# Altered Osteoblast Metabolism with Aging Results in Lipid Accumulation and Oxidative Stress Mediated Bone Loss

**DOI:** 10.14336/AD.2023.0510

**Published:** 2024-04-01

**Authors:** Ananya Nandy, Alison Richards, Santosh Thapa, Alena Akhmetshina, Nikita Narayani, Elizabeth Rendina-Ruedy

**Affiliations:** ^1^Department of Medicine, Division of Clinical Pharmacology, Vanderbilt University Medical Center, Nashville, TN, 37232, USA.; ^2^Molecular Physiology and Biophysics, Vanderbilt University, Nashville, TN, 37232, USA.; ^3^Gottfried Schatz Research Center, Molecular Biology and Biochemistry, Medical University of Graz, Neue Stiftingtalstrasse 6/6, 8010 Graz, Austria

**Keywords:** osteoporosis, lipid droplet, bioenergetics, lipid, aging

## Abstract

Cellular aging is associated with dysfunction of numerous tissues affecting multiple organ systems. A striking example of this is related to age-related bone loss, or osteoporosis, increasing fracture incidence. Interestingly, the two compartments of bone, cortical and cancellous or trabecular, rely on different mechanisms for development and maintenance during ‘normal’ aging. At a cellular level, the aging process disturbs a multitude of intracellular pathways. In particular, alterations in cellular metabolic functions thereby impacting cellular bioenergetics have been implicated in multiple tissues. Therefore, this study aimed to characterize how metabolic processes were altered in bone forming osteoblasts in aged mice compared to young mice. Metabolic flux analyses demonstrated both stromal cells and mature, matrix secreting osteoblasts from aged mice exhibited mitochondrial dysfunction. This was also accompanied by a lack of adaptability or metabolic flexibility to utilize exogenous substrates compared to osteoblasts cultured from young mice. Additionally, lipid droplets accumulated in both early stromal cells and mature osteoblasts from aged mice, which was further depicted as increased lipid content within the bone cortex of aged mice. Global transcriptomic analysis of the bone further supported these metabolic data as enhanced oxidative stress genes were up-regulated in aged mice, while osteoblast-related genes were down-regulated when compared to the young mice. Collectively, these data suggest that aging results in altered osteoblast metabolic handling of both exogenous and endogenous substrates which could contribute to age-related osteoporosis.

## INTRODUCTION

Bone is one of the hardest tissues in the body which very often is mistakenly considered inert. However, it is an incredibly dynamic tissue [1]. Bone creates the skeletal structure of our body while protecting and supporting soft tissues. In this capacity it is constantly combating external forces to maintain homeostasis between the formation of new bone and resorption of old bone [2, 3]. This balance can get disrupted by multiple diseases like osteogenesis imperfecta [4, 5], osteoporosis [6], hyperthyroidism [7] or physiological conditions like menopause [8], diabetes [9], aging [10], leading to impaired skeletal quality and increasing the incidence of fracture.

Loss of bone mass with age, or age-related osteoporosis, is a very common phenomenon which occurs in humans. Interestingly, in mice aging is primarily associated with the decline of trabecular or cancellous bone [11], while varying responses have been noted in the cortical bone compartment. Few reports suggest aging leads to increase in total bone area, medullary area, and cortical density and a decrease in cortical thickness of long bone [11] whereas another shows that cortical tissue mineral density (Ct. TMD) also increases with age [12]. This report further shows cortical thickness of vertebra also increases with age. Age-related osteoporosis has been associated with multiple factors like osteoblastic dysfunction [13], accumulation of reactive oxygen species (ROS) and increased oxidative stress in bone cells [14, 15], reduced number of osteoblasts[16],along with an increase in bone marrow fat [17, 18]. Several reports suggest aging results in reduced osteoblast differentiation capacity while shifting bone marrow stromal cells more towards an adipogenic lineage [19]. Shorter lifespan of matured osteoblasts due to apoptotic cell death is another reason for loss of osteoblasts with age [20, 21]. These mechanisms can lead to reduced bone formation due to lack of osteoblasts and/ or impaired function.

Aging has been associated with the redistribution of fat in several tissues which normally do not accumulate fat [22], where, there is ectopic lipid storage in organs such as the pancreas [23], skeletal muscle [24], heart [25], bone marrow [26, 27] along with a concomitant decrease in subcutaneous and visceral adipose depot. Expansion of bone marrow adipose tissue (BMAT) has also been described with aging [28]. While these phenomena of ectopic lipid accumulation during aging are not fully understood, higher levels of oxidized fatty acid have been detected in aged bone marrow [19, 29] which could influence osteoblast metabolism and function. However, no studies to date have investigated how aging impacts osteoblast metabolism. Therefore, in this study we have aimed to understand how osteoblast metabolism is altered in aged mice. We have compared the bioenergetic profile of *ex vivo* differentiating osteoblasts from 2-month-old mice, which are expected to be within their active phase of skeletal acquisition, with those from aged, 22-month-old mice We report here, that like the ectopic lipid storage in other organs, aging results in the accumulation of lipid content in the bone cortex as well. Interestingly, we also observed bone marrow stromal cells (BMSCs) from the aged mice were loaded with neutral lipids, whereas lipid accumulation slowly progressed during osteoblast differentiation in young mice. Additionally, we demonstrate vastly different metabolic profiles between young vs. aged osteoblasts at both transcript and activity level, which is expected to be contributing to the age-related impairment in bone formation.

## MATERIAL AND METHODS

### Mice

Young, 2-month-old, C57BL/6J male mice were purchased from Jackson Laboratory (stock 000664), while aged (16-month-old) male C57BL/6J mice were obtained from the National Institute of Aging (NIA) Colony and further aged to 22 months in house. Mice were maintained in a clean environment in accordance with guidelines of the Institutional Animal Care and Use Committee (IACUC). During maintenance, the mice were kept under standard 12 hours light/ dark cycle and had *ad libitum* access to standard chow diet and water.

### Micro-computed Tomography (μCT)

Tibia from the mice were cleaned of soft-adhering tissue, placed in 10% neutral-buffered formalin (NBF) for 48 hrs, and then stored in 70% ethanol. The proximal and mid-diaphysis of these tibiae were imaged using an *ex vivo* micro-computed tomography (μCT) scanner (μCT 50, Scanco Medical AG, Brüttisellen, Switzerland). Peak X-ray tube intensity and current of 70 kVp and 114 mA, respectively, 500 projections per full rotation of the sample, and an integration time of 300 ms, image stacks with an isotropic voxel size of 6 μm were acquired for the tibia metaphysis and diaphysis. Trabecular bone was analysed by identifying a region of interest (ROI) 180 μm distal from the proximal tibia growth plate to include a region of secondary spongiosa extending distally of 1.2 mm. Cortical bone was analysed to include 1.2 mm ending at the tibia-fibular junction.

### Isolation of bone marrow stromal cells or haematopoietic stem cells and culturing osteoblasts

Primary murine bone marrow stromal cells (BMSCs) and haematopoetic stem cells (HSCs) were isolated as previously described [30]. Briefly, after removing adherent soft tissue from femur, tibia and iliac crests, their distal and proximal ends were opened. Total bone marrow was isolated by centrifugation at 12,000 RPM for 30 seconds and was plated in complete α-MEM (α-MEM (Sigma; M0450), 10% FBS (Avantor; 89510-186), 1% penicillin/ streptomycin (Sigma; P4333)). Cells were incubated at 37°C in presence of 5% CO_2_. According to the plastic adherence theory, the adherent mesenchymal stromal cells or BMSCs adhered to the plastic flask, while the non-adherent population was used for HSCs [31, 32]. The adherent BMSC population was trypsinized (Sigma; T4049) after 48 hours, counted and plated in appropriate tissue-culture treated plates at appropriate numbers. BMSCs were then cultured in osteogenic medium (complete α-MEM, 50 µg/mL ascorbic acid (Sigma; A4544), and 5 mM β-glycerol phosphate (Sigma; G9422)) to induce osteoblast differentiation after the cells become 80% confluent [30].

For culturing HSCs, the non-adherent cell populations were isolated by centrifugation (2,500 x *g*) of the supernatant. These cells were then counted and seeded accordingly in complete DMEM media (DMEM (Sigma; D64290), 10% FBS, 1% penicillin/ streptomycin) to culture them at 37°C in presence of 5% CO_2_.

### Seahorse Flux Assays

ATP rate assay: To measure metabolic flux in real time by different seahorse based assays, BMSCs were plated in Seahorse XFe 96-well plates at 2.5 × 10^4^ cells/well and cultured under osteogenic conditions for either 0, 3, or 8 days, as we have previously demonstrated these timepoints to be reflective of an immature stromal cell, committed osteo-progenitor, or mature, matrix secreting osteoblast, respectively [33]

At specified timepoints, ATP rate assay was performed. Briefly, after removing cell culture media the cells were washed with basal assay DMEM medium (Agilent; 103575-100) in the absence or presence of 10mM glucose (Agilent; 103577-100), 2mM glutamine (Agilent; 103579-100), 1mM sodium pyruvate (103578-100), 200 nM insulin and 60µM oleic acid-BSA (Sigma; O3008). During the assay, a final concentration of 2 µM oligomycin A (Sigma; 75351) and 1 µM rotenone/1 µM antimycin A (Sigma; R8875, A8674) were injected while oxygen consumption rates (OCR) and extracellular acidification rates (ECAR) were monitored in real time. Considering the stoichiometry of the glycolytic pathway, the percentage of glycolysis is calculated mainly based on the ECAR. Conversely, the rate of oxygen consumption that is coupled to ATP production during oxidative phosphorylation can be calculated as the OCR that is inhibited by addition of the ATP synthase inhibitor, oligomycin.

Mito fuel flex assay: To measure flexibility and dependency of the cells on different nutrients, Mito fuel flexibility assay was done. Three different inhibitors were used independently or as a mixture of two: 2µM UK5099 (2-cyano-3-(1-phenyl-1H-indol-3-yl)-2-propenoic acid) (PZ0160), 3µM BPTES (Bis-2-(5-phenylacetamido-1,3,4-thiadiazol-2-yl) ethyl sulfide) (Sigma; SML0601), and 4µM Etomoxir (Sigma; E1905). This assay determines the rate of oxidation of these three different fuels by measuring mitochondrial respiration via OCR of the cells in the presence or absence of fuel pathway inhibitors. Based on the sequential introduction of these inhibitors, mitochondrial substarte utlization can be interrogated. demand.

Mito Stress assay: To measure the mitochondrial ATP production and proton leak of the cells, Mito stress assay was performed. At the timepoints indicated, according to the manufacturers protocol (Agilent; 103015-100) with minor differences listed previously. Briefly after removing cell culture media the cells were washed with basal assay DMEM medium and assays were performed in the basal media or in presence of cocktail of external nutrients of 10mM glucose, 2mM glutamine, 1mM sodium pyruvate, 200nM insulin and 60µM oleic acid in the basal media. During the assay, sequential injections of 2µM oligomycin A, 2µM FCCP (Sigma; C2920), 1µM rotenone/ 1µM antimycin A mixture inhibitors are used to efficiently analyze the effect on mitochondrial function and calculate proton leak and mitochondrial ATP production.

For all Seahorse assays, Hoechst 33342 stain (Thermo Scientific; 62249) was also injected in the last port and a Cytation 5 (BioTek) was used to provide cell counts, both for normalization and to monitor proliferation throughout differentiation.

### Immunostaining, imaging, and analysis

BMSCs or HSCs were seeded on collagen treated glass coverslips at a density of 1.75× 10^5^/mL and grown in presence of osteogenic media or in DMEM with 10% FBS. BMSCs in osteogenic media were fixed at the mentioned time points whereas HSCs were fixed after 3 days using neutral buffered, methanol-free 4% formaldehyde for 20 minutes. Cells were washed thrice in 1X phosphate buffered saline (PBS) and then were stained for lipid droplets using 10µM BODIPY 493/503 (Thermo Fisher; D-3922) solution for 1 hour at room temperature followed by three more washes with 1X PBS. For Runx2 staining, the cells were blocked and permeabilized in 0.2% Gelatin B (Sigma; G-9391) with 0.1% Saponin (Sigma; 47036) in PBS for 10 minutes after fixing. Immunostaining with Runx2 (Cell Signalling Technology; D1L7F) was done overnight in 0.2% Gelatin B with 0.01% Saponin overnight at 4°C. After washing three times with PBS, we incubated cells with secondary antibody (Invitrogen; Alexa Fluor Goat Anti- Rabbit 647; A32733) in the same buffer for 1 hour at room temperature. Finally, the cells were washed thrice in PBS and once in distilled water then mounted on slide. The coverslips were mounted on slides using the Prolong Glass Antifade Mountant with NucBlue (Thermo Fisher; P36981). For secondary antibody control staining of BMSCs exact same protocol was followed except adding Runx2 in the immunostaining buffer. Confocal Z stacks with 0.30 mm thickness were taken using Zeiss LSM 880. The percentage of Runx2 positive cells were measured with respect to total number of cells in one field. Total number of cells were counted based on DAPI staining. Lipid droplets were identified and counted using built in object identification program in Gen5 software. The same program was used to measure the intensity of these identified lipid droplets.

### Fluorescent fatty acid pulse chase and lipolysis assay

Cells seeded on 6 well plates at a density of 5×10^5^/mL were incubated with complete α-MEM media containing 2 μM BODIPY 558/568 C_12_ (Thermo Fisher; D3835) for 16 h on the indicated time points during osteo-blastogenesis. Cells were then washed three times with 1X PBS and chased for 6 h in α-MEM media with 2% fatty acid free BSA (Sigma; A8806) and 1% penicillin/ streptomycin with 10µM Triacsin C (fatty acyl CoA synthetase inhibitor). The supernatant was collected and harvested for lipid extraction. Lipid was extracted following Bligh and Dyer method [34]. Briefly, four volumes of chloroform: methanol (1:2) (Sigma; 319988, 179337) was added to the supernatants and the mixture was vortexed. One volume of 50mM citric acid (Sigma; 251275), one volume of distilled water and one volume of chloroform were added sequentially to it and again vortexed. This final mixture was then centrifuged at 10,000 RPM for 10 minutes. The lower organic phase was isolated and dried. The dried lipid extract was resuspended in chloroform: methanol (2:1) mixture and loaded on thin layer chromotography (TLC) (Sigma; 1.05553.0001). Lipids were separated by developing TLC at room temperature in the solvent system of cyclohexane: ethyl acetate (1:2) (Sigma; 34855,650528) [35] and fluorescent lipids were visualised using iBright 1500 (Thermo Fisher). Fiji was used to analyse the fluorescent lipid bands where integrated density of the lipid bands from each sample was normalised with the integrated density for the origin of that sample.

### RNA isolation and NextGen Sequencing

Total RNA was isolated from the flushed femur cortex (devoid of marrow elements) following pulverization and submitted to VANTAGE to gain a deep profile of transcriptional regulation of osteoblast function upon aging relative to lipid metabolism, cell bioenergetics, and bone formation. The VANTAGE Core performed QC analysis of the RNA using the Agilent Bioanalyzer and an RNA Qubit assay. RNASeq libraries were prepared using 200 ng of total RNA and the NEBNext rRNA Depletion Kit (NEB, Cat: E6310X) per manufacturer’s instructions. This kit employs an RNaseH-based method to deplete both cytoplasmic (5S rRNA, 5.8S rRNA, 18S rRNA and 28S rRNA) and mitochondrial ribosomal RNA (12S rRNA and 16S rRNA) from human, mouse, and rat total RNA preparations. The mRNA was enriched via poly-A-selection using oligoDT beads and then the RNA was thermally fragmented and converted to cDNA. The cDNA was adenylated for adaptor ligation and PCR amplified. The libraries were sequenced using the NovaSeq 6000 with 150 bp paired end reads targeting 50M reads per sample. RTA (version 2.4.11; Illumina) was used for base calling and analysis was completed using Dragen RNA Pipeline (v3.7.5).

The raw data was submitted to VANGARD for analysis. Adapters were trimmed by Cutadapt (v2.10). After trimming, reads were mapped to the mouse genome GRCm38.p6 using STAR (v2.7.8a) and quantified by feature Counts (v2.0.2). DESeq2 (v.1.30.1) was used to detect differential expressions between two groups. WebGestaltR (v0.4.4) was used to perform functional enrichment analysis against Gene Ontology and KEGG. GSEA (v4.2.3) was used to find enriched pathways against HALLMARK gene sets in MsigDB (v7.5.1). Differentially expressed genes belonging to metabolic pathways were determined.

### Lipid isolation from bone and TLC analysis

Mouse tibia: Flushed tibia cortex (devoid of marrow elements) was pulverized and used to harvest total lipid by Bligh and Dyer method [34] mentioned previously. However, the powder obtained from total flushed tibia after pulverization was weighed before adding chloroform: methanol (1:2) mixed to it for normalization. Also, the powder was kept overnight in chloroform, methanol mixed in a 37°C water bath for better and complete extraction before the following steps were performed. Finally, the dried lipid extract was resuspended in equal volume of (80 µl) chloroform: methanol (2:1) mixture and loaded on TLC (Sigma; 1.05553.0001). Lipids were separated by developing TLC in 4°C in the solvent system of hexanes: di ethyl ether: acetic acid (70:30:1) (Sigma; 293253, Emparta; 1.07026.2500, Sigma; 695092). The TLCs were stained using 10% (wt/vol) copper sulphate in an 8% (vol/vol) phosphoric acid solution, followed by charring at 120°C for visualization of lipids. Fiji was used to analyse the lipid bands where integrated density of the lipid bands from each sample were normalised with the weight of the powdered bone sample.

Human bone: Cadaveric tissues were stored fresh-frozen and obtained from the Musculoskeletal Transplant Foundation (Edison, NJ, USA), the Vanderbilt Donor Program (Nashville, TN, USA), and the National Disease Research Interchange (Philadelphia, PA, USA). Cortical bone samples were extracted from the proximal quadrant of the femoral mid-shaft of human donors (male donors, aged 65 to 82 years old). Specimens (one per donor) were cut using a circular low-speed, diamond-embedded saw (Model 660; South Bay Technology, Inc., San Clemente, CA, USA) and machined using an end mill to a specimen with 5mm thickness and 1.8mm length. These samples devoid of marrow material were then pulverized, weighed and used to extract lipid by Bligh and Dyer method [34] in a similar manner as that from mouse bone. However, for running TLCs, since the weight of the powdered bones were much higher and variable, the dried lipids were resuspended according to weight of the powdered samples. TLC development and lipid visualization was done in a similar manner as for lipid from mouse bones.

### Statistical Analysis

Statistical analyses were performed in GraphPad Prism V9. Normal distribution assumption for sample size<40 was evaluated using Shapiro-Wilk normality test. Statistically significant differences across two groups were evaluated using Student's two-tailed unpaired *t* test with significance defined as p<0.05 when normal distribution was met when normality assumptions were not met, an alternative non-parametric test (Mann-Whitney test) was used. Data expressed are either mean ± standard deviation (SD) or mean ± standard error of mean (SEM) as described. Micro CT analysis and RNA-sequencing were done with 5 mice from each group, for cell culture experiments stromal cells were obtained and pooled from 6 mice in each group. For the statistical analysis of RNA-sequencing, see “RNA isolation and NextGen Sequencing” above.

## RESULTS

### Trabecular bone is drastically reduced during aging while Cortical bone expands

**Figure 1.**
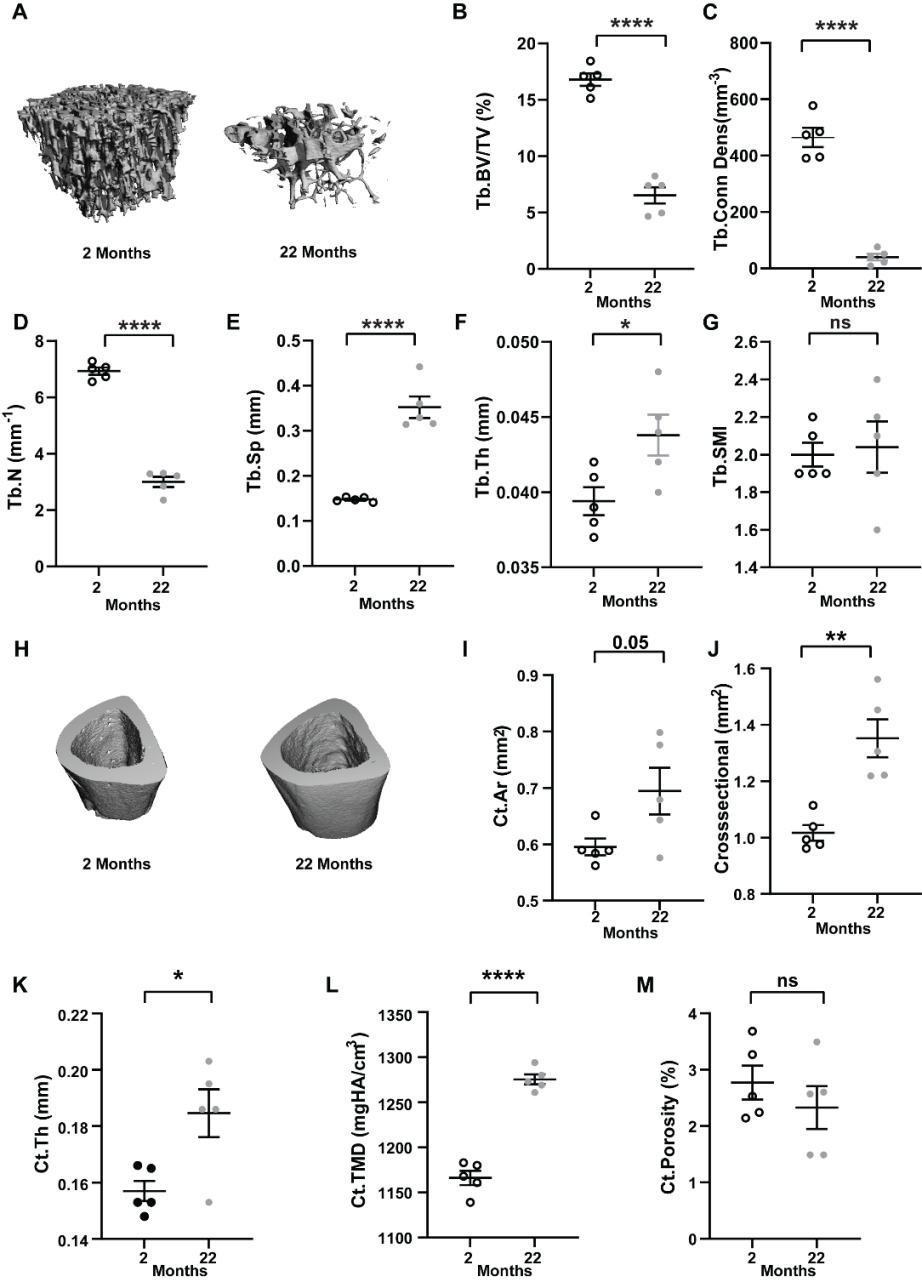
**Age-related changes in bone microarchitecture**. **(A-G)** micro computed tomography (µCT) analysis of trabecular bone; trabecular bone of tibia of 2 months or 22 months old male mice. **(A)** Representative 3D micro CT image of trabecular bone. **(B)** Percentage bone volume over total volume (Tb. BV/TV; %). **(C)** Connection density (Tb. Conn Dens; mm^-3^). **(D)** Trabecular number (Tb. N; mm^-1^). **(E)** Trabecular spacing (Tb.Sp;mm). **(F)** Trabecular thickness (Tb. Th;mm). **(G)** Trabecular structure model index (Tb.SMI;0= plates, 3= rods). **(H-M)** micro computed tomography (µCT) analysis of cortical bone; (H) Representative 3D micro CT image of cortical bone. **(I)** Cortical bone area (Ct.Ar;mm^2^ ). **(J)** Total cross-sectional area (Crosssectional;mm^2^). **(K)** Thickness (Ct. Th;mm). **(L)** Tissue mineral density (Ct.TMD;mgHA/cm^3^) (M) Percentage porosity (Ct.Porosity; %). Each dot represents data from individual animal where N=5 and data are mean ± standard error of mean. t tests or non-parametric Mann-Whitney tests were done accordingly after testing normal distribution using Shapiro-Wilk normality test to determine significance between two groups where *, p <0.05, **, p <0.01, ***, p <0.001, ****, p <0.0001.

In line with previous reports our data also showed drastic loss of trabecular bone in the tibia metaphysis of aged mice (Fig. 1A) quantified by significantly lower BV/TV (6.277% ± 0.48 versus 16.86% ± 0.68) (Fig. 1B), connectivity density (Conn.Dens; 35.98mm^-3^ ±7.72 versus 580.1mm^-3^ ± 151) (Fig. 1C), trabecular number (Tb.N; 2.956mm^-1^ ± 0.13 versus 6.96mm^-1^ ± 0.20) (Fig. 1D), with higher trabecular separation (Tb.Sp; 0.3548mm ± 0.018 versus 0.1477mm ± 0.0038) (Fig. 1E) and trabecular thickness (Tb.Th; 0.0446mm ± 0.0018 versus 0.0395mm ± 0.00067) (Fig. 1F).

The Structure Model Index was similar between the two groups (Tb. SMI; 2.11 ± 0.079 versus 2 ± 0.051) (Fig. 1G). The length of tibia from the older mice was slightly higher than that of younger mice (18.40mm ± 0.069 versus 16.77mm ± 0.68). Interestingly, microCT analyses of the tibia diaphysis revealed older mice had higher cortical area (Ct.Ar; 0.694mm^2^ ± 0.041), total cross-sectional area (Crosssectional; 1.352 mm^2^ ± 0.067) as well as cortical thickness (Ct.Th; 0.1846mm ± 0.0085) compared to young mice (Ct.Ar; 0.595mm^2^ ± 0.014, Crosssectional; 1.014 mm^2^ ± 0.022 and Ct.Th; 0.1583mm ± 0.0032) (Fig. 1H,I,J&K). Although, the cortical tissue mineral density was higher in older mice (Ct.TMD; 1275mgHA/cm^3^ ± 5.57 versus 1166mgHA/cm^3^ ± 7.88) (Fig. 1L) there was no change in percentage porosity per se (Ct.Porosity; 2.32% ± 0.38 versus 2.77% ± 0.30) (Fig. 1M).

### Metabolic profile for energy production in aged and young mice are different during osteoblastogenesis

**Figure 2.**
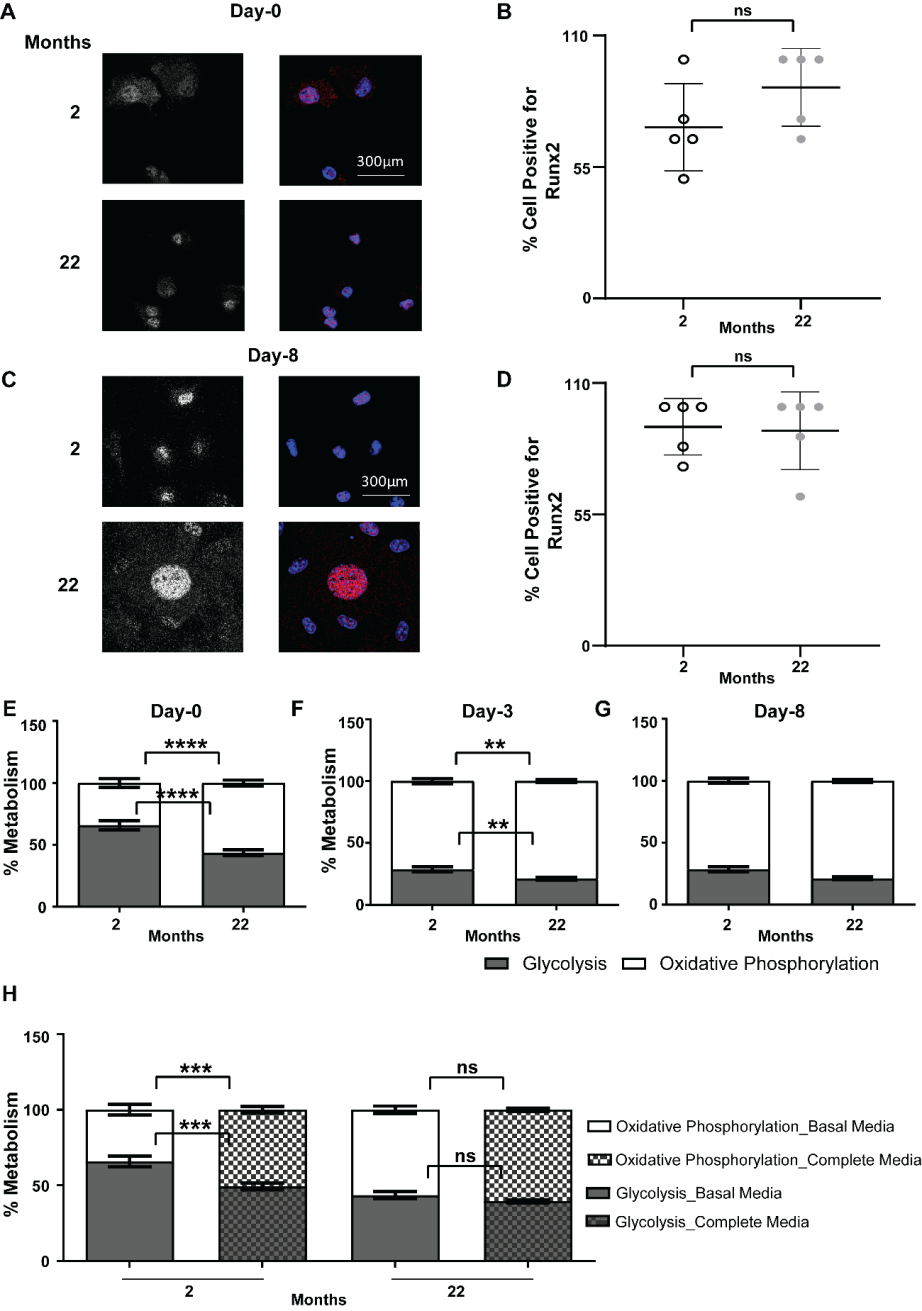
**Effect of aging on cellular metabolic profile**. **(A)** Representative confocal image of undifferentiated stromal cells on 0^th^ day and (C) fully differentiated osteoblasts on 8^th^ day of *ex vivo* differentiation of 2 months or 22 months old mice immunostained for Runx2 along with mounting with DAPI. Panel 1 shows monochrome images of nuclei staining by Runx2 whereas panel 2 shows merged image (Runx2 in red and DAPI in blue). (B, D) Quantification of percentage of Runx2 positive cells in the 2 months (open) and (D) 22 months (light gray) old mice on (B) 0^th^ and (D) 8^th^ day of differentiation. Data are mean ± standard deviation (SD) where percentage of Runx2 positive cells were counted from independent images captured in 5 different field of view of coverslip (n=5) with pooled BMSCs obtained from 6 mice (N=6) in each age group. t tests or non-parametric Mann-Whitney tests were done accordingly after testing normal distribution using Shapiro-Wilk normality test to determine significance between two groups where *, p <0.05, **, p <0.01, ***, p <0.001, ****, p <0.0001. **(E-G)** Percentage of glycolytic (shaded) versus oxidative phosphorylation (white) measured by Seahorse ATP rate assay in absence of any external nutrients during *ex vivo* osteoblastogenesis of stromal cells harvested from 2 months or 22 months old mice in (E) stromal cells on 0^th^ day before addition of differentiation media. **(F)** cells midway in their osteoblastogenesis process on 3^rd^ day of differentiation. **(G)** matured osteoblast on 8^th^ day of differentiation. **(H)** Percentage glycolysis and oxidative phosphorylation measured in stromal cells on 0^th^ day of *ex vivo* differentiation harvested from 2 months or 22 months old male mice by Seahorse ATP rate assay done in presence (with pattern in shaded or open) (Complete media) or absence (without pattern in shaded or open) (Basal media) of additional external nutrients i.e., in complete or basal media respectively. Data are mean ± standard error of mean of data normalized to cell counts per well with data from minimum 17 wells per group (n=17) done from pooled BMSCs obtained from 6 mice (N=6) in each age group. t tests or non-parametric Mann-Whitney tests were done accordingly after testing normal distribution using Shapiro-Wilk normality test to determine significance between two groups where *, p <0.05, **, p <0.01, ***, p <0.001, ****, p <0.0001.

primarily relying on oxidative phosphorylation of endogenous substrates (88.7% ± 0.5 for young mice and 87.7% ± 0.6 for old mice) and with lower glycolytic profile (11.3% ± 0.5 for young and 12.3% ± 0.6 for old mice) (Fig. 2G). Interestingly, addition of exogenous nutrients into the assay media (i.e., mix of glucose, glutamine, pyruvate, and oleic acid) was able to shift the metabolic pathway of cells of young mice from glycolysis (from 65.8% ±3.6 to 49.3% ± 2.3) to more towards oxidative phosphorylation (from 34.2% ±3.6 to 50.7% ± 2.3) on the 0^th^ day of differentiation similar to that of cells from aged mice in absence of those external nutrients (Fig. 2H). Addition of the external nutrients in the assay media was not able to cause any significant alteration in the metabolism of cells from aged mice (Fig. 2H).

### Stromal cells and matured osteoblasts from older mice have higher flexibility for endogenous nutrients for energy generation but lack the ability to adapt to addition of excess external nutrients

**Figure 3.**
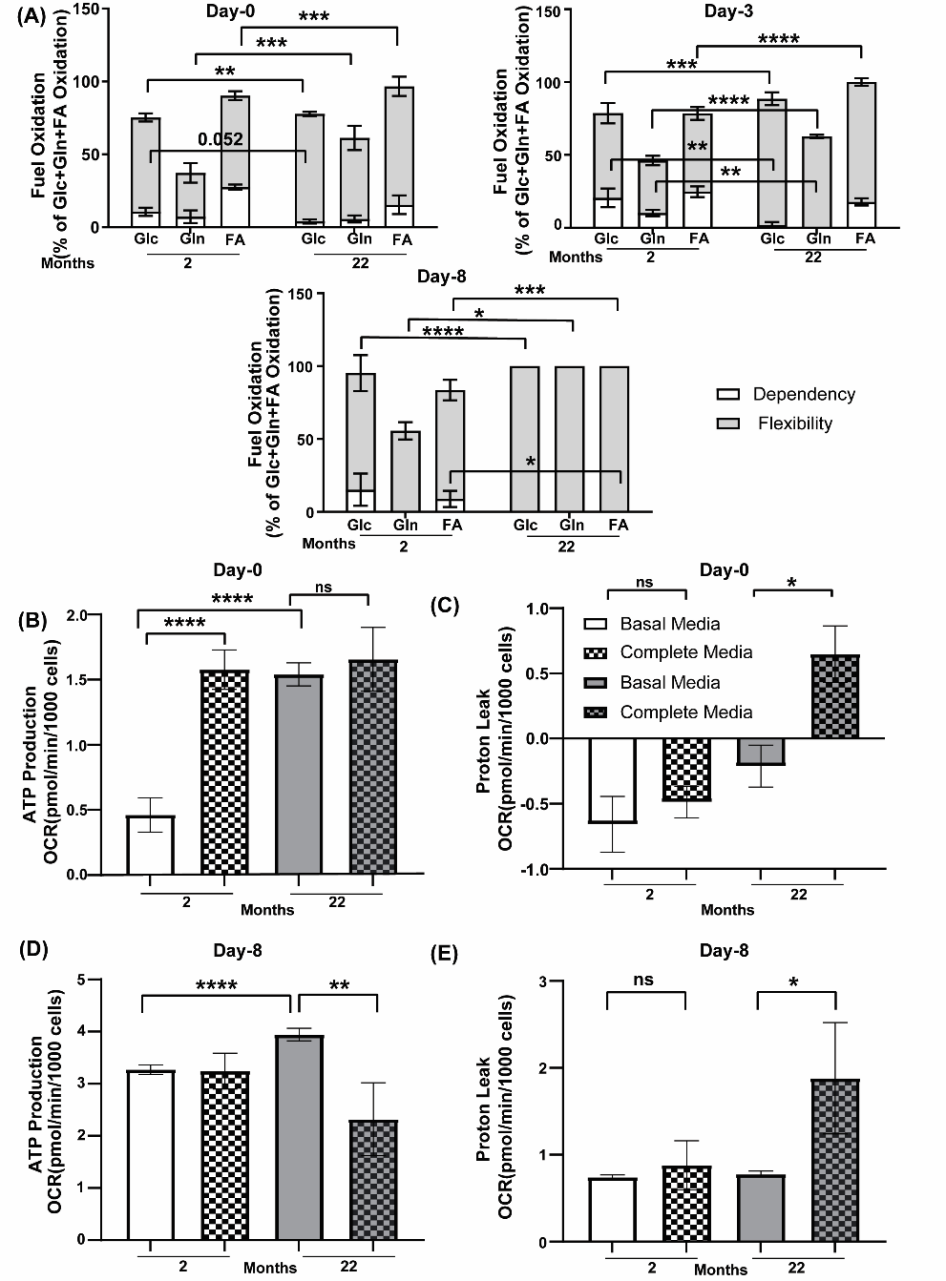
**Effect of aging on flexibility and dependency towards endogenous and exogenous nutrients**. **(A)** Dependency (open) and flexibility (lighter gray shaded) of cells from 2 months or 22 months old mice towards three basic endogenous nutrients glucose/Glc (carbohydrate), glutamine/Gln (amino acid) and fatty acid/FA (lipid) in absence of any external nutrients during *ex vivo* osteoblastogenesis (0^th^, 3^rd^ and 8^th^ day of differentiation) measured by Seahorse Mitoflex assay where data are mean ± standard error of mean of data normalized to cell counts per well with data from minimum 21 wells per group (n=21) done from pooled BMSCs obtained from 6 mice(N=6) in each age group. **(B-E)** ATP production and Proton leak measured by Seahorse Mitostress assay from 2 months(open) or 22 months (gray) old mice in presence (with pattern) (Complete media) or absence (without pattern) (Basal media) of additional external nutrients (B), **(C)** in stromal cells on 0^th^ day of *ex vivo* differentiation. **(D)**, **(E)** in matured osteoblasts on 8^th^ day of differentiation. Data are mean ± standard error of mean of data normalized to cell counts per well with data from minimum 21 wells per group (n=21) done from pooled BMSCs obtained from 6 mice (N=6) in each age group. t tests or non-parametric Mann-Whitney tests were done accordingly after testing normal distribution using Shapiro-Wilk normality test to determine significance between two groups where *, p <0.05, **, p <0.01, ***, p <0.001, ****, p <0.0001.

Stromal cells (day 0) from young mice demonstrated higher dependency on glucose (10.63% ± 2.761) compared to aged mice (4.018% ±1.455), whereas cells from aged mice showed more flexibility for all three nutrients, glucose (73.78% ± 1.45), glutamine (55.58% ± 8.37) and fatty acids (81.18% ±6.12) compared to cells from young mice (64.74% ± 2.76, 30.08% ± 6.66 and 62.68% ± 3.04 respectively) (Fig. 3A). Committed osteoblasts from 3 days in osteogenic medium derived from aged mice were also more flexible towards all three nutrients: glucose (86.63% ± 4.40), glutamine (62.63% ±1.22), and fatty acid (82.16% ± 2.51) compared to the cells from young mice (58.07% ±6.90, 36.02% ±3.27, 53.76% ±4.43 respectively) (Fig. 3A). At maturation (day 8) osteoblasts from the aged mice demonstrate maximum flexibility for all glucose (100% ± 0), glutamine (100% ± 0), and fatty acid (100% ± 0), whereas those from young mice show 80.07% ± 12.35 flexibility for glucose, 55.64% ± 5.90 for glutamine and 74.69% ±7.06 for fatty acid (Fig. 3A).

Interestingly, mature osteoblasts from young mice show more (8.89% ± 5.55) dependency on fatty acid (Fig. 3A). Given these differences in metabolism and nutrient dependency between old vs. young mice, we next investigated their mitochondrial respiration. Mitostress assay showed the cells from older mice were able to produce much higher ATP utilizing endogenous nutrients during both initial (1.541 ±0.0884 versus 0.4621 ± 0.1317) and last phase of differentiation (3.944 ± 0.1218 versus 3.271 ±0.088) (Fig. 3B and 3D). However, the difference between the two age groups decreases at later stages in osteogenic differentiation where the matured osteoblast from young mice almost catches up with the ATP production of older mice using endogenous substrate (Fig. 3D). Interestingly, the older mice were not able to adapt to addition of external nutrients as well as the younger mice. When external nutrients were added during early phase (0^th^ day) the younger mice were able to utilize them and produce more ATP (1.578 ± 0.1514 versus 0.4621 ±0.1317) whereas the ATP production by the older mice remained unaltered (1.656 ± 0.2456 versus 1.541 ± 0.0884) (Fig. 3B). However, this addition caused an increase in the proton leak in older mice (0.6470 ± 0.2177 versus -0.2119 ± 0.1609) (Fig. 3C). Addition of external nutrients were not able to affect either the ATP production (3.243 ± 0.3438 versus 3.271 ± 0.088) or the proton leak (0.8787 ± 0.2835 versus 0.7415 ± 0.0278) of younger mice at day 8 whereas it caused a decrease in ATP production (2.319 ± 0.6994 versus 3.944 ± 0.1218) and increase in proton leak (1.880 ± 0.6401 versus 0.7765 ± 0.0345) in older mice (Fig. 3D and 3E).

### Stromal cells from aged mice have higher cellular nutrient availability in form of stored lipid droplets to utilize for oxidative phosphorylation during early phase of differentiation

**Figure 4.**
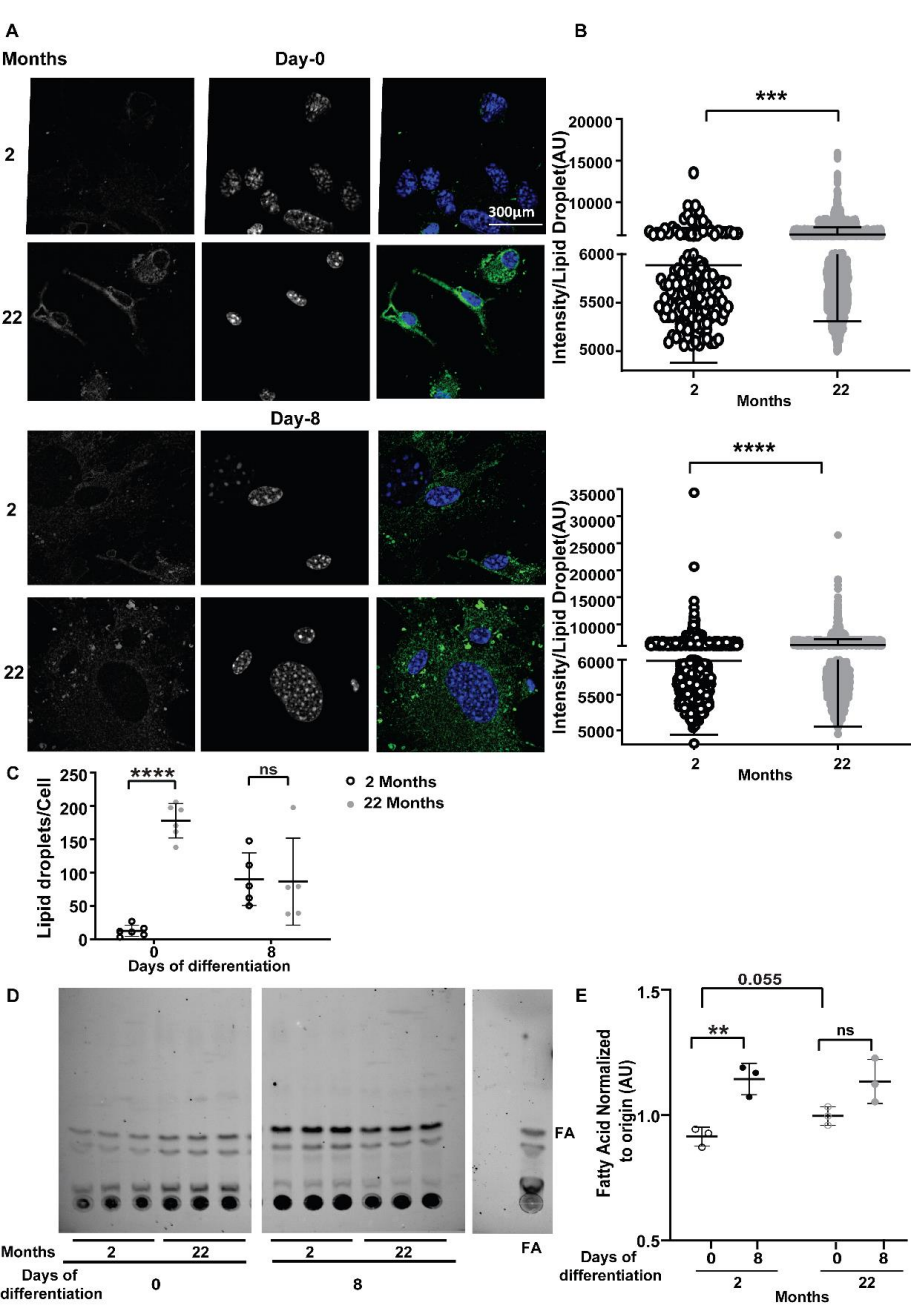
**Effect of aging on lipid storage and metabolism in osteoblasts**. **(A)** Representative confocal image of undifferentiated stromal cells on 0^th^ day and fully differentiated osteoblasts on 8^th^ day of *ex vivo* differentiation of 2 months or 22 months old mice. Cellular lipid droplets were stained with BODIPY 493/503 (green in merged panel) and nuclei were stained with DAPI (blue in merged panel). Panel 1 and 2 show monochrome images of lipid droplet and nuclei staining respectively whereas panel 3 shows merged image. Quantification of (B) the intensity of BODIPY 493/503 per lipid droplet in 2 months (open circle) or 22 months (gray closed circle) where each dot represents intensity of one lipid droplet. Data are mean ± standard error of mean where intensity of each lipid droplets were measured from independent images captured in 5 or 6 different field of view of the coverslip (n=5/6) with pooled BMSCs obtained from 6 mice (N=6) in each age group.t tests were done assuming normal distribution since, data points were more than 40, to determine significance between two groups where *,p <0.05, **,p <0.01, ***,p <0.001, ****, p <0.0001 (C) number of lipid droplets per cell in the two groups on 0^th^ and 8^th^ day of differentiation. Data are mean ± standard deviation (SD) where lipid droplets per cell were counted from independent images captured in 5 or 6 different field of view (n=5/6) from pooled BMSCs obtained from 6 mice (N=6) in each age group. The number of lipid droplets counted from each image were divided by number of cells (number of DAPI positive nucleus) in that image to get lipid droplets per cell. t tests or non-parametric Mann-Whitney tests were done accordingly after testing normal distribution using Shapiro-Wilk normality test to determine significance between two groups where *, p <0.05, **, p <0.01, ***, p <0.001, ****, p <0.0001. **(D)** Thin layer chromatogram of lipid harvested from BODIPY 558/568 C_12_ (red fluorescent fatty acid) labelled stromal, or *ex vivo* differentiated matured osteoblast cells from 2 months or 22 months old mice, along with the chromatogram of BODIPY 558/568 C_12_ fatty acid ran as the standard. The bands below fatty acid are degraded products of the labelled fatty acid (E) Densitometric quantification of fatty acid normalized to origin. Data are mean ± standard deviation done from 3 wells (n=3) with pooled BMSCs obtained from 6 mice in each age group (N=6). t tests or non-parametric Mann-Whitney tests were done accordingly after testing normal distribution using Shapiro-Wilk normality test to determine significance between two groups where *, p <0.05, **, p <0.01, ***, p <0.001, ****, p <0.0001.

To determine potential endogenous fuel sources for oxidative phosphorylation, we imaged osteoblasts throughout differentiation for neutral lipid droplets. BODIPY 493/503 staining was done to measure the total neutral lipid component of these cells. Total neutral lipid was higher in cells from older mice during both early (6141 AU ± 15.59 on 0^th^ day) and late phases (6144 AU ± 16.72 on 8^th^ day) of osteoblast differentiation compared to that of young mice (5887 AU ± 79.35 and 5984 AU ± 22.65 respectively) (Fig. 4A and 4B). Also, the number of lipid droplets per cell were significantly higher during early phase of differentiation in the cells from aged mice (177.9 ± 25.88 versus 12.78 ± 8.29) (Fig. 4C). Even the matured osteoblasts from the old mice on 8^th^ day of differentiation shows a higher trend for intracellular lipid droplet number, although not statistically significant (Fig. 4C). Furthermore, BMSCs isolated from young mice demonstrate increased lipolysis occurring in response to osteoblast differentiation as evidenced by higher amounts for fatty acid detected in the supernatant during later time point of differentiation (from 0.9148 ± 0.037 AU at day 0 to 1.144 ± 0.062 AU at day 7) (Fig. 4D and 4E). Although, this increased release of fatty acids was not detected aged mice as osteoblast matured (Fig. 4D and 4E), interestingly stromal cells from aged mice showed more trend towards lipolysis compared to cells from younger mice (0.9969 ± 0.037AU versus 0.9148 ± 0.037AU).

### Long bones of aged mice are rich in neutral lipid content

**Figure 5.**
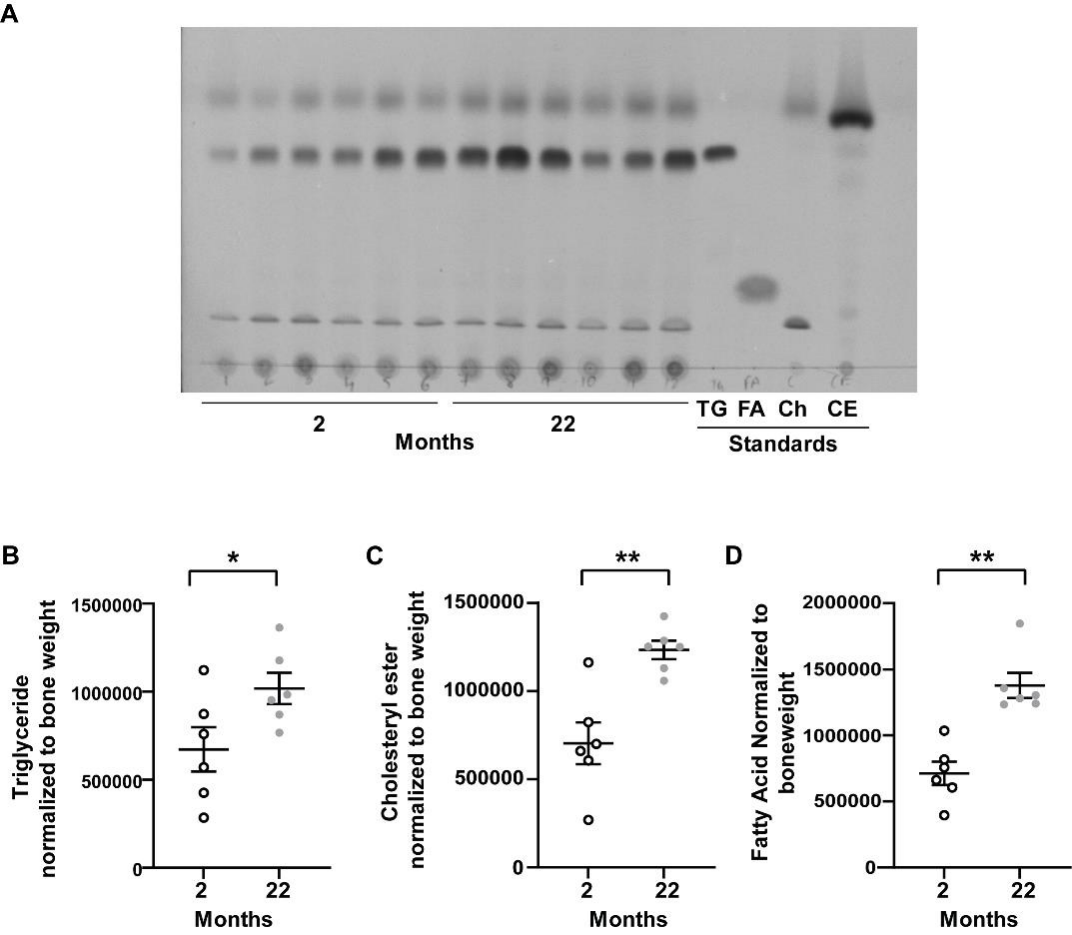
**Lipid profile in bone cortex from young or aged mice**. **(A)** Thin layer chromatogram (TLC) of lipid harvested from flushed tibia (tibia without any bone marrow) from 2 months or 22 months old mice. Standards include triglycerides (TG), fatty acids (FA), cholesterol (Ch), and cholesteryl esters (CE). **(B-D)** Densitometric quantification of the lipid species from 2 months (open circle) and 22 months (gray closed circle) old mice from the chromatogram (B) triglyceride, **(C)** cholesteryl ester (D) fatty acid normalized to bone weight. Each dot represents data from individual animal where N=6 animals in each group and data are mean ± standard error of mean. t tests or non-parametric Mann-Whitney tests were done accordingly after testing normal distribution using Shapiro-Wilk normality test to determine significance between two groups where *, p <0.05, **, p <0.01, ***, p <0.001, ****, p <0.0001.

To find out whether with age, *in-vivo* also the bone cells get loaded up with lipid we performed lipid extraction from flushed bone (tibia) i.e, from bone without bone marrow. Both the storage lipids, triglyceride, and cholesteryl ester were respectively 1.5 and 1.7 times higher in bones from aged mice (Fig. 5A, B and C). Also, free fatty acids were 1.9 times higher (Fig. 5D) in long bones of old mice compared to young mice. We also extracted lipids from flushed femur from human autopsy samples to do lipid profiling. We found human bone cortex to contain both storage neutral lipid esters triglyceride and cholesteryl ester as well as fatty acid (Supplementary Fig. 2). We observed variation between individuals in terms of presence of different lipid components. To our knowledge, here we have reported for the first-time lipid profiling from human bone cortex.

**Figure 6.**
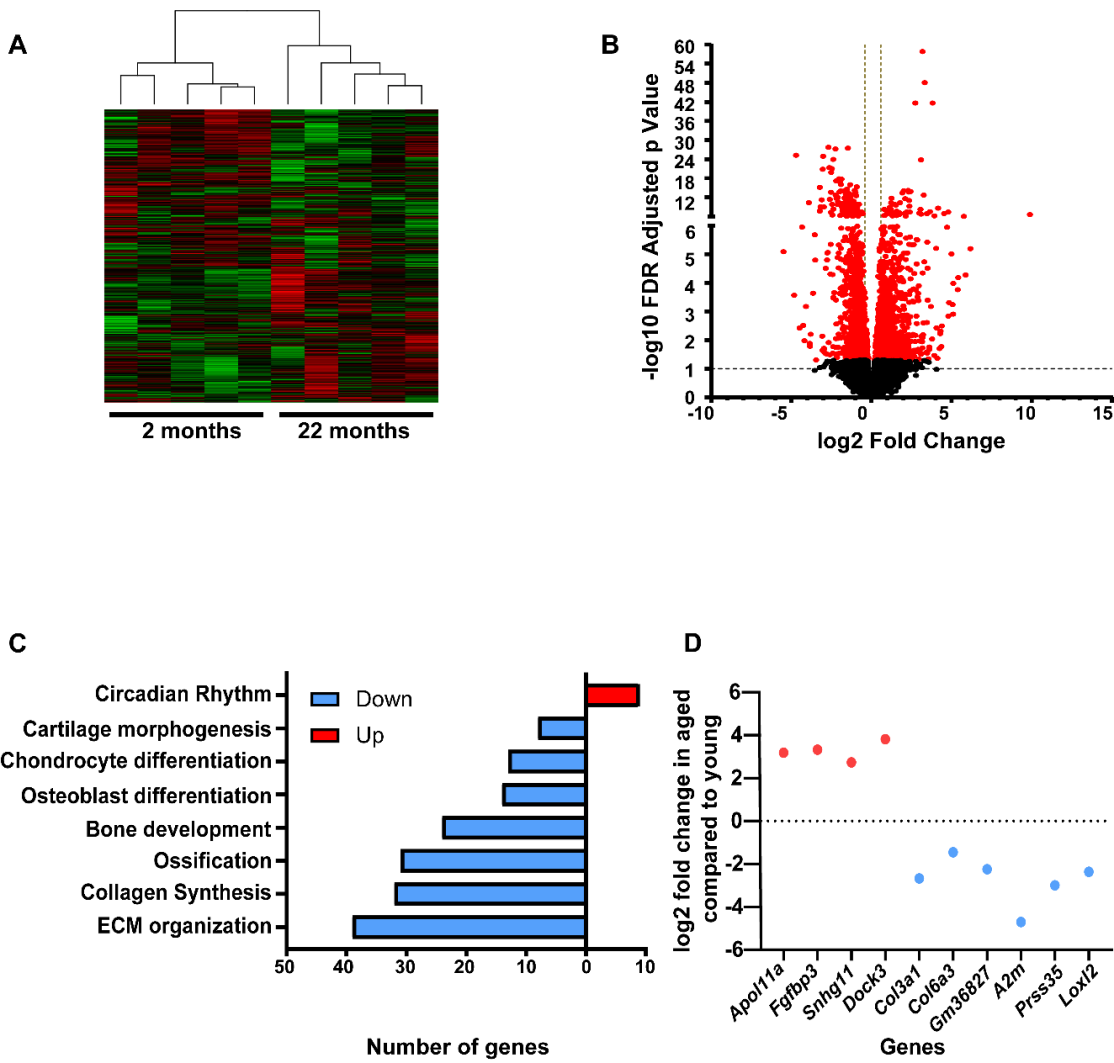
**Transcriptional profiling and analysis of differentially expressed genes in bones with aging**. **(A)** Correlation heatmap using 25% variant gene. **(B)** Volcano plot showing differentially expressed genes (FDR adjusted p <0.05 in red circles) in flushed femur of 2 months versus 22 months old mice in pairwise comparison. **(C)** Top enriched differentially expressed pathways based on functional enrichment analysis (D) log2 fold change of top 10 differentially expressed genes.

### Global transcriptome profiling of long bone from young and aged mice reveals differential metabolic profile and oxidative stress mitigation property

Global transcriptome analysis of flushed femur (bone without marrow) was done by RNA sequencing to do a comparative study of gene expression in young versus aged mice. The RNA after sequencing was subjected to analysis for differentially expressed genes (at FDR adjusted p value/ q value ≤ 0.05). We combined 5 mice data from each group to get in average 0.85 billion reads from young mice and 0.92 billion reads from aged mice (Supplementary Table 1). A correlation heat map using 25% variant genes demonstrates similarity of gene expression among the replicates belonging to the same group (Fig. 6A). Out of the differentially expressed genes between the two groups, 994 genes were found to be upregulated and 817 genes were downregulated in old mice compared to the young with a cut off 1.5fold up and down regulation (Fig. 6B and Supplementary Table 2).

**Figure 7.**
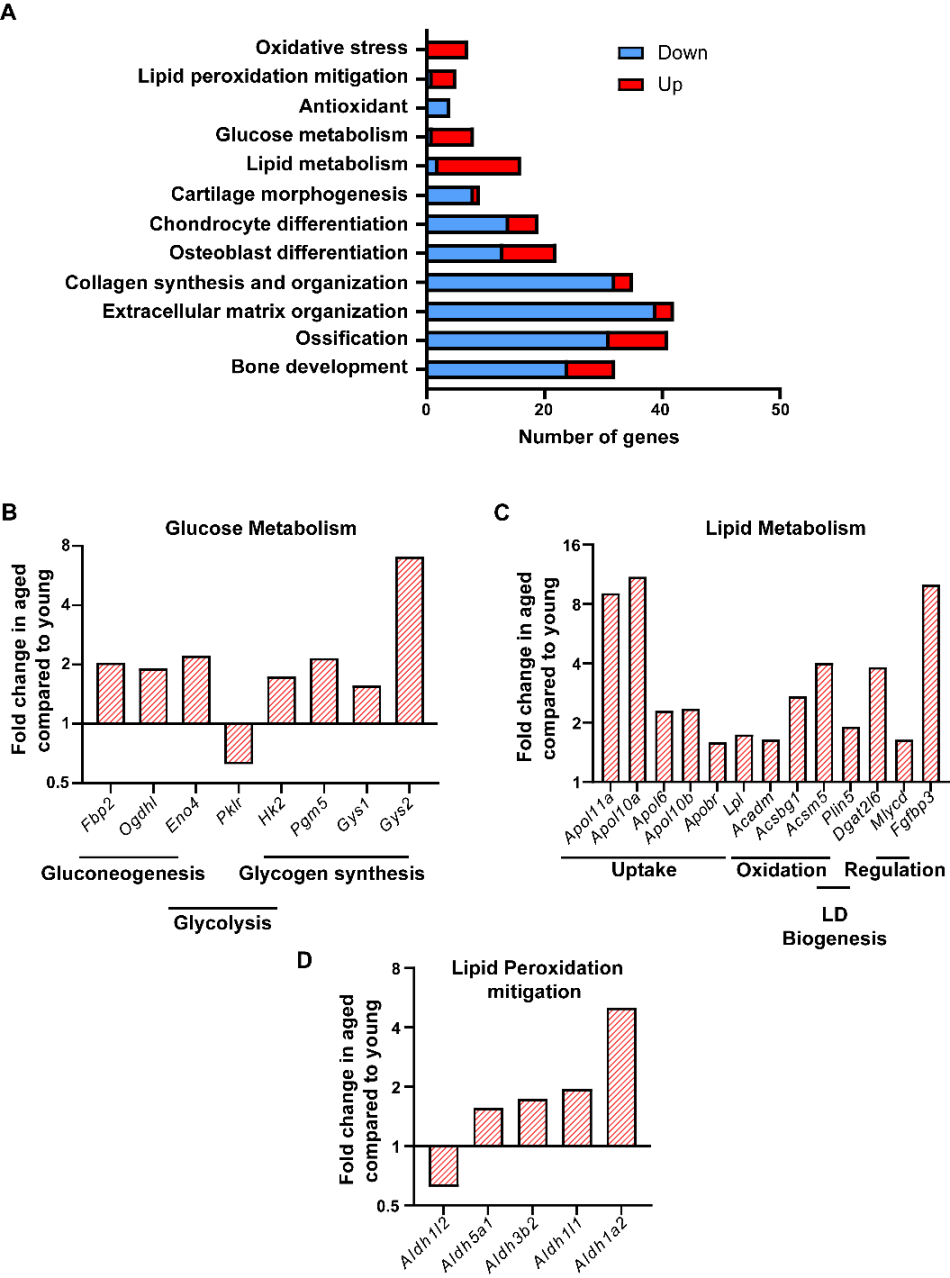
**Aging alters expression of genes involved in metabolic processes**. **(A)** Distribution of differentially expressed genes belonging to bone formation or metabolic pathways. **(B)** Fold change expression of genes involved in glucose metabolism, **(C)** Lipid metabolism (LD or lipid droplets), **(D)** Lipid peroxidation mitigation.

Significant genes were classified based on their involvement in different functional pathways. As expected, genes involved in bone development (24), ossification (31), and osteoblast differentiation (13) were the pathways having maximum number of genes expressed in lower abundance in the bones of aged mice compared to young mice (Fig. 6C). Additionally, the old mice exhibited downregulation of expression of genes involved in chondrocyte differentiation (14) and cartilage morphogenesis (8) (Fig. 6C). Interestingly, genes expressing enzymes involved in collagen synthesis and organization, along with extracellular matrix (ECM) organization were most affected with aging (32 and 39 genes, respectively) (Fig. 6C). Out of all the enriched differentially expressed pathways only the circadian entrainment pathway had a greater number of genes being upregulated in aged mice than in young ones (9) (Fig. 6C). Out of the top 10 differentially expressed genes, 2 genes involved in lipid metabolism (*Apol11a* and *Fgfbp3*) were upregulated whereas 4 genes involved in ECM formation and organization were downregulated (*Col3a1, Col6a3, Prss35, Loxl2*) in aged mice (Fig. 6D). The maximum number of differentially expressed genes belonged to the category of ECM organization (39 downregulated and 3 upregulated) most of which are associated with collagen synthesis including *Col9a1,Col3a1,Col12a1,Loxl2, Col11a1,Col10a1,Col16a1,Lox,Col5a2,Col8a1,Col5a1,Col1a2,Col1a1,Col15a1,Col8a2,Col4a1,Col5a3,Col4a2,Col24a1,Col11a2,Loxl1,Col14a1,Col4a3,Col4a5* (Fig. 7A and Supplementary Table 2).

Oxidative stress marker genes were also found to be upregulated (7) along with downregulation of 4 oxidative stress mitigation genes/ antioxidants and upregulation of 4 lipid peroxidation mitigation genes (Fig. 7A and Supplementary Table 2). In terms of glucose metabolism, we found the gene coding enzyme for the last rate limiting step in glycolysis Pyruvate kinase (*Pklr*) to be downregulated (1.6-fold) whereas the gene for the first-rate limiting enzyme, Hexokinase (*Hk2*) to be upregulated (1.7-fold). Interestingly, 3 genes involved in gluconeogenesis including one of the rates limiting Fructose-1,6- bisphosphatase (*Fbp2*) were upregulated (2-fold) (Fig. 7B and Supplementary Table 2). Genes for enzymes of glycogen synthesis pathway *Pgm5, Gly1* and *Gly2* were also upregulated (2, 1.5 and 7-fold respectively) (Fig. 7B and Supplementary Table 2). A total of 16 genes involved in lipid metabolism were differentially expressed, of which 14 were upregulated and 2 downregulated (Fig. 7C and Supplementary Table 2) whereas 4 of the lipid peroxidation mitigation genes belonging to aldehyde dehydrogenase category were upregulated (Fig. 7D and Supplementary Table 2). Out of the upregulated genes of lipid metabolism category 6 were involved in uptake of fatty acids, 3 in β-oxidation and 2 in regulation of lipid metabolism (Fig. 7C and Supplementary Table 2). Another upregulated gene *Dgat2l6* is involved in lipid droplet biogenesis whereas *Plin5* has a dual role in lipogenesis as well as oxidation (Fig. 7C and Supplementary Table 2).

**Figure 8.**
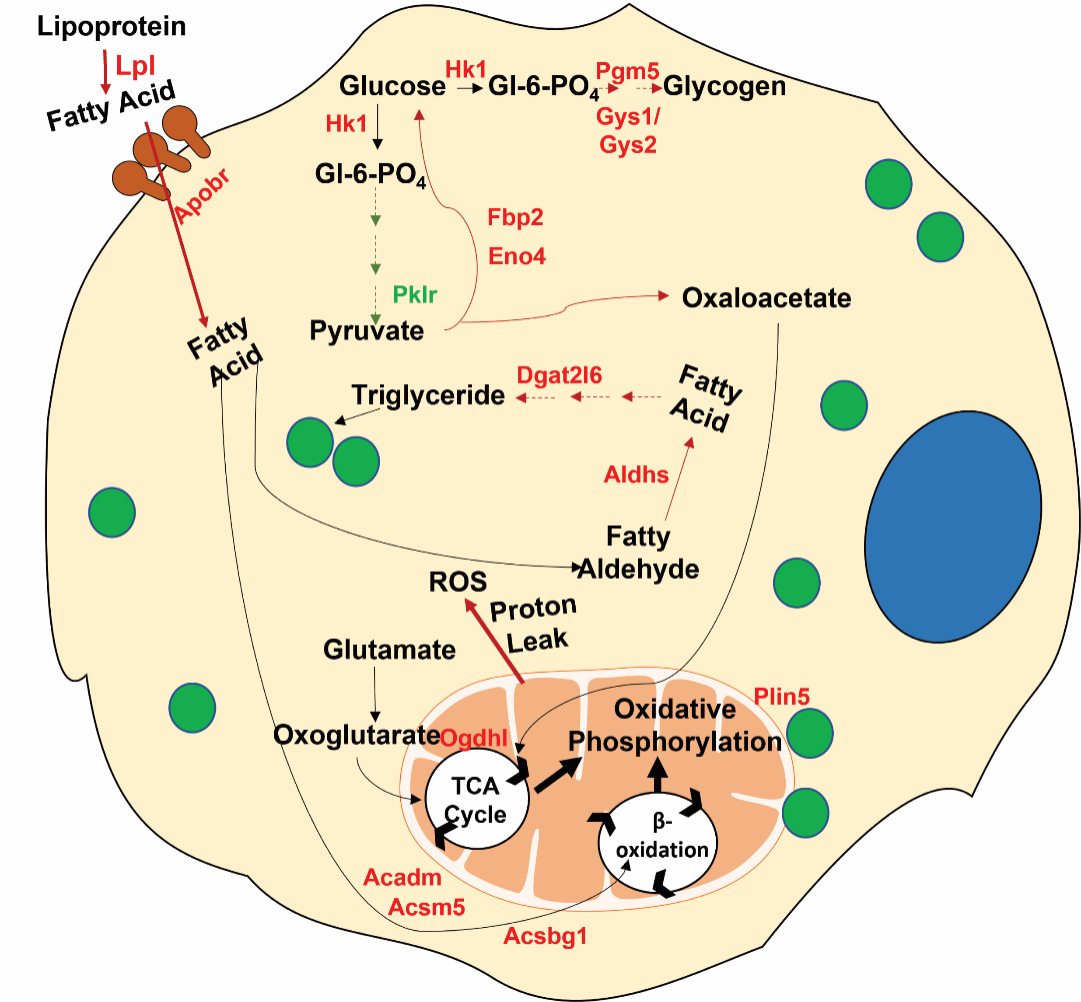
**Image depicting metabolic alterations occurring in osteoblasts contributing to age-related bone loss**. Genes upregulated in bones of aged mice compared to young mice are shown in red whereas those downregulated in green. The pathways upregulated are shown in red and downregulated in green dashed line arrows whereas one step reactions are shown with solid line arrows. Blue circle: Nuclei; Green circles: Lipid droplet.

## DISCUSSION

Age-related bone fragility is a major public health problem, and as such, is generally well described. In this study we aimed to characterize the alterations occurring in murine bone due to the ‘normal’ aging process, within the context of alterations in osteoblast metabolic pathways. First, regarding the skeletal alterations occurring *in vivo,* our data demonstrate dramatic loss of trabecular bone, whereas cortical bone increased in terms of both thickness and total cross-sectional area. An explanation of this phenomenon has been previously described as “cortical drift” whereby there is a discrepancy in bone deposition on the periosteal surface, while bone resorption occurs on the endosteal surface, thereby increasing bone diameter during aging [36]. Although cortical drift described in humans is often associated with increase in total cross-sectional area but decrease in cortical thickness and density and increase in porosity [37]. Regardless of this skeletal limitation, osteoblast activity is reduced in aged mice as well as humans [38, 39], therefore further studying these cells throughout the aging process is reasonable. Peak bone mass occurs during equivalent phases of life in human (30-40 years) and C57BL/6J mice (4-6 months; 50% of total life expectancy) [40, 41]. However, this is compartment dependent. For example, trabecular bone attains peak mass as early as 1.5 months in C57BL/6J mice [40], whereas cortical bone takes more time to reach the peak bone mass (around 5/6 months) which can further explain the discrepancy between these bone compartments. Nonetheless, the current study utilized mice at 2- and 22-months of age to ultimately isolate osteoblasts with distinctly different activity with respect to bone formation.

The main goal of the study was to rigorously characterize metabolic profiles between aged (22 mo) and young (2 mo) mice, as these alterations can contribute to age-related decline in bone formation leading to osteoporosis. Previous studies have already shown that metabolic alterations, at both systemic as well as cellular level, contribute to various age-related pathologies including neurodegeneration, atherosclerosis, type 2 diabetes, other cardiovascular diseases [42-45]. Similarly, the current study demonstrates that BMSCs isolated from young mice show distinctly different metabolic profiles compared to BMSCs from old mice. For example, BMSCs isolated from both young and old mice utilize endogenous nutrients to shift their main mode of energy generation from glycolysis to oxidative phosphorylation during osteoblast differentiation and maturation. However, stromal cells and early osteoblast progenitors from old mice are more dependent on oxidative phosphorylation compared to young mice. Although the cells from the young mice can ‘rebound’ their oxidation capacity in mature osteoblasts. Interestingly, contrary to our results, previous studies have reported an increase in ATP produced via glycolysis, during osteoblast maturation [46, 47]. However, unlike these previous studies, we have compared the different modes of metabolism in our study in the absence of any external nutrients, forcing the cells to rely on endogenous substrates for ATP generation. The importance of glycolysis during earlier phases of differentiation in young mice was further confirmed by their higher glucose dependency. We further showed differential preference of nutrients between the two groups throughout the osteoblastogenesis. Mature osteoblasts from aged mice showed more flexibility towards all three nutrients glucose, glutamine, and oleic acid. Additionally, we corroborated the metabolic data from the *ex vivo* differentiating osteoblasts with *in vivo* gene expression profiling of bone. As such, the first-rate limiting enzyme of glycolysis hexokinase (*Hk2*) was upregulated in aged bones while the last rate limiting enzyme of this pathway, pyruvate kinase (*Pklr*), was downregulated indicating although the glucose is being converted to its metabolically active form of glucose-6-phosphate it is being utilized for some pathway other than glycolysis. The upregulation of phosphoglucomutase (*Pgm5*) and the rate limiting enzymes of glycogen synthesis, glycogen synthase 1 and 2 (*Gys1* and *Gys2*) confirmed utilization of glucose more towards glycogen storage than energy generation. Further confirmation of this shift of glucose flux towards the anabolic arm in aged mice came from upregulation of 3 genes belonging to gluconeogenesis pathway (*Fbp2, Ogdhl, Eno4*) including the rate limiting enzyme fructose-1,6-bisphophatase (*Fbp2*). Other than providing glucose, gluconeogenesis also acts as anaplerotic pathway as it produces TCA cycle intermediate oxaloacetate which is a substrate for oxidative phosphorylation mediated ATP generation. Thus, higher gluconeogenesis can lead to the higher oxidative phosphorylation by providing its substrate in aged mice. Oxoglutaratedehydrogenase, encoded by the *Ogdhl* gene, also contributes to oxidative phosphorylation mediated ATP generation by converting oxoglutarate coming from oxidation of glutamate to succinyl-CoA, another TCA cycle intermediate. Availability of TCA cycle intermediates from both glucose and glutamate may explain the metabolic flexibility of matured osteoblasts towards these two nutrients.

Outside of glucose and glutamine, oxidative phosphorylation can generate maximum number of ATPs by using fatty acid as fuel (oxidation of 18-carbon length oleic acid generates 120 ATP). The presence of higher number of lipid droplets in the aged mice during both early and late stage of osteoblastogenesis indicated differential availability of endogenous nutrients. However, in younger mice only matured osteoblasts had considerable number of lipid droplets, which we have previously reported [48]. Measurement of lipolysis by pulse chase assay showed while this process remained constant throughout differentiation in cells from aged mice, in young mice it increased with progression of osteoblast maturation indicating both stromal cells and matured osteoblasts of aged mice are more prone towards utilizing lipid as a source of energy. Moreover, the presence of higher lipid content was confirmed *in vivo* in the marrow devoid long bones of the older mice. While this was an attempt to enrich osteoblastic cells, the bone shaft is also a source of terminally differentiated osteocytes, therefore we cannot delineate which cell population was the primary contributor of bone lipid accumulation. Although, previous reports have demonstrated osteocytes can accumulate lipid droplets [49] [50, 51], our *in vitro* data provides a basis for how aging contributes to this process in osteoblasts. We further confirmed the presence of triglyceride, cholesteryl ester and fatty acid in human bone cortex as well which showed variation between individuals in terms of quantity. To make an age wise comparison between lipid components in human we need much higher sample size to attain statistical significance because in human different lifestyle, food habits can cause more variation compared to our mice model in contained environment. Also, as these human samples are collected from cadaver autopsies, the time of collection may lead to variation. Lesser availability of cadaver samples from younger age group is another technical limitation to do age wise comparison of lipid metabolism in bones in human.

Interestingly, following the trend of our metabolic assays the most affected metabolic pathway in terms of differential gene expression with age was also that of lipid metabolism. In fact, two of the most highly expressed genes in bones of aged mice, *Apol11a* and *Fgfbp3* are involved with lipid metabolism. *Apol11a* codes for a lipoprotein associated with lipid transfer whereas *Fgfbp3* has been shown to be involved with regulating both carbohydrate and lipid metabolism [52]. Other than *Apol11a*, expression of apolipoproteins *Apol10a, Apol10b, Apol6,* and *Apobr* were also upregulated in long bones of aged mice. Lipoprotein lipase (*Lpl*) which releases fatty acid from lipoproteins for cellular uptake was also expressed more in aged mice. Elevated expression of the apolipoproteins along with *Lpl* in aged mice bone indicated higher fatty acid uptake was the main reason for their higher lipid availability. Additional lipid-related genes up-regulated in aged bones are directly involved in β-oxidation pathway (*Acadm, Acsbg1, Acsm5, Mlycd*). Finally, perilipin 5 (*Plin5)* which has been shown to increase association of lipid droplets with mitochondria in highly oxidative tissues like cardiac and skeletal muscles [53] was upregulated in aged bones. Collectively, these data indicate higher lipid flux in bones of aged mice compared to young mice where higher apolipoprotein mediated uptake of fatty acids can lead to higher availability of substrates for both oxidative phosphorylation mediated ATP generation and for generation of lipid droplets.

Although osteoblasts from older mice were able to generate more ATP utilizing endogenous nutrients, unlike young mice they failed to utilize externally added nutrients to increase their ATP production. In fact, it led to higher proton leak in these cells indicating they were not able to adapt to change in external nutrient availability like the younger mice. These data indicated that although cells from aged mice may have higher intracellular nutrient availability, with aging both stromal cells and matured osteoblasts became less adaptive to external environment and more prone towards proton leak. Proton leak can be caused by dysfunctional mitochondria. Interestingly, aging has been proposed to lead to mitochondrial dysfunction in several tissues including bone [54], brain [55], muscle [56] and cardiovascular tissue [57]. Additive effects of increased expression of genes involved in fatty acid uptake and mitochondrial dysfunction can be the reason for the inability of these cells from aged mice to utilize exogenous nutrients. Instead of generating more ATP, the oxidation of excess exogenous fatty acid taken up by these cells was leading to more protons leak due to the presence of damaged mitochondria. Proton leak is also very closely associated with mitochondrial ROS generation and cell death [58]. Several previous reports have also suggested a strong correlation between senescence and oxidative stress [59, 60]. Corroborating with this our RNA sequencing data from bone demonstrated higher expression of oxidative stress marker genes and lower expression of oxidative stress mitigation genes in 22-month-old mice. The Mitochondrial dysfunction can also be one of the reasons for the shift of the lipid flux more towards anabolic lipid droplet formation where the higher availability of intracellular fatty acid due to increased uptake was not being used up completely but was rather leading to accumulation of lipid droplets. Interestingly, we found increased expression of multiple aldehyde dehydrogenases (*Aldh1l1, Aldh1a2, Aldh3b2, Aldh5a1*). These enzymes have been reported to get upregulated under oxidative stress [61, 62], as other than catalyzing their substrate specific reactions, all of them can act as “aldehyde scavenger’ during lipid peroxidation [61]. Oxidative stress can lead to peroxidation of lipid which can result in toxicity and cell death [63, 64]. These aldehyde dehydrogenases are NAD(P)^+^ dependent enzymes that can convert the aldehydes formed due to peroxidation of fatty acids back to their respective carboxylic acids. Recent studies have shown increased lipid droplet production is another defense mechanism used by cells to protect themselves from lipid peroxidation [65, 66]. This way they can hide the unsaturated fatty acids which are more vulnerable to ROS within the core of lipid droplets, esterified in form of neutral lipid like triglyceride or cholesterol ester [65]. Thus, accumulation of lipid droplets in the bone cells of aged mice and upregulation of diacylglycerolacyl-transferase (*Dgat2l6*) the rate limiting enzyme in triglyceride synthesis in bone of aged mice can be a protective measure adapted by these cells against the higher oxidative stress and presence of dysfunctional mitochondria.

In conclusion, our data demonstrate regulated metabolism is important for maintenance of bone homeostasis. Dysregulation of this process due to aging leads to generation of oxidative stress which can further lead to impaired functioning and even death of osteoblasts (Fig. 8) which can be one of the reason for decrease in their number with aging [67]. The overall effect of this is reflected in loss of bone which is one of the leading reasons for age related osteoporosis. Although, accumulation of lipid droplets can be a protective mechanism to adapt to the higher oxidative stress, shifting metabolic energy flux from glycolytic to oxidative phosphorylation to utilize this stored lipid can be the reason behind the higher ROS generation of these cells. Since osteoblasts *in vivo* are exposed to constant external nutrient availability, combinatorial effect of excess uptake of lipid and damaged mitochondria can make them more prone to proton leak and ROS generation. There are multiple previous reports associating increased marrow adiposity with aging in both human and mice [68-70], here we have shown for the first time the lipid metabolism of the bone per se also gets affected due to aging which may further lead to age related bone loss. However, studying site specific metabolic profile and gene expression in cortical versus trabecular bone with aging will be interesting as that might help us to find protective measures against age related osteoporosis.

## Supplementary Materials

The Supplementary data can be found online at: www.aginganddisease.org/EN/10.14336/AD.2023.0510.



## References

[1] Weilbaecher KN, Guise TA, McCauley LK (2011). Cancer to bone: a fatal attraction. Nat Rev Cancer, 11:411-425. 21593787 10.1038/nrc3055 PMC3666847

[2] Zaidi M (2007). Skeletal remodeling in health and disease. Nat Med, 13:791-801. 17618270 10.1038/nm1593

[3] Iqbal J, Sun L, Zaidi M (2009). Coupling bone degradation to formation. Nat Med, 15:729-731. 19584858 10.1038/nm0709-729

[4] Etich J, Lessmeier L, Rehberg M, Sill H, Zaucke F, Netzer C, et al. (2020). Osteogenesis imperfecta-pathophysiology and therapeutic options. Mol Cell Pediatr, 7:9. 32797291 10.1186/s40348-020-00101-9 PMC7427672

[5] Garibaldi N, Contento BM, Babini G, Morini J, Siciliani S, Biggiogera M, et al. (2021). Targeting cellular stress in vitro improves osteoblast homeostasis, matrix collagen content and mineralization in two murine models of osteogenesis imperfecta. Matrix Biol, 98:1-20. 33798677 10.1016/j.matbio.2021.03.001 PMC11162743

[6] Al-Bari AA, Al Mamun A (2020). Current advances in regulation of bone homeostasis. FASEB Bioadv, 2:668-679. 33205007 10.1096/fba.2020-00058 PMC7655096

[7] Dhanwal DK (2011). Thyroid disorders and bone mineral metabolism. Indian J Endocrinol Metab, 15:S107-112. 21966645 10.4103/2230-8210.83339 PMC3169869

[8] Emmanuelle NE, Marie-Cecile V, Florence T, Jean-Francois A, Francoise L, Coralie F, et al. (2021). Critical Role of Estrogens on Bone Homeostasis in Both Male and Female: From Physiology to Medical Implications. Int J Mol Sci, 22. 33557249 10.3390/ijms22041568 PMC7913980

[9] Jin J, Wang Y, Jiang H, Kourkoumelis N, Renaudineau Y, Deng Z (2018). The impact of obesity through fat depots and adipokines on bone homeostasis. AME Medical Journal, 3:10-10.

[10] Chandra A, Rajawat J (2021). Skeletal Aging and Osteoporosis: Mechanisms and Therapeutics. Int J Mol Sci, 22. 33805567 10.3390/ijms22073553 PMC8037620

[11] Halloran BP, Ferguson VL, Simske SJ, Burghardt A, Venton LL, Majumdar S (2002). Changes in Bone Structure and Mass with Advancing Age in the Male C57BL 6J Mouse. J Bone Miner Res, 17:1044-1050. 12054159 10.1359/jbmr.2002.17.6.1044

[12] Shim J, Iwaya C, Ambrose CG, Suzuki A, Iwata J (2022). Micro-computed tomography assessment of bone structure in aging mice. Sci Rep, 12:8117. 35581227 10.1038/s41598-022-11965-4 PMC9114112

[13] Lai P, Song Q, Yang C, Li Z, Liu S, Liu B, et al. (2016). Loss of Rictor with aging in osteoblasts promotes age-related bone loss. Cell Death Dis, 7:e2408. 27735936 10.1038/cddis.2016.249 PMC5133960

[14] Almeida M (2012). Aging mechanisms in bone. Bonekey Rep, 1. 23705067 10.1038/bonekey.2012.102 PMC3659822

[15] Almeida M, Han L, Martin-Millan M, O'Brien CA, Manolagas SC (2007). Oxidative stress antagonizes Wnt signaling in osteoblast precursors by diverting beta-catenin from T cell factor- to forkhead box O-mediated transcription. J Biol Chem, 282:27298-27305. 17623658 10.1074/jbc.M702811200

[16] Tiede-Lewis LM, Xie Y, Hulbert MA, Campos R, Dallas MR, Dusevich V, et al. (2017). Degeneration of the osteocyte network in the C57BL/6 mouse model of aging. Aging (Albany NY), 9:2190-2208. 29074822 10.18632/aging.101308 PMC5680562

[17] Gerdhem P, Isaksson A, Akesson K, Obrant KJ (2005). Increased bone density and decreased bone turnover, but no evident alteration of fracture susceptibility in elderly women with diabetes mellitus. Osteoporos Int, 16:1506-1512. 15824889 10.1007/s00198-005-1877-5

[18] Duque G (2008). Bone and fat connection in aging bone. Curr Opin Rheumatol, 20:429-434. 18525356 10.1097/BOR.0b013e3283025e9c

[19] Moerman EJ, Teng K, Lipschitz DA, Lecka-Czernik B (2004). Aging activates adipogenic and suppresses osteogenic programs in mesenchymal marrow stroma/stem cells: the role of PPAR-gamma2 transcription factor and TGF-beta/BMP signaling pathways. Aging Cell, 3:379-389. 15569355 10.1111/j.1474-9728.2004.00127.x PMC1850101

[20] Jilka RL, Weinstein RS, Parfitt AM, Manolagas SC (2007). Quantifying osteoblast and osteocyte apoptosis: challenges and rewards. J Bone Miner Res, 22:1492-1501. 17542686 10.1359/jbmr.070518

[21] Nojiri H, Saita Y, Morikawa D, Kobayashi K, Tsuda C, Miyazaki T, et al. (2011). Cytoplasmic superoxide causes bone fragility owing to low-turnover osteoporosis and impaired collagen cross-linking. J Bone Miner Res, 26:2682-2694. 22025246 10.1002/jbmr.489

[22] Kuk JL, Saunders TJ, Davidson LE, Ross R (2009). Age-related changes in total and regional fat distribution. Ageing Res Rev, 8:339-348. 19576300 10.1016/j.arr.2009.06.001

[23] Roger H Unger LO (2002). <pancreas.pdf>. Biochimica et Biophysica Acta (BBA) - Molecular and Cell Biology of Lipids, 1585:202-212.12531555

[24] Slawik M, Vidal-Puig AJ (2006). Lipotoxicity, overnutrition and energy metabolism in aging. Ageing Res Rev, 5:144-164. 16630750 10.1016/j.arr.2006.03.004

[25] Zhou Y-T, Grayburn P, Karim A, Shimabukuro M, Higa M, Baetens D, et al. (2000). Lipotoxic heart disease in obese rats: implications for human obesity. Proceedings of the National Academy of Sciences, 97:1784-1789. 10.1073/pnas.97.4.1784 PMC26513 10677535

[26] Duque G (2007). As a matter of fat: New perspectives on the understanding of age-related bone loss. BoneKEy-Osteovision, 4:129-140.

[27] Duque G (2008). Bone and fat connection in aging bone. Current Opinion in Rheumatology, 20:429-434. 18525356 10.1097/BOR.0b013e3283025e9c

[28] Justesen J, Stenderup K, Ebbesen EN, Mosekilde L, Steiniche T, Kassem M (2001). Adipocyte tissue volume in bone marrow is increased with aging and in patients with osteoporosis. Biogerontology, 2:165-171. 11708718 10.1023/a:1011513223894

[29] Beata Lecka-Czernik EJM, David FG, Jürgen ML, Stavros CM, Robert LJ ( 2002). Divergent Effects of Selective Peroxisome Proliferator-Activated Receptor-γ2 Ligands on Adipocyte Versus Osteoblast Differentiation. Endocrinology, 143:2376-2384. 12021203 10.1210/endo.143.6.8834

[30] Maridas DE, Rendina-Ruedy E, Le PT, Rosen CJ (2018). Isolation, Culture, and Differentiation of Bone Marrow Stromal Cells and Osteoclast Progenitors from Mice. [J] Vis Exp. 10.3791/56750 PMC5908449 29364278

[31] Phinney DG KG, Isaacson RL, Prockop DJ (1999). Plastic adherent stromal cells from the bone marrow of commonly used strains of inbred mice: variations in yield, growth, and differentiation. J Cell Biochem, 72:570-585. 10022616

[32] Sun S GZ, Xiao X, Liu B, Liu X, Tang PH, Mao N (2003). Isolation of mouse marrow mesenchymal progenitors by a novel and reliable method. Stem Cells, 21:527-535. 12968107 10.1634/stemcells.21-5-527

[33] Jayapalan S, Nandy A, Rendina-Ruedy E (2022). Using Real-Time Cell Metabolic Flux Analyzer to Monitor Osteoblast Bioenergetics. [J] Vis Exp. 10.3791/63142 PMC10962472 35311813

[34] Bligh EG, Dyer WJ (1959). A rapid method of total lipid extraction and purification. Canadian journal of biochemistry and physiology, 37:911-917. 13671378 10.1139/o59-099

[35] Rambold AS, Cohen S, Lippincott-Schwartz J (2015). Fatty acid trafficking in starved cells: regulation by lipid droplet lipolysis, autophagy, and mitochondrial fusion dynamics. Dev Cell, 32:678-692. 25752962 10.1016/j.devcel.2015.01.029 PMC4375018

[36] Goldman HM, McFarlin SC, Cooper DM, Thomas CD, Clement JG (2009). Ontogenetic patterning of cortical bone microstructure and geometry at the human mid-shaft femur. Anat Rec (Hoboken), 292:48-64. 19051245 10.1002/ar.20778

[37] Garn SM, Sullivan TV, Decker SA, Larkin FA, Hawthorne VM (1992). Continuing bone expansion and increasing bone loss over a two-decade period in men and women from a total community sample. Am J Hum Biol, 4:57-67. 28524405 10.1002/ajhb.1310040109

[38] Tiede-Lewis LM, Xie Y, Hulbert MA, Campos R, Dallas MR, Dusevich V, et al. (2017). Degeneration of the osteocyte network in the C57BL/6 mouse model of aging. Aging, 9(10):2190-2208 29074822 10.18632/aging.101308 PMC5680562

[39] Becerikli M, Jaurich H, Schira J, Schulte M, Dobele C, Wallner C, et al. (2017). Age-dependent alterations in osteoblast and osteoclast activity in human cancellous bone. J Cell Mol Med, 21:2773-2781. 28444839 10.1111/jcmm.13192 PMC5661248

[40] Halloran BP, Ferguson VL, Simske SJ, Burghardt A, Venton LL, Majumdar S (2002). Changes in bone structure and mass with advancing age in the male C57BL/6J mouse. J Bone Miner Res, 17:1044-1050. 12054159 10.1359/jbmr.2002.17.6.1044

[41] Bar-Shira-Maymon B, Coleman R, Cohen A, Steinhagen-Thiessen E, Silbermann M (1989). Age-related bone loss in lumbar vertebrae of CW-1 female mice: a histomorphometric study. Calcif Tissue Int, 44:36-45. 2492885 10.1007/BF02556238

[42] Wesley UV, Bhute VJ, Hatcher JF, Palecek SP, Dempsey RJ (2019). Local and systemic metabolic alterations in brain, plasma, and liver of rats in response to aging and ischemic stroke, as detected by nuclear magnetic resonance (NMR) spectroscopy. Neurochem Int, 127:113-124. 30707914 10.1016/j.neuint.2019.01.025

[43] Lettieri-Barbato D, Ventura N, Faraonio R, Aquilano K (2020). Editorial: Advances in Metabolic Mechanisms of Aging and Its Related Diseases. Front Physiol, 11:594974. 33132919 10.3389/fphys.2020.594974 PMC7579133

[44] Qi G, Mi Y, Yin F (2019). Cellular Specificity and Inter-cellular Coordination in the Brain Bioenergetic System: Implications for Aging and Neurodegeneration. Front Physiol, 10:1531. 31969828 10.3389/fphys.2019.01531 PMC6960098

[45] Sabbatinelli J, Prattichizzo F, Olivieri F, Procopio AD, Rippo MR, Giuliani A (2019). Where Metabolism Meets Senescence: Focus on Endothelial Cells. Front Physiol, 10:1523. 31920721 10.3389/fphys.2019.01523 PMC6930181

[46] Guntur AR, Le PT, Farber CR, Rosen CJ (2014). Bioenergetics during calvarial osteoblast differentiation reflect strain differences in bone mass. Endocrinology, 155:1589-1595. 24437492 10.1210/en.2013-1974 PMC3990840

[47] Misra BB, Jayapalan S, Richards AK, Helderman RCM, Rendina-Ruedy E (2021). Untargeted metabolomics in primary murine bone marrow stromal cells reveals distinct profile throughout osteoblast differentiation. Metabolomics, 17:86. 34537901 10.1007/s11306-021-01829-9 PMC8450216

[48] Rendina-Ruedy E, Guntur AR, Rosen CJ (2017). Intracellular lipid droplets support osteoblast function. Adipocyte, 6:250-258. 28792783 10.1080/21623945.2017.1356505 PMC5638385

[49] Kawai K, Tamaki A, Hirohata K (1985). Steroid-induced accumulation of lipid in the osteocytes of the rabbit femoral head. A histochemical and electron microscopic study. JBJS, 67:755-763. 3997928

[50] Wang Y, Li Y, Mao K, Li J, Cui Q, Wang G-J (2003). Alcohol-Induced Adipogenesis in Bone and Marrow: A Possible Mechanism for Osteonecrosis. Clinical Orthopaedics and Related Research®, 410:213-224. 10.1097/01.blo.0000063602.67412.83 12771833

[51] Maurel DB, Pallu S, Jaffre C, Fazzalari NL, Boisseau N, Uzbekov R, et al. (2012). Osteocyte apoptosis and lipid infiltration as mechanisms of alcohol-induced bone loss. Alcohol Alcohol, 47:413-422. 22596044 10.1093/alcalc/ags057

[52] Tassi E, Garman KA, Schmidt MO, Ma X, Kabbara KW, Uren A, et al. (2018). Fibroblast Growth Factor Binding Protein 3 (FGFBP3) impacts carbohydrate and lipid metabolism. Sci Rep, 8:15973. 30374109 10.1038/s41598-018-34238-5 PMC6206164

[53] Kimmel AR, Sztalryd C (2014). Perilipin 5, a lipid droplet protein adapted to mitochondrial energy utilization. Curr Opin Lipidol, 25:110-117. 24535284 10.1097/MOL.0000000000000057 PMC4517968

[54] Shum LC, White NS, Nadtochiy SM, Bentley KL, Brookes PS, Jonason JH, et al. (2016). Cyclophilin D Knock-Out Mice Show Enhanced Resistance to Osteoporosis and to Metabolic Changes Observed in Aging Bone. PLoS One, 11:e0155709. 27183225 10.1371/journal.pone.0155709 PMC4868300

[55] Eliseev RA, Filippov G, Velos J, VanWinkle B, Goldman A, Rosier RN, et al. (2007). Role of cyclophilin D in the resistance of brain mitochondria to the permeability transition. Neurobiol Aging, 28:1532-1542. 16876914 10.1016/j.neurobiolaging.2006.06.022

[56] Figueiredo PA, Powers SK, Ferreira RM, Appell HJ, Duarte JA (2009). Aging impairs skeletal muscle mitochondrial bioenergetic function. J Gerontol A Biol Sci Med Sci, 64:21-33. 19196905 10.1093/gerona/gln048 PMC2691197

[57] Harrison CM, Pompilius M, Pinkerton KE, Ballinger SW (2011). Mitochondrial oxidative stress significantly influences atherogenic risk and cytokine-induced oxidant production. Environ Health Perspect, 119:676-681. 21169125 10.1289/ehp.1002857 PMC3094420

[58] Nanayakkara GK, Wang H, Yang X (2019). Proton leak regulates mitochondrial reactive oxygen species generation in endothelial cell activation and inflammation - A novel concept. Arch Biochem Biophys, 662:68-74. 30521782 10.1016/j.abb.2018.12.002 PMC6800189

[59] Liguori I, Russo G, Curcio F, Bulli G, Aran L, Della-Morte D, et al. (2018). Oxidative stress, aging, and diseases. Clin Interv Aging, 13:757-772. 29731617 10.2147/CIA.S158513 PMC5927356

[60] Stefanatos R, Sanz A (2018). The role of mitochondrial ROS in the aging brain. FEBS Lett, 592:743-758. 29106705 10.1002/1873-3468.12902

[61] Singh S, Brocker C, Koppaka V, Chen Y, Jackson BC, Matsumoto A, et al. (2013). Aldehyde dehydrogenases in cellular responses to oxidative/electrophilic stress. Free Radic Biol Med, 56:89-101. 23195683 10.1016/j.freeradbiomed.2012.11.010 PMC3631350

[62] Chen Y, Mehta G, Vasiliou V (2009). Antioxidant defenses in the ocular surface. Ocul Surf, 7:176-185. 19948101 10.1016/s1542-0124(12)70185-4 PMC4104792

[63] Brooks PJ, Theruvathu JA (2005). DNA adducts from acetaldehyde: implications for alcohol-related carcinogenesis. Alcohol, 35:187-193. 16054980 10.1016/j.alcohol.2005.03.009

[64] Jacobs AT, Marnett LJ (2010). Systems Analysis of Protein Modification and Cellular Responses Induced by Electrophile Stress. Accounts of Chemical Research, 43:673-683. 20218676 10.1021/ar900286y PMC2873822

[65] Bailey AP, Koster G, Guillermier C, Hirst EM, MacRae JI, Lechene CP, et al. (2015). Antioxidant Role for Lipid Droplets in a Stem Cell Niche of Drosophila. Cell, 163:340-353. 26451484 10.1016/j.cell.2015.09.020 PMC4601084

[66] Lee J, Homma T, Kurahashi T, Kang ES, Fujii J (2015). Oxidative stress triggers lipid droplet accumulation in primary cultured hepatocytes by activating fatty acid synthesis. Biochem Biophys Res Commun, 464:229-235. 26116535 10.1016/j.bbrc.2015.06.121

[67] LeAnn M. Tiede-Lewis YX, Hulbert Molly A (2017). Degeneration of the osteocyte network in the C57BL/6 mouse model of aging. Aging, 9. 10.18632/aging.101308 PMC5680562 29074822

[68] Griffith JF, Yeung DK, Ma HT, Leung JC, Kwok TC, Leung PC (2012). Bone marrow fat content in the elderly: a reversal of sex difference seen in younger subjects. J Magn Reson Imaging, 36:225-230. 22337076 10.1002/jmri.23619

[69] Tuljapurkar SR, McGuire TR, Brusnahan SK, Jackson JD, Garvin KL, Kessinger MA, et al. (2011). Changes in human bone marrow fat content associated with changes in hematopoietic stem cell numbers and cytokine levels with aging. J Anat, 219:574-581. 21923862 10.1111/j.1469-7580.2011.01423.x PMC3191309

[70] Ambrosi TH, Scialdone A, Graja A, Gohlke S, Jank AM, Bocian C, et al. (2017). Adipocyte Accumulation in the Bone Marrow during Obesity and Aging Impairs Stem Cell-Based Hematopoietic and Bone Regeneration. Cell Stem Cell, 20:771-784 e776. 28330582 10.1016/j.stem.2017.02.009 PMC5459794

