# Supplementary material for: Altered Osteoblast Metabolism with Aging Results in Lipid Accumulation and Oxidative Stress Mediated Bone Loss
Source: Aging Dis. 2024 Apr 1;15(2):767–86. doi: 10.14336/AD.2023.0510 (PMC10917552; doi:10.14336/AD.2023.0510)
Supplement: Supplementary file 1 [file AD-15-2-767-s.pdf]

SUPPLEMENTARY DATA

Altered Osteoblast Metabolism with Aging Results in Lipid Accumulation and Oxidative Stress Mediated Bone Loss

Ananya Nandy, Alison Richards, Santosh Thapa, Alena Akhmetshina[1,3], Nikita Narayani, Elizabeth Rendina-Ruedy

© 2023. Nandy A et al. Published online at http://www.aginganddisease.org/EN/10.14336/AD.2023.0510

SUPPLEMENTARY DATA

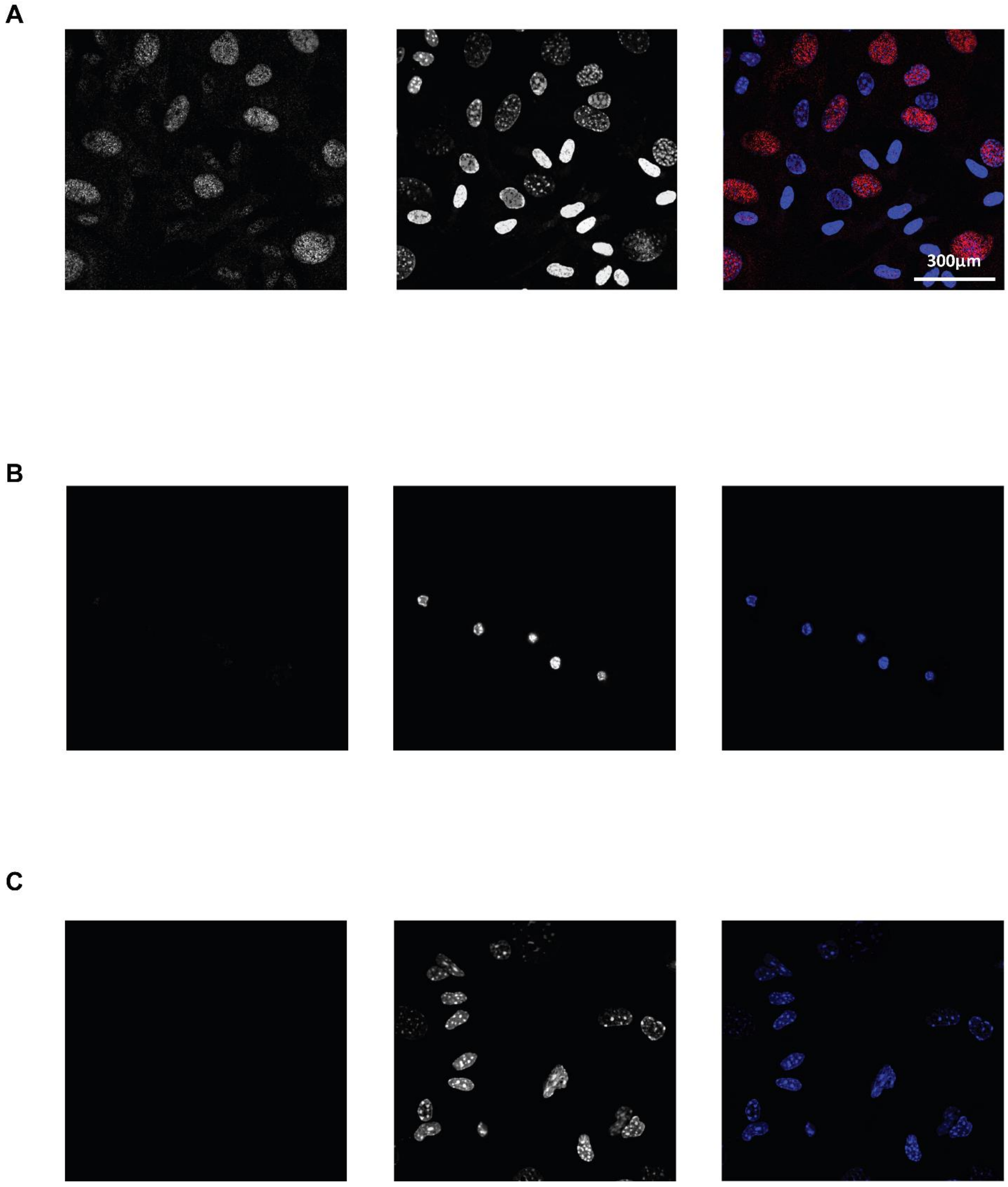

Supplementary Figure 1. Confirmation of presence of stromal osteogenic population in BMSC. Representative confocal image of (A) undifferentiated stromal cells (B) hematopoietic stem cells from 2 months old mice immunostained for Runx2 with DAPI staining. (C) Representative confocal image of secondary antibody control of undifferentiated stromal cells. Panel 1 shows monochrome images of nuclei staining by Runx2 whereas panel 2 shows monochrome images of nuclei staining by DAPI and panel 3 shows merged image (Runx2 in red and DAPI in blue).

© 2023. Nandy A et al. Published online at http://www.aginganddisease.org/EN/10.14336/AD.2023.0510

SUPPLEMENTARY DATA

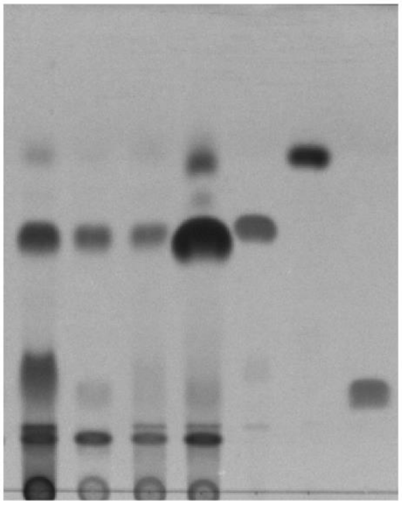

Supplementary Figure 2. Neutral lipid profile in human bones. (A) Thin layer chromatogram of lipid harvested from flushed femur (femur without any bone marrow) from human autopsy samples (between 65–82-year-old), where N=4.

Supplementary Table 1. RNA-Seq Library Characteristics

Sample	Input reads	Uniquely mapped reads	Mapped to multiple loci	Mapped to too many loci	Uniquely mapped ratio	Mapped ratio
22 months_S1	88978230	60991460	19072707	145724	0.685465	0.899818
22 months_S2	91114346	70632257	11150180	156501	0.7752046	0.89758
22 months_S3	78176631	48084275	18595342	113976	0.6150722	0.852935
22 months_S4	100089131	69557142	15396525	163066	0.694952	0.84878
22 months_S5	106170598	68429366	20485720	159666	0.6445228	0.837474
2 months_S1	82257395	60355141	13020984	135690	0.7337351	0.892031
2 months_S2	83851103	59959894	18078416	169554	0.7150758	0.930677
2 months_S3	83550485	50970338	23417229	151004	0.6100544	0.890331
2 months_S4	88662528	70689173	11016474	138377	0.7972835	0.921535
2 months_S5	84054937	64954566	10713962	212766	0.7727632	0.900227

© 2023. Nandy A et al. Published online at http://www.aginganddisease.org/EN/10.14336/AD.2023.0510

SUPPLEMENTARY DATA

Supplementary Table 2. Differentially expressed genes in bones of aged mice versus young mice

2.1. Statistically significant (FDR adj p≤0.05) upregulated (≥1.5 fold genes)

**Feature_gene_biotype**	**Feature_gene_name**	**padj**	**FoldChange**
protein_coding	Apol11a	1.55E-58	9.06850266
protein_coding	Fgfbp3	8.88E-49	10.0127576
protein_coding	Dock3	2.34E-42	14.0422674
protein_coding	Snhg11	2.34E-42	6.67281076
protein_coding	Rasd2	1.63E-24	8.54679602
protein_coding	Tmem132b	7.01E-15	4.89614222
protein_coding	Foxd1	9.18E-15	4.03363767
protein_coding	Nol4	1.56E-14	5.43152833
protein_coding	Aldh1a2	2.19E-14	5.02955777
protein_coding	Gpnmb	4.13E-14	3.81051395
protein_coding	Kcna4	2.04E-13	9.60451513
protein_coding	Apol11b	1.98E-12	2.38474397
lncRNA	Srrm4os	2.87E-12	4.30415743
protein_coding	C7	9.76E-12	5.07135898
protein_coding	Mlph	4.97E-11	3.44928659
protein_coding	Kif1a	5.40E-11	2.34417792
protein_coding	Kif5c	1.65E-10	2.74002093
protein_coding	Cacna1h	2.78E-10	2.87496665
protein_coding	Slc24a2	6.09E-10	5.17751917
protein_coding	Nr3c2	1.44E-09	2.21286148
IG_C_gene	Ighg3	2.61E-09	17.8444354
unprocessed_pseudogene	Gm7582	6.05E-09	8.33997973
protein_coding	Zfp773	6.11E-09	2.3280553
protein_coding	Trpm2	6.23E-09	1.74024298
protein_coding	Ide	1.72E-08	2.06579628
protein_coding	Lancl3	2.39E-08	2.99131813
protein_coding	Trim29	3.22E-08	12.4982741
protein_coding	Fam241b	4.01E-08	5.24246739
protein_coding	Ppp1r14bl	4.01E-08	4.6027872
lncRNA	Gm14461	4.40E-08	27.7366219
protein_coding	Lhfpl4	5.41E-08	3.26604056
protein_coding	Trp63	5.64E-08	2.98940886
protein_coding	Olfm4	7.79E-08	2.45711144
protein_coding	Plekhb1	8.63E-08	2.21912371
protein_coding	Astn2	9.19E-08	2.98975737
protein_coding	Cd163l1	9.30E-08	7.92614374

© 2023. Nandy A et al. Published online at http://www.aginganddisease.org/EN/10.14336/AD.2023.0510

protein_coding	Shisa3	9.30E-08	2.16649193
protein_coding	Prkcz	1.16E-07	1.9585339
protein_coding	Lax1	1.17E-07	2.02653491
protein_coding	Greb1	1.21E-07	4.2301689
IG_V_gene	Igkv3-4	1.25E-07	23.240476
protein_coding	Apol10a	1.34E-07	10.9882017
IG_C_gene	Igha	1.87E-07	8.5115146
protein_coding	Amy1	2.55E-07	3.29518083
protein_coding	Gm10800	2.69E-07	946.568968
protein_coding	Faah	3.20E-07	2.71335229
protein_coding	Phf24	4.74E-07	2.65632673
protein_coding	Icosl	5.33E-07	1.76017663
protein_coding	Sfxn5	5.36E-07	1.74349725
transcribed_unprocessed_pseudogene	Olfr1372-ps1	5.91E-07	2.95394361
lncRNA	Gm36252	6.68E-07	15.2479285
protein_coding	Rad51ap2	6.87E-07	2.48781117
IG_V_gene	Ighv1-53	7.81E-07	4.39443793
lncRNA	Gm2619	9.45E-07	54.5268663
protein_coding	D430041D05Rik	1.08E-06	3.03511927
protein_coding	Cirbp	1.09E-06	1.72426397
protein_coding	Crtam	1.11E-06	2.51969216
protein_coding	Sh2d3c	1.11E-06	1.63650697
protein_coding	Vwa5b1	1.15E-06	26.4248074
TEC	Gm43181	1.15E-06	2.24518737
protein_coding	Chst1	1.29E-06	2.75889293
protein_coding	Anxa3	1.32E-06	1.53148346
protein_coding	Abcb1b	1.35E-06	1.64545966
protein_coding	Mib1	1.53E-06	1.65634455
protein_coding	Chd7	1.53E-06	1.6526047
protein_coding	Tox2	1.55E-06	2.70323866
protein_coding	Mapk11	1.71E-06	1.78610986
protein_coding	F2rl1	2.08E-06	3.43621242
lncRNA	Gm47015	2.08E-06	3.21015111
protein_coding	Cpeb2	2.08E-06	1.65435585
transcribed_unprocessed_pseudogene	Gm10499	2.51E-06	5.34548584
protein_coding	Hcn1	2.74E-06	4.46638097
protein_coding	Syp	2.97E-06	2.49099986
protein_coding	Gm19410	3.10E-06	7.10987651
protein_coding	Nlrp12	3.17E-06	1.58337781
protein_coding	Jchain	3.18E-06	7.24258384
protein_coding	Mill2	3.59E-06	1.62174038

© 2023. Nandy A et al. Published online at http://www.aginganddisease.org/EN/10.14336/AD.2023.0510

protein_coding	Nrxn2	3.74E-06	2.71282153
IG_V_gene	Ighv2-3	3.83E-06	11.1994891
protein_coding	Myh14	4.02E-06	3.40341155
lncRNA	3222401L13Rik	4.09E-06	2.05687797
protein_coding	Cbx7	4.21E-06	1.56746254
IG_V_gene	Ighv11-2	4.54E-06	5.10929757
protein_coding	Foxd4	4.56E-06	2.00728561
protein_coding	Dmxl2	4.57E-06	1.54381063
protein_coding	Elf3	4.59E-06	6.21833119
protein_coding	Gabre	4.64E-06	2.23326151
IG_V_gene	Igkv6-32	4.74E-06	5.83271711
protein_coding	Eml6	4.94E-06	1.75848432
protein_coding	Gdf7	6.01E-06	2.3753529
protein_coding	Aatk	6.21E-06	1.74741598
IG_C_gene	Ighg2b	6.26E-06	16.4070371
protein_coding	Apol6	6.38E-06	2.29419149
protein_coding	Gm10801	6.41E-06	72.4820481
protein_coding	Ccdc136	7.48E-06	2.1083831
protein_coding	Plekha7	7.57E-06	1.94790048
protein_coding	Ces1d	7.87E-06	2.72817934
protein_coding	Prmt8	9.16E-06	4.72000569
protein_coding	C130026I21Rik	9.35E-06	4.03928688
protein_coding	Fut1	9.84E-06	31.7876992
protein_coding	Tdrkh	1.19E-05	1.85103329
protein_coding	Gdf15	1.22E-05	3.59976731
protein_coding	B4galnt4	1.28E-05	2.69366391
protein_coding	Pdcd1lg2	1.34E-05	3.75556061
protein_coding	Slc6a17	1.34E-05	2.65704522
protein_coding	Spag6	1.46E-05	2.99854152
protein_coding	Tdrd9	1.54E-05	4.42044713
protein_coding	Pik3c2g	1.62E-05	3.27644798
protein_coding	Pdzd3	1.78E-05	2.31540728
protein_coding	Hrh2	1.81E-05	1.69725189
protein_coding	Bcl2	1.81E-05	1.52981584
protein_coding	Myh3	1.85E-05	9.84126724
protein_coding	Hook1	1.95E-05	1.94799199
protein_coding	Clec2f	2.13E-05	3.16556872
protein_coding	Sgip1	2.18E-05	1.92950419
protein_coding	Gys2	2.20E-05	7.06709839
protein_coding	Crtac1	2.20E-05	1.97541928
protein_coding	Eda2r	2.25E-05	2.48739763

© 2023. Nandy A et al. Published online at http://www.aginganddisease.org/EN/10.14336/AD.2023.0510

SUPPLEMENTARY DATA

lncRNA	Crnde	2.32E-05	1.60985371
protein_coding	Ccdc181	2.32E-05	1.57029202
protein_coding	H2-Q10	2.48E-05	1.60569322
processed_pseudogene	Gm50243	2.97E-05	3.68932748
protein_coding	Gimap4	3.01E-05	1.94242981
protein_coding	Cpne7	3.04E-05	2.51536257
transcribed_unprocessed_pseudogene	Gm16026	3.10E-05	11.2844254
lncRNA	4930512J16Rik	3.11E-05	4.81350718
unprocessed_pseudogene	Btnl7-ps	3.13E-05	3.20312482
protein_coding	Mvp	3.39E-05	1.51386892
protein_coding	Ankrd1	3.52E-05	5.36914503
protein_coding	Zkscan2	3.54E-05	3.36645829
protein_coding	Fgf9	3.69E-05	2.43762338
protein_coding	Ryr2	3.81E-05	2.94430473
lncRNA	Lncpint	4.11E-05	1.66911879
protein_coding	Ntng2	4.19E-05	1.55211271
protein_coding	Psd2	4.21E-05	2.58749356
TEC	5330406M23Rik	4.50E-05	1.62151612
processed_pseudogene	Gm12240	4.54E-05	9.20526085
protein_coding	Ddn	4.59E-05	2.72239543
protein_coding	L1cam	4.59E-05	1.53184602
IG_V_gene	Igkv4-72	4.79E-05	6.39180466
unprocessed_pseudogene	Gm29284	5.33E-05	58.6151801
protein_coding	Padi4	5.61E-05	1.54946111
protein_coding	Lgals4	5.77E-05	1.52757409
protein_coding	Dennd3	5.97E-05	1.51126886
protein_coding	Nfasc	6.04E-05	2.8376476
transcribed_unprocessed_pseudogene	Apol10c-ps	6.50E-05	2.38682787
protein_coding	Piwil2	6.52E-05	1.75290737
protein_coding	H2-Q2	6.61E-05	42.6965898
protein_coding	Acod1	6.61E-05	3.58883909
protein_coding	Gli1	6.81E-05	1.79372358
IG_V_gene	Igkv6-15	7.21E-05	5.42859105
protein_coding	Aldh3b2	7.94E-05	1.73344583
protein_coding	Gata6	8.02E-05	3.86652198
protein_coding	Esrrb	8.23E-05	3.28661231
protein_coding	Ildr2	8.24E-05	2.09614578
protein_coding	Btnl2	8.24E-05	2.03037386
protein_coding	Podn	8.41E-05	1.97803558
protein_coding	Wnt1	8.78E-05	3.41864372
protein_coding	Dok6	8.86E-05	2.53445369

© 2023. Nandy A et al. Published online at http://www.aginganddisease.org/EN/10.14336/AD.2023.0510

protein_coding	Grin2c	9.23E-05	3.9617058
lncRNA	Gm7967	9.61E-05	2.38125342
protein_coding	Phactr1	9.81E-05	1.87909268
protein_coding	Tap2	9.81E-05	1.64797532
protein_coding	Musk	9.89E-05	2.59837366
protein_coding	Neurl3	9.93E-05	1.51518778
protein_coding	Rhobtb1	0.00010435	1.54816741
IG_V_gene	Igkv5-43	0.00010516	34.5111938
protein_coding	Ldlr	0.00010874	1.52833765
protein_coding	Mapk13	0.00011541	1.63070502
protein_coding	Mapt	0.00011696	2.34776314
lncRNA	Gm44659	0.00012073	2.76283894
protein_coding	Abca8b	0.00012241	2.02128878
protein_coding	Sirpb1c	0.00012619	1.90190028
protein_coding	Cxcl13	0.00012682	7.73002776
protein_coding	Camk2b	0.00012682	2.4563008
protein_coding	Styk1	0.00014051	2.00010446
protein_coding	Col4a3	0.00014201	2.07532474
protein_coding	C4b	0.00014447	1.9999709
protein_coding	Rab11fip5	0.00014638	1.65957333
protein_coding	Nfatc2	0.00014836	1.5934894
protein_coding	Slc26a8	0.00015675	1.74894481
protein_coding	Col7a1	0.00016026	2.74924794
protein_coding	Wnt10b	0.00016256	1.56871993
IG_C_gene	Igkc	0.00016282	6.17972798
protein_coding	Rasl10a	0.00016512	2.06209519
protein_coding	Dnah10	0.00017177	2.21790118
protein_coding	Palm3	0.00017197	2.17033945
protein_coding	C87977	0.00017383	41.7518715
protein_coding	Acer2	0.00017591	1.55082751
lncRNA	9330162B11Rik	0.00017772	6.80309462
protein_coding	Plekha6	0.00017991	2.24949276
protein_coding	Baiap3	0.00018655	2.11887861
protein_coding	Pcdhga1	0.00018713	1.89117611
transcribed_processed_pseudogene	Smarca5-ps	0.00019047	5.38080505
protein_coding	Nwd1	0.00020062	2.11365882
protein_coding	Jhy	0.00020865	3.04553685
protein_coding	Gm36079	0.00021422	2.12362181
protein_coding	Rnf43	0.00021751	1.66348338
protein_coding	Pou3f1	0.00021938	3.62475352
protein_coding	Atp13a5	0.00021938	3.11381646

© 2023. Nandy A et al. Published online at http://www.aginganddisease.org/EN/10.14336/AD.2023.0510

protein_coding	Gimap8	0.00022356	1.644161
protein_coding	Olfr111	0.00023634	7.54489402
unprocessed_pseudogene	Gm15753	0.00024474	3.51410478
transcribed_unprocessed_pseudogene	Klra13-ps	0.0002454	2.2135106
protein_coding	Igf2	0.00024638	1.95691334
protein_coding	AC167036.2	0.00025066	4.37472185
processed_pseudogene	Gm47994	0.00025326	2.04527469
IG_V_gene	Ighv1-39	0.00025745	5.13065205
protein_coding	Pik3r6	0.00026432	1.62065739
protein_coding	Fibcd1	0.00027265	3.48313952
protein_coding	Cdh22	0.0002779	3.63195509
protein_coding	Dennd1c	0.0002881	1.57717844
protein_coding	Nfe2l1	0.00029478	1.98802601
protein_coding	Abca8a	0.00029963	1.78504816
protein_coding	Xdh	0.00030707	1.55825127
protein_coding	Il18r1	0.00030865	1.79885367
lncRNA	AC106834.1	0.00031509	2.69167325
processed_pseudogene	Rpl19-ps1	0.00031689	7.28732028
protein_coding	Card10	0.00031881	1.6500625
protein_coding	Mef2b	0.00032708	3.23422594
protein_coding	Il1b	0.0003276	2.04007829
protein_coding	1700029H14Rik	0.00033967	2.18135676
protein_coding	Sncg	0.00034208	2.115921
protein_coding	Lilr4b	0.00034429	1.6629985
protein_coding	Pou6f1	0.00034597	1.54362497
lncRNA	Gm10863	0.00035158	2.07707452
protein_coding	Myh8	0.00035402	2.12389578
protein_coding	Efhd1	0.00035583	2.2851516
protein_coding	Trem2	0.00035583	2.06112284
protein_coding	Pak6	0.00036236	3.76006136
IG_V_gene	Igkv5-48	0.00036256	4.22376866
protein_coding	Spag17	0.0003649	4.29088863
protein_coding	Stum	0.00039248	2.08773563
IG_V_gene	Ighv6-3	0.00040756	11.3571938
protein_coding	Nbeal2	0.00041449	1.68132982
protein_coding	Ajm1	0.00041953	1.72448214
processed_pseudogene	Gm4949	0.00042053	7.64868156
protein_coding	Stfa2l1	0.00042978	2.7303169
protein_coding	Pi16	0.00043563	1.52313466
protein_coding	Pacsin3	0.00043617	2.44586409
protein_coding	Dpep3	0.00043634	5.92231868

© 2023. Nandy A et al. Published online at http://www.aginganddisease.org/EN/10.14336/AD.2023.0510

protein_coding	Neurl2	0.00045336	1.79471652
polymorphic_pseudogene	Mmp1a	0.0004649	6.23305274
lncRNA	Gm15494	0.00048091	5.14330445
protein_coding	Gm10718	0.00049164	28.7141375
TEC	Gm36932	0.00049164	2.54713542
protein_coding	Myo1f	0.00049164	1.59553599
protein_coding	Napb	0.00051922	2.09071295
protein_coding	Tspan11	0.00052609	1.778132
protein_coding	Cxcr3	0.00053955	2.37652143
protein_coding	Ablim2	0.00053957	2.18223215
scaRNA	Scarna17	0.00053957	2.05548459
protein_coding	Ttbk1	0.00057409	2.24390653
processed_pseudogene	Gm6366	0.00057674	33.4271941
protein_coding	Espn	0.0005768	1.8152599
protein_coding	Rasgrf2	0.00058518	1.70310388
protein_coding	Slc26a10	0.00058923	2.01088209
protein_coding	Cacna1a	0.00060941	1.65215587
lncRNA	Gm17110	0.00062493	2.25383923
protein_coding	Ntrk3	0.00062624	2.37197883
protein_coding	Ptpn21	0.00063928	1.67695233
TEC	9330121K16Rik	0.00064319	4.00833065
protein_coding	Map4	0.00064377	1.86390757
lncRNA	2900052L18Rik	0.00066299	1.70197629
protein_coding	Txk	0.0006713	1.89563698
IG_V_gene	Igkv4-61	0.00067375	13.0875063
protein_coding	Zfp641	0.00067375	2.38864192
protein_coding	Gprasp2	0.00068195	1.95597859
protein_coding	Fam214a	0.00069773	1.59648621
protein_coding	Tmem38b	0.00070299	1.62323265
protein_coding	Inmt	0.00070636	2.78080733
protein_coding	Map3k9	0.00071949	1.57354178
protein_coding	Klhl29	0.00074079	1.74563011
protein_coding	Fgf23	0.00074877	2.04787056
protein_coding	Sptbn5	0.00075122	2.10051738
protein_coding	Bmpr1b	0.00075373	2.93586446
lncRNA	Gm14964	0.0007764	3.03001415
protein_coding	Cxcl5	0.00077814	1.89234966
protein_coding	Amer2	0.00077814	1.73932327
TEC	Gm37728	0.00079895	1.60205067
lncRNA	2310016G11Rik	0.00083324	4.10874418
protein_coding	Soat2	0.00086978	1.68410909

© 2023. Nandy A et al. Published online at http://www.aginganddisease.org/EN/10.14336/AD.2023.0510

SUPPLEMENTARY DATA

protein_coding	Bglap3	0.00087803	2.42926277
protein_coding	1810010H24Rik	0.0008833	1.67636406
protein_coding	Tmem191c	0.00089182	1.81985621
protein_coding	BC051142	0.00089567	2.18196482
protein_coding	Alpk3	0.00090156	2.5310189
protein_coding	Actr3b	0.00090156	1.90306744
protein_coding	Hoxa7	0.00090731	1.72372341
protein_coding	Scel	0.0009196	3.39074215
protein_coding	Chrnb1	0.00093951	1.69147631
lncRNA	Gm10382	0.00095335	2.22150221
protein_coding	Gm5150	0.00098699	2.0188433
protein_coding	Ccl5	0.00099675	2.43738746
protein_coding	Myrip	0.00100019	2.15939923
lncRNA	Gm1968	0.00100019	1.93828747
protein_coding	Adgrb1	0.00104285	1.79080197
protein_coding	Tchh	0.00108341	2.05886719
protein_coding	Dnah6	0.00108468	1.52962347
protein_coding	Filip1	0.00108927	2.00019353
protein_coding	Plekha4	0.00109699	1.66071702
protein_coding	Gata2	0.00109699	1.61678934
protein_coding	Rasgrf1	0.00114251	1.80176266
protein_coding	Efemp1	0.00121755	2.44853057
protein_coding	Il21	0.00124193	34.3215406
protein_coding	Nlrc3	0.00124395	1.54440446
protein_coding	Adra1a	0.00128335	2.30981957
protein_coding	Ppp2r2c	0.00133774	4.63249964
protein_coding	Pgr	0.00133774	4.1953441
transcribed_processed_pseudogene	Gm31166	0.00134682	1.73242321
protein_coding	Celsr3	0.00134682	1.50556816
protein_coding	Gdf6	0.00135244	3.71174237
processed_pseudogene	BC023105	0.0013573	2.20468666
protein_coding	Agap2	0.00143789	1.58915662
protein_coding	Catsper4	0.00144825	4.06303121
IG_V_gene	Ighv14-3	0.00145206	2.70928317
protein_coding	H2-T3	0.00145724	27.8620742
protein_coding	Adcy8	0.00145724	6.99473719
protein_coding	Ccno	0.00149019	1.73517532
protein_coding	Gadd45b	0.00149104	1.59343974
lncRNA	Prdm16os	0.00149407	3.10691384
protein_coding	Cnksr1	0.00149407	2.3450744
protein_coding	Ctla4	0.0015001	3.47934969

© 2023. Nandy A et al. Published online at http://www.aginganddisease.org/EN/10.14336/AD.2023.0510

protein_coding	Hecw1	0.00152694	5.3600113
protein_coding	Zc3h6	0.00154476	1.59585231
protein_coding	1600002K03Rik	0.0015586	1.60615713
protein_coding	Npc1l1	0.00155979	2.94591488
protein_coding	Spint2	0.00158562	1.56327685
protein_coding	Als2cl	0.00161523	1.61183509
protein_coding	Fmo2	0.00161856	1.83661097
protein_coding	Mss51	0.00162233	2.11861441
protein_coding	Tnfsf13	0.00162233	1.75679531
lncRNA	Gm11211	0.00165498	2.96866503
protein_coding	Pou4f1	0.00169883	2.89227142
protein_coding	Sfn	0.00173995	1.56527373
protein_coding	Acsbg1	0.00174427	2.71657079
lncRNA	2900005J15Rik	0.00175069	1.53242791
protein_coding	Pgm5	0.00175374	2.15666437
protein_coding	Rassf10	0.00177459	2.24481607
IG_V_gene	Igkv8-30	0.00177549	4.87252483
protein_coding	Sall4	0.00177727	2.7450583
protein_coding	Myo5c	0.00186103	2.62638182
protein_coding	F7	0.00186875	1.95277564
protein_coding	Adra2c	0.00187759	1.99525003
protein_coding	Bin1	0.00187759	1.75643059
protein_coding	Krt80	0.00189529	1.72433802
protein_coding	Zbtb42	0.00191446	1.51354849
protein_coding	Mustn1	0.00191838	1.84736109
lncRNA	Gm5608	0.00196223	2.17876721
protein_coding	Myoz3	0.00197154	2.23129049
protein_coding	Ccdc88b	0.00197154	1.66725222
protein_coding	Pla2g3	0.0019803	2.31696852
protein_coding	Kcnc2	0.001991	1.76816117
protein_coding	Prune2	0.00199513	2.0448812
processed_pseudogene	Gm2830	0.00200678	2.28717814
protein_coding	Egr3	0.00201364	1.53981167
protein_coding	Ccdc9	0.00202384	1.55918484
protein_coding	Bex1	0.00205809	2.2660737
protein_coding	H2-Oa	0.00207313	1.75941948
protein_coding	Csf2ra	0.00211191	1.6583029
protein_coding	Tmem37	0.00212454	2.19219545
protein_coding	Trpm3	0.00212859	1.91530848
processed_pseudogene	Gm14769	0.00213846	3.89515312
protein_coding	Hoxb5	0.00215589	1.92791904

© 2023. Nandy A et al. Published online at http://www.aginganddisease.org/EN/10.14336/AD.2023.0510

protein_coding	Cry2	0.00215589	1.57523245
protein_coding	Slc12a5	0.0021566	1.61774512
protein_coding	Kcnj6	0.00217109	3.12950916
protein_coding	Tmem45a2	0.00220592	2.33027024
protein_coding	Lgr6	0.00221591	1.56678792
IG_V_gene	Igkv6-23	0.00222493	4.92783476
protein_coding	Sidt1	0.0022298	1.65300165
protein_coding	Prob1	0.00224657	2.20305928
protein_coding	Clip1	0.0022762	1.61425865
lncRNA	Gm12523	0.00230537	2.24022894
protein_coding	Aox1	0.00234551	1.95011748
protein_coding	Col4a5	0.0023592	2.22231842
protein_coding	Grhl1	0.0023592	1.84548827
protein_coding	Hlf	0.00236932	1.59050648
protein_coding	Ppp1r26	0.00238978	1.51109154
protein_coding	Gm7609	0.00243023	11.0501693
protein_coding	Ctnna3	0.00243094	2.27193625
protein_coding	Cxcr5	0.00246342	1.65153127
protein_coding	Arhgap9	0.00246342	1.58682678
protein_coding	Slc1a1	0.00246494	2.70402595
protein_coding	Hepacam2	0.00248976	2.64857568
IG_V_gene	Igkv4-70	0.00251671	9.50133315
protein_coding	Adamtsl5	0.00253707	2.00027307
protein_coding	Muc3a	0.00260663	2.19071876
protein_coding	Bhlhe41	0.00260663	1.59962256
protein_coding	Csmd2	0.00261962	5.00136543
protein_coding	Ifi27l2b	0.00264797	7.12792676
protein_coding	Cd274	0.0026582	1.79251807
protein_coding	Aldh1l1	0.00266672	1.94420628
protein_coding	Xntrpc	0.00270695	2.33280215
protein_coding	Adra1d	0.00273945	1.94027101
protein_coding	Mmp25	0.00275624	1.52740769
TR_C_gene	Trac	0.00276064	2.47235817
processed_pseudogene	Gm38158	0.00276158	2.12796529
protein_coding	Sema4f	0.00282922	1.65489214
protein_coding	Ccdc85a	0.00283972	2.3520157
protein_coding	Insl3	0.00284228	1.99370303
protein_coding	Hk2	0.00288192	1.74240536
protein_coding	Bcam	0.00289906	1.82889228
protein_coding	Top1mt	0.00290526	1.52506494
protein_coding	Ager	0.00296797	1.5917382

© 2023. Nandy A et al. Published online at http://www.aginganddisease.org/EN/10.14336/AD.2023.0510

protein_coding	Adh6b	0.00301976	1.92308699
protein_coding	Fam71b	0.00302438	2.14100604
protein_coding	Ubxn10	0.00305521	3.82901507
protein_coding	Lsmem1	0.00309645	2.28780524
protein_coding	Carns1	0.00316172	1.50400408
protein_coding	Awat1	0.00322034	20.9816308
protein_coding	Ggt1	0.00324979	1.90977639
protein_coding	Nlrp3	0.00333182	1.54797221
protein_coding	Herc3	0.00335745	1.53501397
protein_coding	Fezf2	0.00337887	3.94197469
protein_coding	Dmd	0.00342446	2.14341244
protein_coding	Prkn	0.0034394	1.8148834
lncRNA	Gm17034	0.00345698	1.65942188
protein_coding	Nes	0.00345748	2.12342287
TEC	Gm43185	0.0034677	2.14780213
protein_coding	Ier2	0.0034677	1.5422001
protein_coding	Trim7	0.00348268	1.67794813
lncRNA	Gm16170	0.00354526	1.93091589
protein_coding	Xkr6	0.00361963	1.96460523
lncRNA	C4a	0.00365813	1.58071236
protein_coding	Sbk3	0.00366838	4.63966625
protein_coding	Ccr6	0.00370552	2.76270694
protein_coding	Golga4	0.00370552	1.74168759
protein_coding	Itgal	0.00370552	1.5784361
protein_coding	Mab21l1	0.00373841	1.87051932
IG_V_gene	Ighv1-63	0.00376307	16.7338539
TEC	AC124502.1	0.00379135	2.02350374
protein_coding	Ovgp1	0.00380838	1.5516645
protein_coding	Cpeb1	0.00383487	1.69625739
TEC	Gm42432	0.00393364	1.89369058
protein_coding	Vwa3b	0.0039763	2.01101442
protein_coding	Fbxo32	0.00399336	2.03317719
protein_coding	Llgl2	0.00400916	1.5078377
protein_coding	C1rl	0.00406795	1.5220698
protein_coding	Ptchd1	0.00416385	2.09465308
protein_coding	Ccdc9b	0.00416487	1.61629856
protein_coding	Zfp365	0.00422664	1.89691405
protein_coding	Hoxb6	0.0042474	1.96265529
IG_V_gene	Ighv1-26	0.00428897	2.42314581
protein_coding	Syt7	0.00429762	1.73189483
protein_coding	Tceal5	0.00437499	2.32380883

© 2023. Nandy A et al. Published online at http://www.aginganddisease.org/EN/10.14336/AD.2023.0510

lncRNA	Gm20544	0.00437585	1.74906327
protein_coding	Cul9	0.00442543	1.50012171
protein_coding	Kri1	0.00447045	1.51270383
protein_coding	Golga7b	0.00450471	2.01060918
protein_coding	Ifitm1	0.0045976	1.54464497
unprocessed_pseudogene	Gm2389	0.00467937	4.13969853
protein_coding	Tenm2	0.00480966	2.2632278
protein_coding	Galr1	0.0048569	19.9342268
protein_coding	Vmn2r97	0.00487561	1.80381778
protein_coding	Rinl	0.00493954	1.55366705
protein_coding	Inpp5j	0.00501067	1.51692926
protein_coding	Robo3	0.00501593	2.27361414
protein_coding	Inafm1	0.00504129	1.67580954
IG_V_gene	Ighv6-6	0.00511734	3.56704029
protein_coding	Ildr1	0.00517971	2.1192416
protein_coding	Klhl41	0.0052317	2.13098091
lncRNA	Tmem51os1	0.0052317	1.95662696
protein_coding	Rgs4	0.0052317	1.64427549
protein_coding	Hook2	0.0052317	1.53880456
protein_coding	Ankrd33b	0.00526589	1.543568
protein_coding	Prodh	0.00529085	1.64744361
protein_coding	Dlgap2	0.00536294	2.55877078
protein_coding	Plec	0.00543618	1.63545025
protein_coding	AC147806.2	0.0054913	2.9473261
protein_coding	Casz1	0.00557106	1.61627417
protein_coding	Hsf4	0.0056352	1.73485166
lncRNA	Irx3os	0.00564982	1.50091515
lncRNA	AC241534.1	0.00579655	1.77227093
protein_coding	Heatr9	0.0058282	2.1070357
protein_coding	Ucn2	0.00593492	3.63109631
lncRNA	Gm26569	0.00593571	1.84597875
protein_coding	Olfr60	0.00596258	19.5541749
protein_coding	Mink1	0.00614872	1.51504582
protein_coding	Pitx2	0.00632053	2.42223929
protein_coding	Arrb2	0.00633451	1.51441424
protein_coding	Pramef12	0.00633565	6.02173407
protein_coding	Akap6	0.00633565	1.79663065
protein_coding	Slc7a15	0.00639701	2.16172403
protein_coding	Ros1	0.00643788	4.95074943
lncRNA	Gm47782	0.00655006	9.33785473
TEC	A230085B16Rik	0.00657148	4.86473846

© 2023. Nandy A et al. Published online at http://www.aginganddisease.org/EN/10.14336/AD.2023.0510

protein_coding	Derl3	0.00657148	2.08011775
protein_coding	Frmpd2	0.00660089	3.1164037
protein_coding	Ppl	0.0066353	1.85829692
lncRNA	9930014A18Rik	0.00667529	2.00158685
processed_pseudogene	Hspd1-ps4	0.00670362	2.26003266
protein_coding	Asb15	0.00670362	2.19549773
protein_coding	Ppip5k1	0.00670362	1.65284135
protein_coding	Rims4	0.00675058	2.26906777
protein_coding	Samd3	0.00677899	2.21062135
protein_coding	Pclo	0.00681096	2.2341772
lncRNA	Gm12688	0.00681871	6.66420136
protein_coding	Apol10b	0.00686809	2.35610189
protein_coding	Neil2	0.00696746	2.04210026
protein_coding	L3mbtl4	0.00704264	5.40568228
lncRNA	Gm32014	0.00704264	1.91567612
protein_coding	Aire	0.00715802	5.58886192
lncRNA	A530040E14Rik	0.00716523	1.89078622
protein_coding	Mfsd7a	0.00717699	1.55063847
lncRNA	Gm35551	0.00720097	4.29442434
unprocessed_pseudogene	Gm8979	0.00720872	2.17460639
IG_V_gene	Ighv1-82	0.00725323	1.94259903
protein_coding	Fam184a	0.00725618	1.60072731
protein_coding	Vwa3a	0.0073251	2.37443246
protein_coding	Cd101	0.0073251	1.52871056
TEC	Gm38197	0.00734625	2.67225991
protein_coding	Sptbn4	0.00734886	2.01506818
protein_coding	Olfr98	0.00736319	8.67363911
lncRNA	5033421B08Rik	0.0073702	2.12731505
protein_coding	En2	0.00740809	3.72472841
protein_coding	Cacna1i	0.00760896	1.55466153
protein_coding	Dact2	0.0076238	1.9159191
protein_coding	Wfs1	0.00778128	1.71332504
protein_coding	Lim2	0.00785771	4.80576868
protein_coding	Tpm3-rs7	0.00785771	2.49766032
protein_coding	Vegfa	0.00785771	1.67415097
lncRNA	Gm39323	0.00790674	2.84838873
protein_coding	Krt86	0.00794249	1.69125267
protein_coding	Sh3rf2	0.00798303	2.16245256
IG_V_gene	Igkv3-5	0.00800132	9.16907396
protein_coding	Hephl1	0.00800132	3.71386612
protein_coding	Trpc1	0.00800132	1.85656093

© 2023. Nandy A et al. Published online at http://www.aginganddisease.org/EN/10.14336/AD.2023.0510

protein_coding	Uaca	0.00800519	1.77944234
protein_coding	Insyn1	0.00802378	1.64564428
protein_coding	Zic1	0.00806961	3.35085373
protein_coding	Rims2	0.00807691	2.09061776
protein_coding	Plin5	0.00829327	1.90943767
unprocessed_pseudogene	Gm20789	0.00830765	3.60545945
protein_coding	Rnft2	0.00831958	1.66800377
protein_coding	Gm11639	0.00839373	2.22746494
TEC	8430422M14Rik	0.00847658	1.97317024
protein_coding	Adprhl1	0.00863004	2.04698383
protein_coding	Dhdh	0.00865678	1.58605911
protein_coding	Tfcp2l1	0.00867605	2.13478109
protein_coding	Myom1	0.00869799	1.91710109
protein_coding	Nrg2	0.00873472	1.64138167
protein_coding	Synm	0.00877012	2.0156433
processed_pseudogene	AC122821.1	0.00895127	3.93560219
protein_coding	Crim1	0.00898086	1.55329712
TEC	Cpeb1os1	0.00905544	2.28261328
protein_coding	Hoxc4	0.00905602	1.52635818
processed_pseudogene	Gm11830	0.00924064	13.7701121
protein_coding	Insrr	0.00925654	3.5536512
protein_coding	Pex11a	0.00933471	1.52173536
protein_coding	Pabpc1l	0.00934182	1.69438545
protein_coding	Slc15a5	0.00934559	2.45926259
protein_coding	Kcns3	0.00934559	2.31750688
protein_coding	Abca4	0.00936144	1.77301391
lncRNA	A330023F24Rik	0.00946182	1.57142762
protein_coding	Stx4a	0.00950931	1.50184238
protein_coding	Slc4a10	0.00952581	2.29938291
protein_coding	Syt15	0.00958466	1.51385329
lncRNA	Gm7854	0.00958591	1.88766574
protein_coding	Tbc1d4	0.00958591	1.61983394
protein_coding	Trip10	0.00967034	1.68367796
protein_coding	Kif13a	0.00967237	1.5085888
protein_coding	Tnik	0.00970489	1.59268791
protein_coding	Nrap	0.00974591	2.15346842
protein_coding	Apobr	0.00983631	1.58766051
protein_coding	Srcin1	0.00985073	1.68879647
protein_coding	Tmc4	0.00985073	1.54620321
protein_coding	Vmn2r96	0.00986497	2.8178445
lncRNA	Gm13206	0.01001753	2.37115703

© 2023. Nandy A et al. Published online at http://www.aginganddisease.org/EN/10.14336/AD.2023.0510

protein_coding	Gpr20	0.01016013	2.4594522
protein_coding	Tacstd2	0.01018249	1.67683247
protein_coding	Cd300lf	0.01023588	1.50569251
lncRNA	Gm15222	0.01035079	1.52996261
protein_coding	Slitrk4	0.01056992	3.60819544
protein_coding	Wnk3	0.01062009	1.55479588
lncRNA	Gm34804	0.01066939	3.10024052
protein_coding	Habp2	0.01077952	2.38222044
protein_coding	Cryab	0.01079303	2.11891282
protein_coding	Rbm47	0.01079303	1.50364889
lncRNA	Gm50316	0.01081	4.9281449
protein_coding	Clnk	0.01083854	2.29433704
protein_coding	Lrat	0.01084777	7.998982
protein_coding	Lhcgr	0.0110383	2.92479044
protein_coding	Asic2	0.01104662	5.57934415
protein_coding	Unc79	0.01110095	1.86934807
protein_coding	Hspa1l	0.01110283	2.001833
lncRNA	Gm15984	0.01112896	4.94206538
IG_V_gene	Ighv5-6	0.01114331	3.14146253
protein_coding	Kiss1r	0.01117345	1.63148026
protein_coding	Il1rapl2	0.01119506	6.58929776
lncRNA	Gm15706	0.01119771	1.81037547
protein_coding	Cysltr2	0.01123903	1.578576
protein_coding	2310030G06Rik	0.01128482	1.8018934
protein_coding	Pcp4l1	0.01137031	1.72820595
protein_coding	Lrg1	0.01142579	1.5266071
protein_coding	Gm8909	0.01145473	8.95917325
protein_coding	Uts2b	0.01169822	4.84308745
protein_coding	Hcar2	0.01169822	2.50899273
protein_coding	Kcnj3	0.01182295	2.19540851
protein_coding	Abcb1a	0.01186215	1.5491054
protein_coding	Acsm5	0.01202518	4.0135416
protein_coding	Zfr2	0.01215664	1.50507875
protein_coding	Erich3	0.01220998	3.35565156
protein_coding	Myo5b	0.01220998	2.08085817
lncRNA	Gm17197	0.01225972	3.00548749
lncRNA	4932441J04Rik	0.01229663	1.53590544
processed_pseudogene	Gm2445	0.012319	3.73852092
protein_coding	Fyb	0.012319	1.58007516
lncRNA	Hectd2os	0.01253062	2.48610991
protein_coding	Klhl38	0.01259657	2.40838023

protein_coding	Nol3	0.01260654	2.00135544
protein_coding	Cacng8	0.012774	3.75168698
lncRNA	Gm15870	0.01281292	2.68948016
protein_coding	Hoxa4	0.01281923	1.71019369
protein_coding	Cngb1	0.01281923	1.707817
IG_V_gene	Igkv3-10	0.01287896	4.60946079
protein_coding	Alox15	0.01288623	1.86904352
protein_coding	Zbtb32	0.01299333	1.59184877
protein_coding	Ankrd35	0.01303631	1.72283934
protein_coding	Wdr95	0.01305907	2.60141736
protein_coding	Nuggc	0.01313318	2.3598024
protein_coding	Mtus2	0.01313318	1.88088348
lncRNA	Gm41381	0.01314468	1.54034582
lncRNA	Mecomos	0.01321857	2.18725085
protein_coding	Mecom	0.01321857	1.53368655
protein_coding	Retnla	0.01337659	2.18086445
protein_coding	Mybpc2	0.0134506	1.99333661
protein_coding	Lamc3	0.01356368	1.6419695
protein_coding	Npas4	0.01356755	2.0542389
protein_coding	Fam135b	0.01357428	2.53570595
protein_coding	Gzmm	0.01357428	1.59683621
protein_coding	Etfb	0.01358909	1.51600641
protein_coding	Tmem82	0.01359689	2.1587157
protein_coding	Rbm24	0.01364622	1.85743433
lncRNA	Gm28187	0.01378991	1.6046925
protein_coding	Pcdhga2	0.01403918	1.55251555
protein_coding	Scn8a	0.01441107	2.10172167
processed_pseudogene	Mospd4	0.01457739	2.49257647
protein_coding	Atp2b2	0.01460007	1.6112967
TEC	Gm44981	0.01469055	1.56151146
protein_coding	F830016B08Rik	0.01485785	1.74483214
protein_coding	Rbfox1	0.01491517	1.85083778
transcribed_processed_pseudogene	Gm2099	0.01494044	3.25820822
protein_coding	Tcap	0.01505427	2.0266643
protein_coding	Fhad1	0.01508092	3.79922001
processed_pseudogene	Gm7895	0.01514138	2.44782475
lncRNA	Gm26604	0.01520572	2.5323267
protein_coding	1700001O22Rik	0.01523791	2.34170519
protein_coding	Rab37	0.01523791	1.516358
lncRNA	I730030J21Rik	0.0152824	1.93972128
lncRNA	B430219N15Rik	0.01530002	2.09692094

protein_coding	Arhgap44	0.0153301	1.90020215
unitary_pseudogene	Gm46575	0.01536319	4.17160872
protein_coding	Sbk2	0.01538376	1.89111824
lncRNA	Gm40916	0.01546152	2.00489432
protein_coding	Ecel1	0.01560612	4.93212332
protein_coding	Hspb8	0.01566246	1.86356771
protein_coding	Eid3	0.0156669	1.59754575
protein_coding	Lmod1	0.01568932	1.65070721
protein_coding	Lrrc2	0.01574006	2.10294914
protein_coding	Lrrc3b	0.01595922	2.87688737
protein_coding	Tekt1	0.01604173	2.37826107
lncRNA	4930417O22Rik	0.01609897	2.70532331
protein_coding	Gm21988	0.01611454	1.80753434
TR_C_gene	Trbc1	0.0161465	1.89493956
processed_pseudogene	Gm9525	0.01618482	2.11015922
protein_coding	Ablim1	0.01624106	1.58891472
protein_coding	Slc16a9	0.01642154	1.69497244
protein_coding	Cd3d	0.01643348	2.28291419
protein_coding	Sycp2	0.016542	1.63682542
protein_coding	Drc3	0.0168097	1.63414328
protein_coding	Pacrg	0.01680988	2.61161719
TEC	Gm10513	0.01680988	1.9028356
protein_coding	Med12l	0.01682749	1.64112902
protein_coding	Kcnd3	0.0168588	2.8251006
protein_coding	Scube3	0.01694577	1.53362649
IG_V_gene	Ighv1-20	0.01702631	20.5987408
protein_coding	Sorbs1	0.01704181	1.58516163
protein_coding	Ryr1	0.01708454	2.02739189
protein_coding	Eppk1	0.01731361	1.86132718
protein_coding	Fgf13	0.01740693	1.54541628
miRNA	Gm25301	0.017425	2.15871699
protein_coding	Sv2a	0.0174804	1.53167981
protein_coding	Gm3045	0.0175516	12.219321
lncRNA	Gm9869	0.0175917	1.69679226
protein_coding	Epcam	0.01759982	2.18352549
protein_coding	Gm4841	0.01759982	1.92789989
protein_coding	Pla2g2d	0.01760002	2.73238229
lncRNA	Gm42722	0.01760002	2.5853493
protein_coding	Zfyve28	0.01760002	1.53516194
protein_coding	Prf1	0.01773413	1.7359854
protein_coding	Itga7	0.01784363	1.79980296

© 2023. Nandy A et al. Published online at http://www.aginganddisease.org/EN/10.14336/AD.2023.0510

protein_coding	Acadm	0.01797977	1.64105902
lncRNA	Gm37027	0.01806796	1.8155978
protein_coding	Abra	0.01808939	2.00368565
protein_coding	Nqo1	0.01839235	1.66578758
protein_coding	Sec14l5	0.01845031	1.87901984
protein_coding	Speg	0.01856846	1.95602926
protein_coding	Ccdc112	0.01862113	1.70358852
lncRNA	Gm49492	0.01877735	1.76191501
protein_coding	Spink11	0.01878363	9.02863437
snoRNA	Gm24519	0.01881168	1.96076392
protein_coding	Fasl	0.01896537	2.50603188
lncRNA	1700021J08Rik	0.01904261	2.86680532
protein_coding	Dupd1	0.01904261	2.16877135
processed_pseudogene	Gm5628	0.01904261	2.02510654
TEC	Gm42141	0.0191766	1.78828652
protein_coding	Pld5	0.01939729	2.6565993
scaRNA	Scarna2	0.01939729	1.73924096
protein_coding	Ccdc148	0.01945102	2.04688347
protein_coding	Tarm1	0.01955745	1.69612658
miRNA	Gm25747	0.01958119	1.98052962
processed_pseudogene	Gm8514	0.01960112	2.96732014
lncRNA	Gm14199	0.01977953	19.0802798
lncRNA	Gm8066	0.01979435	1.59911787
IG_V_gene	Igkv6-17	0.01981033	4.99143347
protein_coding	Esrra	0.01981066	1.50719475
lncRNA	Gm13373	0.01994479	2.35979844
protein_coding	Crybb1	0.01998775	1.95543313
miRNA	Mir133a-2	0.02000895	2.32976874
protein_coding	Fbp2	0.02022515	2.04314534
lncRNA	Notumos	0.02023845	2.60240495
protein_coding	Myom3	0.02041017	3.4955064
processed_pseudogene	Gm7061	0.02045352	1.82433699
TEC	Gm45743	0.02059751	1.67301847
protein_coding	Kif1c	0.02063815	1.70620367
TEC	Gm37024	0.02066252	2.84515018
processed_pseudogene	Akirin1-ps	0.02068961	1.77292864
lncRNA	Gm32849	0.02070978	3.62457694
protein_coding	Slc11a1	0.02079024	1.55746535
protein_coding	Kif5a	0.02079024	1.54496231
protein_coding	Ryr3	0.02084929	2.20708156
protein_coding	Txlnb	0.02086948	1.98492297

© 2023. Nandy A et al. Published online at http://www.aginganddisease.org/EN/10.14336/AD.2023.0510

protein_coding	Dcst1	0.02091792	1.50731178
protein_coding	Tmem252	0.02101381	1.99627276
IG_V_gene	Igkv9-124	0.02108394	3.60536898
processed_pseudogene	Gm4840	0.02113963	2.31848471
protein_coding	Olfr99	0.02113963	1.68467161
IG_V_gene	Igkv3-2	0.0211497	7.50776399
lncRNA	Gm7706	0.02147929	12.1703402
lncRNA	Gm20758	0.021487	4.68441166
transcribed_processed_pseudogene	4931440P22Rik	0.02173272	1.61488695
lncRNA	Gm43646	0.02189229	2.75010026
processed_pseudogene	Gm11408	0.02214414	2.8650654
protein_coding	AC167036.1	0.0224036	1.91589406
protein_coding	Ltb	0.0224036	1.61549463
protein_coding	Cmya5	0.02250852	1.86415764
lncRNA	Gm49906	0.02256519	2.5014387
protein_coding	Map1b	0.02261512	1.58647487
lncRNA	Gm26947	0.02262727	1.57804633
processed_pseudogene	Gm6659	0.02271266	6.37574494
lncRNA	4933432I09Rik	0.022737	2.73206271
lncRNA	Trerf1	0.02273876	1.50844238
protein_coding	Aldh5a1	0.02274538	1.56516655
protein_coding	S1pr5	0.02283277	2.25968347
protein_coding	Map1a	0.02321213	1.87363707
protein_coding	Nhlrc1	0.02331975	1.78194331
lncRNA	Gm12002	0.02336643	1.7655432
lncRNA	5031425F14Rik	0.02341735	1.88332354
protein_coding	Gabrr1	0.02346405	3.98805548
protein_coding	Crip3	0.02363938	1.70055035
protein_coding	Fbxo40	0.02374138	1.80658556
lncRNA	Fendrr	0.0237603	2.76657209
lncRNA	F730043M19Rik	0.02389186	1.74345682
protein_coding	Prx	0.02390784	1.52688045
lncRNA	Gm33104	0.02426727	2.38172275
protein_coding	Oosp1	0.0243768	5.92679362
lncRNA	Gm15912	0.02439083	1.76115443
misc_RNA	Gm23563	0.02461144	2.39843342
protein_coding	Clec2g	0.02462702	1.74720726
protein_coding	Ksr1	0.02472061	1.61729615
processed_pseudogene	Rpl31-ps5	0.02491946	2.98478116
protein_coding	Ppp1r12b	0.02494504	1.55600721
TEC	Gm37767	0.02505823	6.8052818

© 2023. Nandy A et al. Published online at http://www.aginganddisease.org/EN/10.14336/AD.2023.0510

protein_coding	Mfsd6l	0.02507995	1.58218541
protein_coding	Cltb	0.02508482	1.50537362
protein_coding	Gm12216	0.02508921	1.56806083
processed_pseudogene	Gm6252	0.02511261	2.7112193
IG_V_gene	Ighv3-6	0.02526873	2.3256291
protein_coding	Cryba4	0.02534029	1.96482149
IG_V_gene	Igkv1-117	0.02542021	2.04837973
protein_coding	1-Mar	0.02542021	1.51506707
protein_coding	Eno4	0.0254609	2.22234089
TR_J_gene	Traj15	0.02547621	5.43726223
protein_coding	Wipf3	0.02558535	1.61855625
protein_coding	Slco5a1	0.02564373	1.81671433
protein_coding	Maob	0.02569955	1.77921279
protein_coding	Kcnq4	0.02573607	1.97674367
protein_coding	Rbpms2	0.02578574	1.61107036
protein_coding	Gm9918	0.0258595	11.0211073
lncRNA	Gm28653	0.02591365	5.9136514
TR_C_gene	Trgc4	0.02619349	3.24147076
protein_coding	Art5	0.02619349	1.8751598
lncRNA	9130015G15Rik	0.02639877	5.74709009
protein_coding	Gm1043	0.02657891	1.76006218
protein_coding	Cd28	0.02681434	1.94981587
lncRNA	2310005E17Rik	0.02693031	8.04309728
lncRNA	Gm11264	0.02701012	3.53474215
protein_coding	Fchsd1	0.02719563	1.51811206
protein_coding	Prrt4	0.02721583	2.55114534
protein_coding	Map3k20	0.02721995	1.61498092
lncRNA	Gm46224	0.02724457	1.71044383
processed_pseudogene	Gm8185	0.02725034	1.54949316
lncRNA	Gm13441	0.02740414	1.64932763
protein_coding	Scn2a	0.02750043	1.66418697
protein_coding	Chrm3	0.02773303	1.62263059
protein_coding	Kif3c	0.02802977	1.52978908
protein_coding	Il2rb	0.02821788	1.78306265
protein_coding	Stk32b	0.02835676	2.40012231
processed_pseudogene	Gm17047	0.02852083	11.1243025
lncRNA	Gm36070	0.02877341	4.71910805
protein_coding	Il17re	0.02879428	2.10105045
protein_coding	Cd247	0.02897395	1.56974622
lncRNA	9230112J17Rik	0.02905712	2.05736107
processed_pseudogene	Gm11662	0.02916928	1.66244004

© 2023. Nandy A et al. Published online at http://www.aginganddisease.org/EN/10.14336/AD.2023.0510

protein_coding	H2-DMb2	0.02921375	1.5336905
protein_coding	Ociad2	0.02928154	1.74031811
lncRNA	Gm15935	0.02938961	2.52682082
IG_V_gene	Ighv5-17	0.02944193	3.75309035
protein_coding	Vwa7	0.02944369	1.68284986
protein_coding	Slc47a2	0.02961439	8.76878968
protein_coding	Prss16	0.02969566	1.73146231
processed_pseudogene	Gm12098	0.02973413	1.87369748
lncRNA	A430093F15Rik	0.02986351	2.03138445
processed_pseudogene	Gm18609	0.02988029	2.22922764
protein_coding	Ablim3	0.03004469	1.74290333
protein_coding	Ugt1a2	0.03011015	1.74175627
protein_coding	Cpne4	0.03011708	5.01763733
processed_pseudogene	Gm8233	0.03033024	4.82938573
IG_V_gene	Igkv14-126	0.03033024	4.43670605
protein_coding	Efcab8	0.03052891	1.69538684
protein_coding	Adcy5	0.03061401	1.59730671
protein_coding	Rassf9	0.0306323	1.9361802
protein_coding	Mycn	0.03071014	1.78282931
protein_coding	Synpo2l	0.0308357	2.3893772
polymorphic_pseudogene	Clec7a	0.0308357	1.5560591
protein_coding	Gsta3	0.03090052	1.73214694
protein_coding	Ngfr	0.03106005	1.87724296
protein_coding	Rorc	0.03109497	1.99039947
protein_coding	Rimkla	0.03112782	2.18981902
protein_coding	Tead4	0.03127848	1.62750154
IG_V_gene	Igkv6-20	0.03131476	8.57368803
protein_coding	Vinac1	0.03150188	1.79808912
protein_coding	Ldhb	0.03151831	1.96509192
protein_coding	Itih3	0.03159565	3.28408919
protein_coding	Sec31b	0.03159565	1.64293432
lncRNA	Gm44421	0.03190499	2.23275181
lncRNA	Gm38171	0.03193766	1.87522107
protein_coding	Vipr1	0.03202466	1.5237219
IG_V_gene	Igkv2-109	0.03219905	1.99305386
protein_coding	Ffar1	0.03245997	1.636001
lncRNA	Gm15523	0.03268593	1.89487648
protein_coding	Jag2	0.03277549	1.6135494
unprocessed_pseudogene	9130208D14Rik	0.03292559	1.9943845
protein_coding	Ccdc85c	0.03292559	1.73598819
protein_coding	Srrm4	0.03320806	2.04802736

© 2023. Nandy A et al. Published online at http://www.aginganddisease.org/EN/10.14336/AD.2023.0510

protein_coding	Tacc2	0.03320806	1.82874241
protein_coding	Des	0.03325651	1.92092913
protein_coding	Ppp1r14a	0.03332206	1.61412918
protein_coding	Slc25a34	0.0333332	2.934144
protein_coding	Fam163b	0.03377615	2.27231264
lncRNA	Gm5860	0.03402379	1.82340295
protein_coding	Mlycd	0.03404496	1.63937561
protein_coding	Flnc	0.03418729	1.81403308
protein_coding	Stau2	0.03432117	1.53735214
protein_coding	Shh	0.0343315	15.1962344
protein_coding	Fam131a	0.03438963	1.63094262
protein_coding	Sh2d4b	0.03484835	1.60080802
protein_coding	Cdkl2	0.03486546	1.7474574
protein_coding	Rimklb	0.03490781	3.70842667
protein_coding	A530064D06Rik	0.03498254	1.55031641
IG_V_gene	Ighv14-4	0.03519766	5.88708578
protein_coding	Zdhhc23	0.03528952	1.69746108
protein_coding	Mrgpra2b	0.03557878	1.78922796
protein_coding	Upk1a	0.03563539	2.01313182
lncRNA	Gm14261	0.03572869	2.7523271
protein_coding	Gipr	0.03585962	1.91803915
protein_coding	Cacng1	0.03585962	1.8264531
protein_coding	Aqp7	0.03588908	1.89372173
protein_coding	Ppargc1a	0.03635417	1.83749507
protein_coding	Lmntd1	0.0363814	4.21959127
protein_coding	Mylk4	0.03641417	1.83976634
protein_coding	Ptpn3	0.03646714	1.60628127
lncRNA	Gm9899	0.03649808	3.08628358
protein_coding	Stac2	0.03649808	1.59890638
protein_coding	Ccdc92	0.0365372	1.71092979
lncRNA	Gm44175	0.03673958	2.03402771
protein_coding	Rab11fip3	0.03673958	1.54955998
lncRNA	D830032E09Rik	0.03693356	6.36144459
protein_coding	Ptpn5	0.03701463	2.32417423
lncRNA	Gm15943	0.0371289	2.23644817
protein_coding	Kcna3	0.03715306	1.63540069
protein_coding	Pifo	0.03722726	2.31345072
lncRNA	Gm9750	0.03722726	2.00888446
lncRNA	Gm536	0.0372479	1.98094752
protein_coding	Tmem8b	0.03759091	1.59394362
lncRNA	1700018A04Rik	0.03769184	5.51571639

© 2023. Nandy A et al. Published online at http://www.aginganddisease.org/EN/10.14336/AD.2023.0510

protein_coding	Tspan10	0.03784725	1.92953244
lncRNA	Gm13943	0.03804856	2.55266471
protein_coding	1700020L24Rik	0.03816667	1.5321453
protein_coding	Cfap70	0.03825483	1.55298488
rRNA	n-R5s25	0.03844122	3.2199519
TR_C_gene	Trbc2	0.03856817	2.0126733
transcribed_unprocessed_pseudogene	Gm4956	0.03856817	1.93405313
protein_coding	Hoxb8	0.03856817	1.59502098
protein_coding	Pabpn1	0.03876923	1.61295436
protein_coding	Stat4	0.03889106	1.52481044
protein_coding	Idh2	0.03889576	1.65602546
protein_coding	Dynll2	0.03897562	1.59459305
protein_coding	Prkag3	0.03907799	1.859406
protein_coding	Fam184b	0.03907799	1.67564743
protein_coding	Pnck	0.03907799	1.55899338
protein_coding	Jak3	0.03907799	1.51122513
protein_coding	Tubb1	0.03918332	1.55452531
miRNA	Mir1190	0.03920536	1.76058994
protein_coding	Nr2e3	0.03928339	3.91850444
protein_coding	Obscn	0.03937194	2.35463289
unprocessed_pseudogene	Gm13284	0.03937464	6.02344375
processed_pseudogene	Gm12366	0.03937731	1.61256849
lncRNA	Gm41717	0.03940943	3.04830069
processed_pseudogene	Gm18068	0.03943609	1.51962669
IG_V_gene	Igkv10-96	0.03946276	2.36977007
protein_coding	Hcn4	0.03946757	1.94498062
processed_pseudogene	Gm7357	0.0395287	2.32345644
Mt_tRNA	mt-Ti	0.0395287	2.29302073
protein_coding	Ly6g5b	0.03959075	1.57822677
protein_coding	Nrn1	0.03966514	2.08536329
IG_V_gene	Igkv13-84	0.03968293	5.02693827
protein_coding	Lpl	0.03973898	1.73790863
lncRNA	Gm10553	0.03984706	3.02389866
protein_coding	Slc39a4	0.03985458	1.78489632
protein_coding	Zfp853	0.04052267	1.53867277
protein_coding	Foxq1	0.04076372	2.98951206
protein_coding	Gm19345	0.04084583	2.06423884
protein_coding	Upk1b	0.04135765	2.22860321
protein_coding	Cdhr4	0.04164489	1.57261302
protein_coding	Lhfpl3	0.04166266	5.01293318
IG_V_gene	Igkv1-110	0.04170457	3.62984505

© 2023. Nandy A et al. Published online at http://www.aginganddisease.org/EN/10.14336/AD.2023.0510

protein_coding	Coro6	0.04175926	1.79206636
TEC	Gm48296	0.04189724	1.94990943
miRNA	Mir7687	0.04189724	1.82392816
protein_coding	Ppfia3	0.041951	1.93655099
protein_coding	Slc7a3	0.04207129	1.86041719
protein_coding	Dpyd	0.04226696	1.56948492
protein_coding	Gys1	0.04269316	1.56781896
protein_coding	Dst	0.04274259	1.5239501
lncRNA	Gm47381	0.04300918	4.35881371
lncRNA	Gm13429	0.04310176	17.341473
processed_pseudogene	Gm14830	0.04323902	2.35020191
protein_coding	Gzmk	0.04340691	3.6990084
protein_coding	Lrrc30	0.04344884	1.79369592
protein_coding	Myh13	0.04377437	2.33258333
protein_coding	Cfap57	0.04388883	1.66457152
lncRNA	Hoxb5os	0.04418959	1.94774066
protein_coding	Ogdhl	0.0443042	1.91579758
protein_coding	Klhl40	0.04432579	1.85219079
lncRNA	Gm28265	0.04457066	1.84865858
protein_coding	Ttll7	0.04468668	1.70652969
protein_coding	Hmgcs2	0.04504721	1.78057964
lncRNA	Gm20757	0.04545248	11.7485997
protein_coding	Nap1l3	0.04564064	1.79545085
unprocessed_pseudogene	Gm7115	0.04568633	5.56478516
protein_coding	Ostn	0.04580447	1.79076797
protein_coding	Dgat2l6	0.04599606	3.81961549
processed_pseudogene	Gm8734	0.04605386	7.80857728
protein_coding	Mef2c	0.04605386	1.57171571
lncRNA	Gm16050	0.04608783	5.90223996
lncRNA	6330418K02Rik	0.04626301	1.54216983
protein_coding	Cand2	0.04663693	1.63189442
processed_pseudogene	Gm5276	0.04669463	3.26025779
protein_coding	Lat	0.04669463	1.77034194
protein_coding	Synpo2	0.04683814	1.78108244
lncRNA	Gm37759	0.04687341	2.66471015
protein_coding	Rimbp3	0.04699591	1.76177496
protein_coding	Cd4	0.04720324	1.93840501
protein_coding	Ttll11	0.04720847	1.51761024
processed_pseudogene	Gm18911	0.04727073	5.38939319
protein_coding	Fam57b	0.04729254	1.62779387
processed_pseudogene	Gm7498	0.04752291	2.27968896

© 2023. Nandy A et al. Published online at http://www.aginganddisease.org/EN/10.14336/AD.2023.0510

protein_coding	Ankdd1a	0.0477073	1.7398424
processed_pseudogene	Gm48786	0.04772512	1.89251486
miRNA	Mir6538	0.04777342	1.53696966
unprocessed_pseudogene	Gm14086	0.04822794	1.7272813
polymorphic_pseudogene	Gm15922	0.04822794	1.5182899
lncRNA	Gm11266	0.04837565	1.70561995
processed_pseudogene	Gm47842	0.04857392	2.59396755
lncRNA	A530001N23Rik	0.04872238	2.47025884
TEC	Gm44669	0.04891781	1.63358089
lncRNA	A530010L16Rik	0.04899883	1.7251883
protein_coding	Mrgpra2a	0.0492087	1.76704017
protein_coding	Kcnc3	0.04927397	1.51334851
lncRNA	4933401D09Rik	0.04937331	2.91445433
miRNA	Mir143	0.04973767	2.21066708
protein_coding	Mypn	0.0498771	1.74388401

2.2. Statistically significant (FDR adj p≤0.05) downregulated (≥1.5 fold genes)

**Feature_gene_biotype**	**Feature_gene_name**	**padj**	**FoldChange**
protein_coding	Col3a1	1.91E-28	0.155777902
protein_coding	Col6a3	3.49E-28	0.36196761
lncRNA	Gm36827	6.68E-28	0.210485879
protein_coding	A2m	6.15E-26	0.038239371
protein_coding	Prss35	1.31E-25	0.124069502
protein_coding	Loxl2	1.21E-24	0.192199159
lncRNA	Gnasas1	4.73E-22	0.162851181
protein_coding	Col12a1	9.42E-22	0.180769184
protein_coding	Nrk	1.52E-21	0.121733622
protein_coding	Lrrc15	1.49E-20	0.175207352
protein_coding	P4ha3	1.48E-18	0.246240398
protein_coding	Plekhg4	1.82E-18	0.273695878
protein_coding	Syt14	6.94E-18	0.217348356
protein_coding	Myo16	1.66E-17	0.256765467
TEC	Gm37403	9.45E-17	0.26647895
protein_coding	Adam12	1.13E-16	0.398799263
protein_coding	Serinc5	4.97E-16	0.530148244
protein_coding	C1qtnf3	7.52E-16	0.107023543
protein_coding	Col6a1	2.84E-15	0.346987741
protein_coding	Arl5c	7.01E-15	0.487981516
protein_coding	Fbn2	1.03E-14	0.264633305

© 2023. Nandy A et al. Published online at http://www.aginganddisease.org/EN/10.14336/AD.2023.0510

protein_coding	Col16a1	1.07E-14	0.305203637
protein_coding	Itm2a	1.98E-14	0.249794466
protein_coding	Tnn	2.60E-14	0.17407271
protein_coding	Tspan18	4.18E-14	0.503784482
protein_coding	Col6a2	5.94E-14	0.372275576
protein_coding	Ptgfrn	1.34E-13	0.451569896
protein_coding	Lrrc17	1.41E-13	0.330539632
protein_coding	Postn	1.80E-13	0.211861197
protein_coding	Spib	3.09E-13	0.516445367
protein_coding	Igfbp3	5.64E-13	0.381686708
protein_coding	Col11a1	1.12E-12	0.231977321
protein_coding	Col8a1	1.19E-12	0.345737258
protein_coding	Slc12a3	1.58E-12	0.313786197
protein_coding	Capn6	2.11E-12	0.220039144
protein_coding	Mfap2	2.31E-12	0.399840648
protein_coding	Dcc	4.64E-12	0.165415253
protein_coding	Wnt7b	6.31E-12	0.11701256
protein_coding	Col5a1	8.89E-12	0.39455102
protein_coding	Il2ra	1.01E-11	0.332662141
protein_coding	Plagl1	1.75E-11	0.465508606
protein_coding	Lratd1	2.49E-11	0.235390894
protein_coding	Ccn5	3.82E-11	0.353355176
lncRNA	Gm26633	4.76E-11	0.066816795
protein_coding	Rag1	7.24E-11	0.301350557
protein_coding	Kcnj15	7.24E-11	0.314116199
protein_coding	C1qtnf6	7.57E-11	0.33643105
protein_coding	Lurap1l	9.92E-11	0.36840397
protein_coding	Matn2	1.09E-10	0.443406312
protein_coding	Adarb2	1.27E-10	0.252302828
protein_coding	Epha3	1.29E-10	0.296030622
protein_coding	Chst3	1.45E-10	0.584331526
lncRNA	Gm36595	1.65E-10	0.182670024
protein_coding	Bach2	1.67E-10	0.518441617
protein_coding	Ptn	1.82E-10	0.232847176
protein_coding	Fstl1	1.82E-10	0.502399474
protein_coding	Klhl14	2.78E-10	0.341734493
protein_coding	Cpm	2.78E-10	0.425316919
lncRNA	Gm20743	3.20E-10	0.375836074
protein_coding	Col5a2	7.43E-10	0.326212268
TEC	Gm38032	9.84E-10	0.110835817
protein_coding	Spock1	1.08E-09	0.128055553

© 2023. Nandy A et al. Published online at http://www.aginganddisease.org/EN/10.14336/AD.2023.0510

protein_coding	Dnah1	1.13E-09	0.494655367
protein_coding	Thbs2	1.31E-09	0.280868675
protein_coding	Fam167a	1.45E-09	0.399298496
protein_coding	Antxr1	1.88E-09	0.540409088
protein_coding	Gja1	1.91E-09	0.312819396
lncRNA	Gm49085	2.87E-09	0.34226819
protein_coding	Apln	2.94E-09	0.352271519
protein_coding	Lamb1	4.44E-09	0.509624097
protein_coding	Robo1	4.83E-09	0.476406891
protein_coding	Gabrb3	4.98E-09	0.194090634
TEC	Gm45745	6.23E-09	0.474238663
protein_coding	Edaradd	6.26E-09	0.457348853
protein_coding	Fkbp14	1.64E-08	0.4722059
protein_coding	Htra3	1.72E-08	0.495024706
lncRNA	4930562D21Rik	1.74E-08	0.285829613
protein_coding	Kdelr2	1.88E-08	0.553171712
protein_coding	Fmn2	2.06E-08	0.323350968
protein_coding	Acan	2.10E-08	0.1071021
protein_coding	Rag2	2.14E-08	0.299178399
protein_coding	Slc17a8	2.88E-08	0.351121302
TEC	Gm49266	2.88E-08	0.430240738
TEC	Gm37783	2.94E-08	0.483600573
protein_coding	Gpr153	2.94E-08	0.541424017
protein_coding	Fstl4	3.16E-08	0.315744512
protein_coding	Islr	3.38E-08	0.391276611
protein_coding	Col4a1	3.38E-08	0.45899559
protein_coding	Htra1	3.69E-08	0.366266399
protein_coding	Adamts3	4.01E-08	0.608306405
protein_coding	Siglecg	4.40E-08	0.660228349
protein_coding	P2rx3	4.54E-08	0.399014958
protein_coding	Angptl4	5.23E-08	0.503704593
protein_coding	Fetub	5.30E-08	0.173698509
protein_coding	Slc6a2	6.99E-08	0.369678924
protein_coding	Angptl2	8.35E-08	0.470734223
protein_coding	Cilp2	8.48E-08	0.32831566
protein_coding	Adamts2	1.17E-07	0.552772433
lncRNA	Gm16151	1.25E-07	0.448652388
protein_coding	Col15a1	2.11E-07	0.443007969
lncRNA	1700027J07Rik	2.35E-07	0.441155407
protein_coding	Lef1	2.36E-07	0.534695454
protein_coding	Adamts17	2.54E-07	0.414451505

protein_coding	Mmp2	2.55E-07	0.401460681
protein_coding	Vpreb3	2.83E-07	0.354336588
protein_coding	Dkk3	3.44E-07	0.318998305
protein_coding	Gria2	3.82E-07	0.185124872
lncRNA	Gm34095	4.94E-07	0.259110512
lncRNA	Gm37065	5.36E-07	0.443064432
protein_coding	Scara3	5.36E-07	0.481334321
protein_coding	Rapgefl1	5.60E-07	0.427736185
lncRNA	Rian	5.91E-07	0.355650825
protein_coding	Efna5	7.46E-07	0.561463621
protein_coding	Gpx7	8.60E-07	0.444951542
protein_coding	Adamts4	8.80E-07	0.343056671
protein_coding	Arl4d	1.06E-06	0.541706215
protein_coding	Col9a1	1.13E-06	0.049857432
protein_coding	Timp1	1.24E-06	0.270634859
protein_coding	Edil3	1.27E-06	0.362654367
protein_coding	Ppic	1.28E-06	0.456557415
TEC	Gm49268	1.58E-06	0.440728514
protein_coding	Pcsk5	1.72E-06	0.60365825
protein_coding	Aplp1	2.08E-06	0.4219458
protein_coding	Slc1a4	2.10E-06	0.539850192
protein_coding	Col9a2	2.11E-06	0.086359065
protein_coding	Lox	2.50E-06	0.322207256
protein_coding	Fam129c	2.56E-06	0.450786894
protein_coding	Spon2	2.71E-06	0.427135388
protein_coding	Spats2l	2.85E-06	0.504614503
protein_coding	Gxylt2	3.04E-06	0.439233188
protein_coding	Sulf1	3.21E-06	0.450642492
processed_pseudogene	Gm12057	3.30E-06	0.360005185
protein_coding	Cthrc1	3.40E-06	0.301548074
protein_coding	Il7r	4.07E-06	0.437870648
protein_coding	Dpysl3	4.15E-06	0.515334303
protein_coding	Loxl1	4.15E-06	0.591467315
lncRNA	Bach2it1	4.25E-06	0.473695352
protein_coding	Lum	4.54E-06	0.171031084
TEC	Gm19514	4.64E-06	0.458360021
protein_coding	Vstm4	4.64E-06	0.599757446
protein_coding	Crabp2	4.74E-06	0.243846459
protein_coding	C1qtnf1	4.74E-06	0.564693787
protein_coding	Pole2	5.48E-06	0.635196864
lncRNA	2010007H06Rik	5.79E-06	0.529263079

protein_coding	Chn1	5.96E-06	0.458208715
protein_coding	Fgf18	6.21E-06	0.265072882
protein_coding	Matn4	6.38E-06	0.461152152
protein_coding	Tlcd2	6.49E-06	0.46886352
protein_coding	Dpp4	7.46E-06	0.538993295
protein_coding	Srpx	7.47E-06	0.396479125
protein_coding	Cd276	7.47E-06	0.519737218
protein_coding	Fgfr2	7.99E-06	0.655479673
protein_coding	Rcn1	8.21E-06	0.561736392
protein_coding	Gm40193	8.23E-06	0.022284259
lncRNA	Gm32743	8.78E-06	0.151680533
protein_coding	Slc13a5	8.78E-06	0.387123882
protein_coding	Cnmd	9.07E-06	0.148522297
protein_coding	Dner	9.10E-06	0.243065565
protein_coding	Ndnf	9.32E-06	0.50550316
lncRNA	5830487J09Rik	9.80E-06	0.566894748
protein_coding	Susd5	1.19E-05	0.396890451
protein_coding	Sparc	1.21E-05	0.345593721
protein_coding	Thbs3	1.26E-05	0.525024306
protein_coding	Lipc	1.29E-05	0.402365866
protein_coding	Mettl21e	1.31E-05	0.232446428
protein_coding	Fras1	1.31E-05	0.531522148
protein_coding	Tifa	1.31E-05	0.583882476
protein_coding	Olfml2b	1.43E-05	0.481571912
protein_coding	Gli3	1.43E-05	0.565053638
protein_coding	Fbxl7	1.44E-05	0.598635423
protein_coding	Zfp711	1.46E-05	0.354015097
protein_coding	Luzp2	1.50E-05	0.265977899
protein_coding	Serpinf1	1.56E-05	0.335013467
protein_coding	St8sia2	1.57E-05	0.288373211
lncRNA	4932413F04Rik	1.60E-05	0.147019688
protein_coding	Hapln1	1.61E-05	0.08788382
protein_coding	Col8a2	1.69E-05	0.447230096
protein_coding	Kdelr3	1.78E-05	0.435607069
protein_coding	Bcl7a	1.81E-05	0.619718156
protein_coding	Grb10	1.85E-05	0.553865061
protein_coding	Zfp469	1.85E-05	0.591750557
lncRNA	Bach2os	1.87E-05	0.403570111
protein_coding	Fscn1	1.87E-05	0.583555623
protein_coding	Chst10	1.98E-05	0.484390274
protein_coding	Aebp1	1.99E-05	0.559911861

© 2023. Nandy A et al. Published online at http://www.aginganddisease.org/EN/10.14336/AD.2023.0510

protein_coding	Cercam	2.00E-05	0.643844527
protein_coding	Unc5c	2.01E-05	0.338067593
protein_coding	Armcx2	2.05E-05	0.628316898
protein_coding	Chpf	2.11E-05	0.579729818
protein_coding	Serpinh1	2.18E-05	0.388196923
lncRNA	Gm32017	2.29E-05	0.400860476
protein_coding	Cecr2	2.31E-05	0.481548319
protein_coding	Col24a1	2.55E-05	0.566974421
protein_coding	Igll1	2.69E-05	0.431463487
TEC	Gm42449	2.72E-05	0.398766538
protein_coding	Ephb2	2.84E-05	0.559543654
protein_coding	Col1a2	2.93E-05	0.407289648
TEC	Gm37649	3.02E-05	0.24193215
protein_coding	Sox11	3.12E-05	0.1386813
snRNA	Gm23458	3.43E-05	0.448109136
protein_coding	Slc35g1	3.43E-05	0.630159527
protein_coding	Tmem108	3.50E-05	0.39837387
transcribed_processed_pseudogene	Gm6330	3.61E-05	0.55659812
protein_coding	Pamr1	3.67E-05	0.537746343
protein_coding	Gabra3	3.71E-05	0.284363824
protein_coding	Cgref1	3.84E-05	0.371109144
protein_coding	Alpl	3.84E-05	0.44456637
protein_coding	Nrep	4.13E-05	0.454194414
protein_coding	Frzb	4.14E-05	0.425431688
protein_coding	Mmp14	4.21E-05	0.510594067
protein_coding	Itga11	4.62E-05	0.571532646
lncRNA	Gm14120	4.91E-05	0.335918361
protein_coding	Chil4	4.95E-05	0.169841415
protein_coding	Bcat1	5.10E-05	0.539507267
processed_pseudogene	Gm9728	5.18E-05	0.186820389
protein_coding	Igf1	5.48E-05	0.597759468
protein_coding	Peg3	5.60E-05	0.477976663
protein_coding	Mme	5.68E-05	0.497616144
protein_coding	Bcan	5.94E-05	0.348964443
protein_coding	Fcrla	6.04E-05	0.518418041
protein_coding	Pcolce	6.50E-05	0.534303986
protein_coding	Arfgef3	6.52E-05	0.427281282
protein_coding	Cd72	6.52E-05	0.585476014
protein_coding	Timp2	6.82E-05	0.537404304
protein_coding	Fcrls	7.06E-05	0.377237307
protein_coding	Mpzl1	7.18E-05	0.61010271

© 2023. Nandy A et al. Published online at http://www.aginganddisease.org/EN/10.14336/AD.2023.0510

protein_coding	Igsf10	7.21E-05	0.411733658
protein_coding	Slc39a8	7.46E-05	0.590437527
protein_coding	Clmp	8.09E-05	0.597206021
protein_coding	Cplx2	8.29E-05	0.500934581
protein_coding	Egfl6	8.38E-05	0.317600617
protein_coding	Smpd3	9.13E-05	0.487256028
lncRNA	Gm16316	9.39E-05	0.568788879
protein_coding	Fkbp7	9.73E-05	0.411686469
protein_coding	P3h1	9.83E-05	0.613669813
processed_pseudogene	Gm6478	9.93E-05	0.497374838
TEC	Gm43313	0.000102	0.597227862
TEC	Gm43910	0.000105	0.624872694
protein_coding	Col1a1	0.00011	0.429890106
lncRNA	B830012L14Rik	0.000111	0.419767592
protein_coding	Mmp23	0.000111	0.625996278
protein_coding	Zfp354c	0.000113	0.537450155
lncRNA	Gm47730	0.000114	0.382360094
protein_coding	Trim2	0.000114	0.62551784
protein_coding	Mfap4	0.000118	0.338859411
protein_coding	Ccdc80	0.000125	0.544876379
protein_coding	Wif1	0.000128	0.412384023
protein_coding	S1pr1	0.000128	0.643218339
protein_coding	Lonrf2	0.000133	0.414128986
TEC	AC115752.1	0.000136	0.521896188
protein_coding	AI593442	0.000138	0.288326576
lncRNA	D530037P16Rik	0.000138	0.356649411
protein_coding	Fkbp10	0.000138	0.508902627
protein_coding	Pipox	0.000144	0.382589345
lncRNA	Redrum	0.000152	0.527065517
protein_coding	BC035044	0.000155	0.593735507
protein_coding	Slc10a4	0.000156	0.264075133
protein_coding	Cd79b	0.00017	0.632623775
protein_coding	Steap2	0.000173	0.606265977
protein_coding	Scrn1	0.000174	0.468436592
lncRNA	1810059H22Rik	0.00018	0.439791911
lncRNA	Meg3	0.00018	0.519831395
protein_coding	Col4a2	0.00018	0.559218017
TEC	Gm43428	0.000183	0.321480722
TEC	Gm37527	0.000185	0.51403593
processed_pseudogene	Gm5915	0.000186	0.454529099
protein_coding	Rims1	0.000187	0.311017525

SUPPLEMENTARY DATA

protein_coding	Abitram	0.000207	0.660559521
protein_coding	Pafah1b3	0.00021	0.545038
protein_coding	Gask1a	0.000214	0.505802688
snoRNA	Gm26448	0.000216	0.441606207
protein_coding	Col5a3	0.000219	0.533497229
TEC	Gm48261	0.000221	0.20053083
protein_coding	Tram1l1	0.000232	0.079836705
protein_coding	Gpm6b	0.000237	0.66017467
protein_coding	Bglap2	0.000238	0.255919668
protein_coding	Pycr1	0.000241	0.591143244
protein_coding	Megf6	0.000245	0.444483494
protein_coding	Csrnp3	0.000245	0.464732709
lncRNA	Gm26577	0.000245	0.503846235
lncRNA	2310008N11Rik	0.000251	0.320836958
protein_coding	Tmem45a	0.000253	0.41982443
protein_coding	Crtap	0.000261	0.64532904
protein_coding	Ptk7	0.000263	0.627768572
lncRNA	Gm35024	0.000273	0.035439818
protein_coding	Capsl	0.000274	0.285547655
protein_coding	Flrt2	0.000283	0.480744595
protein_coding	Sox4	0.00029	0.600857856
protein_coding	Cnnm1	0.000295	0.591221105
protein_coding	11-Mar	0.000325	0.192813647
protein_coding	Olfml3	0.00033	0.51713221
TEC	Gm43961	0.00034	0.587170321
protein_coding	Nell1	0.000341	0.195264129
protein_coding	Slc2a10	0.000391	0.630691442
protein_coding	Actc1	0.000401	0.216097526
lncRNA	Gm49838	0.000401	0.406940561
protein_coding	B3gnt9	0.000401	0.606343834
snoRNA	Gm24336	0.000408	0.467201939
protein_coding	Sertad4	0.000425	0.591155768
TEC	Gm49839	0.000432	0.405738952
lncRNA	Gm42982	0.000436	0.516409915
protein_coding	Pou2af1	0.000457	0.586398415
misc_RNA	Rny3	0.000471	0.324355918
protein_coding	Cdh2	0.000471	0.522662946
protein_coding	Sh3pxd2b	0.000471	0.584715456
protein_coding	Il1rl1	0.00048	0.590560868
protein_coding	Fam149a	0.000481	0.65681559
protein_coding	Dusp5	0.000481	0.610752902

© 2023. Nandy A et al. Published online at http://www.aginganddisease.org/EN/10.14336/AD.2023.0510

processed_pseudogene	Gm34727	0.000492	0.417721403
protein_coding	Cdh11	0.000498	0.562343724
protein_coding	Aplnr	0.000524	0.503595911
protein_coding	Pdgfrl	0.000536	0.410001831
protein_coding	Reps2	0.000536	0.591769874
lncRNA	1810053B23Rik	0.000562	0.327427869
protein_coding	Cpxm1	0.000565	0.505316399
processed_pseudogene	Gm2962	0.00058	0.43738681
protein_coding	Tmem119	0.000588	0.573017047
protein_coding	Adamts12	0.000625	0.632956396
protein_coding	Suv39h2	0.000638	0.654408771
protein_coding	Epha4	0.000653	0.650567396
protein_coding	Map3k21	0.000659	0.351548903
protein_coding	Nup35	0.000663	0.621150348
processed_pseudogene	Gm8848	0.000665	0.366305069
protein_coding	Id3	0.000669	0.66021299
protein_coding	Sdr9c7	0.000673	0.058971048
protein_coding	Serpine1	0.000703	0.469894251
lncRNA	Gm21986	0.000706	0.450729139
snRNA	Rnu12	0.000707	0.411608417
protein_coding	Cemip	0.000728	0.454353305
protein_coding	Maged1	0.000731	0.623041708
protein_coding	Rfc4	0.000747	0.644566494
protein_coding	Ercc6l	0.000778	0.641390541
protein_coding	Sdc1	0.000791	0.594032013
protein_coding	Kcnk1	0.000833	0.51386053
protein_coding	Minpp1	0.000858	0.578921583
protein_coding	Cdo1	0.000858	0.513646973
TEC	Gm38036	0.000873	0.587225702
protein_coding	Emid1	0.000877	0.647260783
protein_coding	Sdc2	0.000888	0.547317194
protein_coding	Kazald1	0.000906	0.537965789
protein_coding	Twist1	0.000908	0.560979175
protein_coding	D630045J12Rik	0.000924	0.634342932
lncRNA	Gm12158	0.00094	0.525599745
misc_RNA	Gm24371	0.000997	0.386004331
protein_coding	Nsg1	0.001007	0.580790825
lncRNA	4930478L05Rik	0.001021	0.584894655
protein_coding	Ddit4	0.001043	0.476489554
protein_coding	Stc1	0.001097	0.486507984
protein_coding	Fbn1	0.001121	0.636676842

© 2023. Nandy A et al. Published online at http://www.aginganddisease.org/EN/10.14336/AD.2023.0510

protein_coding	Dchs1	0.001155	0.574546971
protein_coding	Rpl39l	0.001192	0.322742671
protein_coding	Sema3d	0.001192	0.461031475
protein_coding	Frmd3	0.001254	0.47545595
protein_coding	Tmem98	0.001269	0.586562468
protein_coding	P3h4	0.001276	0.578993661
lncRNA	9530034A14Rik	0.001284	0.480955313
protein_coding	Mdk	0.001378	0.622345646
TEC	C730045M19Rik	0.001378	0.65833404
protein_coding	Bub1	0.001423	0.658349059
protein_coding	Lrrc18	0.001433	0.568402942
lncRNA	Gm47828	0.00144	0.559083917
snRNA	Gm26465	0.001483	0.411086627
protein_coding	Slc16a4	0.001491	0.604985722
protein_coding	Wnt5a	0.001491	0.628165768
transcribed_unprocessed_pseudogene	Zfp125	0.001509	0.533024998
TEC	Gm43482	0.001518	0.637157378
protein_coding	Sec24d	0.001545	0.614976321
processed_pseudogene	Gm8774	0.001551	0.591096288
protein_coding	Lrrc55	0.001626	0.317083752
protein_coding	Ccn3	0.001628	0.263404167
protein_coding	Aunip	0.001667	0.64486049
protein_coding	Ror2	0.001692	0.56411075
processed_pseudogene	Gm11805	0.001698	0.623983963
protein_coding	Kctd4	0.001704	0.601757719
processed_pseudogene	Gm5470	0.001707	0.280574836
TEC	Gm37674	0.001763	0.489219129
protein_coding	Zkscan16	0.001826	0.540408411
protein_coding	Dcbld1	0.001828	0.657867678
protein_coding	Ibsp	0.001878	0.397603393
TEC	Gm48014	0.001879	0.355252433
protein_coding	Gulp1	0.001881	0.570852903
protein_coding	Cybrd1	0.001895	0.396192078
miRNA	Mir144	0.001895	0.498725992
protein_coding	Flrt3	0.001907	0.569501411
protein_coding	Elovl6	0.00197	0.656791641
protein_coding	Arhgef26	0.001972	0.633063743
protein_coding	Fn1	0.001975	0.558748239
processed_pseudogene	Gm19144	0.001976	0.638418557
protein_coding	Kcns1	0.001991	0.389026837
protein_coding	Mex3a	0.001991	0.598324087

© 2023. Nandy A et al. Published online at http://www.aginganddisease.org/EN/10.14336/AD.2023.0510

protein_coding	Fat3	0.002009	0.65033297
protein_coding	Fzd3	0.002012	0.583883377
lncRNA	Gm47357	0.002014	0.514872427
protein_coding	Zfp953	0.002014	0.629865804
protein_coding	Podnl1	0.002024	0.598213943
TEC	Gm38140	0.002033	0.409226951
TEC	Gm43921	0.002152	0.511148842
protein_coding	Smoc2	0.002225	0.467769122
lncRNA	Gm43462	0.002322	0.653995636
protein_coding	Camk4	0.002347	0.617110953
protein_coding	Tmem132a	0.002353	0.589911996
protein_coding	Natd1	0.002381	0.658030779
protein_coding	Bglap	0.002419	0.260881227
protein_coding	Praf2	0.00243	0.505709536
processed_pseudogene	Gm50425	0.002457	0.542848913
lncRNA	Gm12503	0.002464	0.653711317
protein_coding	Clec11a	0.002499	0.491207868
TEC	Gm42549	0.002566	0.637106459
protein_coding	H3c15	0.002582	0.417880482
protein_coding	Sstr2	0.002659	0.476399847
protein_coding	Dipk1c	0.002674	0.535845796
protein_coding	Tbx4	0.002738	0.500512791
snRNA	Rnu5g	0.002763	0.21935879
TEC	Gm37285	0.002767	0.627950779
protein_coding	Slc30a10	0.002885	0.547501504
protein_coding	Kera	0.002896	0.422614528
protein_coding	Mboat2	0.002897	0.600729159
protein_coding	Dntt	0.003055	0.603919068
protein_coding	Hey1	0.003086	0.62260258
TEC	Gm47337	0.003096	0.05217622
protein_coding	Pbk	0.003096	0.609506517
protein_coding	Zim1	0.0031	0.517699195
protein_coding	Entpd3	0.003257	0.545681866
protein_coding	1700066M21Rik	0.003289	0.63803065
protein_coding	Tnfaip6	0.003343	0.512970167
TEC	Gm42909	0.003359	0.423615095
protein_coding	Igfbp2	0.003376	0.299246388
protein_coding	Hmcn1	0.003392	0.643924933
protein_coding	Gm8251	0.003518	0.52562216
protein_coding	Tmem47	0.003548	0.577762924
protein_coding	Wdr72	0.003569	0.305340446

© 2023. Nandy A et al. Published online at http://www.aginganddisease.org/EN/10.14336/AD.2023.0510

protein_coding	Rnf182	0.003609	0.411254014
protein_coding	Arf4	0.003609	0.621977088
protein_coding	Cd209f	0.003637	0.340779082
protein_coding	Zfp808	0.003637	0.545271097
protein_coding	S1pr3	0.003706	0.624998497
protein_coding	Ccn4	0.003763	0.559449916
polymorphic_pseudogene	Pla2g2a	0.003776	0.044779906
protein_coding	Fbxo48	0.003794	0.658813944
protein_coding	Tnfsf11	0.003831	0.488375273
protein_coding	Ism1	0.003831	0.631898635
snoRNA	Gm25394	0.00387	0.517403415
protein_coding	Eef1akmt4	0.003966	0.543279503
lncRNA	Gm44993	0.003993	0.403658106
protein_coding	Pax1	0.004068	0.289614867
protein_coding	Sfrp4	0.004068	0.620678064
protein_coding	Aldh1l2	0.004097	0.621503827
lncRNA	Gm17120	0.004132	0.297492154
protein_coding	Tmem132e	0.004229	0.635845989
TEC	4930517J16Rik	0.004281	0.6291751
TEC	Gm43504	0.004425	0.548196866
lncRNA	4930426D05Rik	0.004473	0.582966299
protein_coding	Slc26a7	0.004478	0.598527662
protein_coding	Cnn3	0.004599	0.537593765
protein_coding	Pklr	0.004658	0.625037073
protein_coding	Htra4	0.004703	0.457567269
protein_coding	Zfp760	0.004703	0.640308328
transcribed_processed_pseudogene	Gm7240	0.004757	0.54679817
protein_coding	Chad	0.004874	0.438955534
protein_coding	Akap12	0.004876	0.625484696
misc_RNA	Rny1	0.004883	0.441855161
protein_coding	Dnajb13	0.004921	0.630307175
protein_coding	Ammecr1	0.004935	0.658377916
protein_coding	Ankrd29	0.004938	0.552680738
protein_coding	Pnmal2	0.00494	0.529387897
protein_coding	Pnp	0.004955	0.642296735
protein_coding	Islr2	0.004961	0.545148208
protein_coding	Gm16867	0.005011	0.571787403
scaRNA	Gm24289	0.005038	0.45222267
protein_coding	Fap	0.005041	0.541318713
protein_coding	Rtl1	0.005073	0.450218842
protein_coding	Tenm3	0.005232	0.552232352

© 2023. Nandy A et al. Published online at http://www.aginganddisease.org/EN/10.14336/AD.2023.0510

protein_coding	Nt5dc2	0.005251	0.646008078
TEC	Gm42852	0.005334	0.48194614
protein_coding	Nnmt	0.005338	0.637621213
protein_coding	Mmp13	0.005352	0.308556436
protein_coding	Zfp976	0.005352	0.639481523
protein_coding	Fndc4	0.005393	0.641329778
processed_pseudogene	Gm49760	0.005413	0.459567601
protein_coding	C1qtnf12	0.005825	0.65187052
TEC	Gm43864	0.005829	0.606446737
TEC	Gm43508	0.005831	0.639398722
protein_coding	Gm21680	0.006066	0.386193939
protein_coding	Brinp1	0.006211	0.242909662
transcribed_processed_pseudogene	Gm29036	0.006221	0.603111292
misc_RNA	Gm27899	0.006253	0.072409084
protein_coding	Col26a1	0.006279	0.427886668
processed_pseudogene	Gm13510	0.00632	0.501383682
TEC	Gm36638	0.006321	0.607215548
lncRNA	Snhg18	0.006332	0.550907588
processed_pseudogene	Gm8428	0.006556	0.584309331
protein_coding	Mxra8	0.006571	0.657001061
protein_coding	Sh3pxd2a	0.006571	0.657299691
TEC	Gm50245	0.006576	0.504500277
protein_coding	Kcnh1	0.006576	0.57663885
unprocessed_pseudogene	Gm5834	0.006601	0.437030476
protein_coding	Serf1	0.006675	0.447219303
protein_coding	Chst8	0.006869	0.456359544
lncRNA	Gm44089	0.006978	0.568740812
protein_coding	Plod2	0.007073	0.499926656
protein_coding	Hacd1	0.007114	0.589275485
protein_coding	Slitrk6	0.007158	0.422880221
protein_coding	Lpar3	0.007307	0.604427503
processed_pseudogene	Gm42578	0.007312	0.373300357
protein_coding	Gria1	0.007315	0.119382336
protein_coding	Lrch2	0.007349	0.462682261
lncRNA	Gm36988	0.007404	0.504525846
protein_coding	C6	0.007408	0.396182673
TEC	Gm45819	0.007436	0.627493916
lncRNA	4833422M21Rik	0.00746	0.490635131
TEC	Gm44415	0.00746	0.522712936
TEC	Gm44078	0.007496	0.540924067
protein_coding	Sgms2	0.007498	0.606553002

© 2023. Nandy A et al. Published online at http://www.aginganddisease.org/EN/10.14336/AD.2023.0510

protein_coding	Col10a1	0.007624	0.290841053
protein_coding	Bambi	0.007624	0.545751361
TEC	Gm44416	0.007624	0.607191984
protein_coding	Vpreb1	0.00775	0.510235544
processed_pseudogene	Gm8828	0.007812	0.389717993
processed_pseudogene	Gm6789	0.007838	0.483548071
protein_coding	Slc10a6	0.007858	0.499342544
unprocessed_pseudogene	Gm21451	0.008018	0.444368808
protein_coding	Car2	0.008158	0.565417822
protein_coding	Mro	0.008244	0.574279112
protein_coding	Emp1	0.008325	0.614458377
protein_coding	Csrp2	0.008325	0.636051228
protein_coding	Cpz	0.008328	0.271570717
unprocessed_pseudogene	Gm18665	0.008394	0.317271775
protein_coding	Adamtsl1	0.008436	0.65912204
protein_coding	Nmnat2	0.00846	0.546071225
TEC	Gm48308	0.00851	0.503058098
protein_coding	Nupr1	0.008528	0.577648134
lncRNA	Gm15774	0.008534	0.640590395
processed_pseudogene	Gm11447	0.00871	0.48948735
protein_coding	Efcab1	0.008797	0.235269293
snRNA	Gm24019	0.008886	0.213987248
protein_coding	Hapln4	0.008908	0.486339905
protein_coding	Mrgprf	0.009046	0.496632445
protein_coding	Ccna2	0.009056	0.610464667
protein_coding	Ccn1	0.009226	0.572912876
TEC	Gm45508	0.009346	0.48064198
TEC	Gm42648	0.009346	0.644435246
TEC	Gm49265	0.009497	0.545859884
protein_coding	Ccne2	0.009585	0.56099314
protein_coding	Tmem263	0.009586	0.616464563
protein_coding	Glt8d2	0.00959	0.60120291
protein_coding	Tll1	0.009603	0.553122687
lncRNA	Gm22146	0.009635	0.431600128
processed_pseudogene	Gm45628	0.009746	0.461218835
TEC	Gm48015	0.009785	0.464710184
lncRNA	4833422C13Rik	0.009952	0.657269309
miRNA	Gm24302	0.010005	0.461871275
protein_coding	Mamdc2	0.010018	0.645728462
unprocessed_pseudogene	Gm49331	0.010144	0.563155822
snRNA	Gm25713	0.010261	0.376126155

protein_coding	Ccdc92b	0.010347	0.63146856
lncRNA	Gm49083	0.01057	0.05504723
lncRNA	A930006K02Rik	0.01057	0.637835067
protein_coding	Cldn10	0.010669	0.489041782
protein_coding	Plppr4	0.010848	0.352176796
protein_coding	Enpp3	0.010848	0.582962112
protein_coding	Nxpe2	0.011125	0.612593814
protein_coding	Phex	0.01137	0.576268834
lncRNA	Gm30211	0.011426	0.629222629
protein_coding	Fat4	0.011587	0.645926314
protein_coding	Galr2	0.011648	0.652787035
TEC	Gm38211	0.01169	0.498986727
protein_coding	Tspan6	0.01169	0.567801335
protein_coding	Omd	0.011698	0.355810414
processed_pseudogene	Gm14834	0.011698	0.417220216
lncRNA	Gm16110	0.011698	0.448295298
protein_coding	Lman1l	0.011907	0.517802482
protein_coding	Pgbd5	0.012025	0.613785765
TEC	Gm37606	0.012119	0.591086969
protein_coding	Vstm2b	0.012167	0.348377546
protein_coding	Vcan	0.012167	0.592294123
TEC	Gm29438	0.012167	0.651750914
lncRNA	4933400C23Rik	0.012291	0.157764025
protein_coding	Cubn	0.012409	0.569035361
protein_coding	Arc	0.012557	0.592950107
processed_pseudogene	Gm18329	0.012722	0.413634722
protein_coding	Pdpn	0.012963	0.48491978
protein_coding	Hs3st3a1	0.013033	0.569970891
processed_pseudogene	Gm18163	0.013097	0.069163131
lncRNA	Gm26652	0.013451	0.552609502
protein_coding	Fam180a	0.013527	0.463692356
protein_coding	Ccdc3	0.013527	0.584239047
lncRNA	Mirg	0.013675	0.489758739
lncRNA	Gm16248	0.013826	0.382134072
lncRNA	Gm12743	0.013869	0.65984099
protein_coding	Fibin	0.014168	0.601038272
protein_coding	Pwwp3b	0.014202	0.618517376
protein_coding	Kif18a	0.014251	0.61228486
TEC	Gm37534	0.0146	0.597314589
TEC	Gm43130	0.014754	0.470932566
protein_coding	Gm14410	0.01488	0.638213052

© 2023. Nandy A et al. Published online at http://www.aginganddisease.org/EN/10.14336/AD.2023.0510

SUPPLEMENTARY DATA

lncRNA	Fbxl12os	0.015267	0.624904785
lncRNA	Gm48960	0.015363	0.56659807
lncRNA	1700071G01Rik	0.015462	0.561766889
protein_coding	Col11a2	0.015462	0.581907911
lncRNA	Gm43138	0.015462	0.633688585
protein_coding	Chl1	0.015462	0.655446485
TEC	Gm37899	0.015549	0.459956473
protein_coding	Slc46a1	0.015689	0.618463442
unprocessed_pseudogene	Gm7897	0.015822	0.455228473
processed_pseudogene	Gm36298	0.015822	0.547399546
TEC	Gm45240	0.01597	0.648857508
protein_coding	Dcn	0.016147	0.481556857
protein_coding	Plat	0.016185	0.493761056
lncRNA	C130021I20Rik	0.016241	0.569059614
processed_pseudogene	Gm7219	0.016542	0.069983234
processed_pseudogene	Gm11688	0.01681	0.479603829
protein_coding	Pxdn	0.016827	0.649771914
TEC	Gm37298	0.016884	0.575813892
protein_coding	Nags	0.016991	0.430136895
lncRNA	2410018L13Rik	0.017042	0.190184112
protein_coding	Tcaf2	0.017234	0.637198131
protein_coding	Frk	0.01748	0.646552909
protein_coding	Sdk2	0.017482	0.658797208
protein_coding	Krt75	0.017594	0.313767145
protein_coding	Snai2	0.017785	0.553981403
lncRNA	Gm15418	0.017994	0.238163155
processed_pseudogene	Gm50258	0.018075	0.214454775
lncRNA	A230056P14Rik	0.018075	0.608939338
processed_pseudogene	Gm15833	0.018245	0.387881002
lncRNA	Gm26930	0.018308	0.613089058
protein_coding	Stag3	0.018568	0.640696822
protein_coding	Camk1g	0.018651	0.387171674
protein_coding	Bdh2	0.018895	0.529521325
lncRNA	Gm41724	0.019043	0.39936604
snRNA	Gm25939	0.019312	0.56303197
protein_coding	Dsel	0.019338	0.626684975
miRNA	Mir1188	0.019463	0.128440376
lncRNA	Gm19434	0.019465	0.609430876
protein_coding	Snorc	0.019607	0.297891203
processed_pseudogene	Gm9570	0.019844	0.647682999
lncRNA	Gm44238	0.019939	0.59669471

© 2023. Nandy A et al. Published online at http://www.aginganddisease.org/EN/10.14336/AD.2023.0510

unprocessed_pseudogene	Cyp4b1-ps2	0.019953	0.551293526
protein_coding	Col14a1	0.019953	0.627105651
TEC	Gm47258	0.02003	0.631065092
TEC	Gm42929	0.020252	0.590376404
protein_coding	Nectin3	0.020469	0.654688895
processed_pseudogene	Gm12926	0.020477	0.65832306
processed_pseudogene	Gm19045	0.020738	0.554793461
lncRNA	Gm43149	0.020738	0.639551266
protein_coding	Matn3	0.020809	0.127842084
processed_pseudogene	Gm38266	0.020809	0.33940808
lncRNA	Gm10578	0.02082	0.642441875
protein_coding	Bgn	0.020869	0.575108803
processed_pseudogene	Gm8523	0.02104	0.598365843
miRNA	Gm22117	0.021205	0.49894924
protein_coding	Rsph1	0.021205	0.512926079
protein_coding	Crispld1	0.021297	0.535064174
protein_coding	Prrx2	0.021378	0.567204906
protein_coding	Rtl3	0.02209	0.500057599
protein_coding	Esco2	0.022415	0.637094772
processed_pseudogene	Gm46649	0.022516	0.504007898
TEC	Gm48203	0.022634	0.468470085
protein_coding	Srd5a1	0.022678	0.53934851
protein_coding	Gabra5	0.022972	0.121104999
protein_coding	Oip5	0.023138	0.659422026
lncRNA	Gm43259	0.023212	0.639945936
lncRNA	Gm13381	0.023675	0.524383918
protein_coding	Rhag	0.023908	0.598968278
protein_coding	Colec12	0.023908	0.602607003
protein_coding	Wasf1	0.024218	0.610192739
protein_coding	Syt9	0.024218	0.634194514
protein_coding	AU015836	0.024376	0.344599743
TEC	Gm43268	0.024439	0.63119154
protein_coding	Ccdc68	0.024953	0.592414107
protein_coding	Ssu2	0.024959	0.460691094
snoRNA	Gm22422	0.025088	0.457291794
miRNA	Mir540	0.025132	0.126552447
TEC	Gm48562	0.025176	0.63231435
TEC	Gm43339	0.02522	0.649042886
protein_coding	Cd109	0.025288	0.547353723
protein_coding	Lrrc4b	0.025288	0.583270338
TEC	Gm46436	0.025337	0.614324043

© 2023. Nandy A et al. Published online at http://www.aginganddisease.org/EN/10.14336/AD.2023.0510

lncRNA	F930017D23Rik	0.025372	0.658877137
TEC	Gm43429	0.025372	0.457055902
protein_coding	Prss23	0.02598	0.541879672
protein_coding	Prss46	0.026529	0.41060181
protein_coding	Trim59	0.026737	0.656789504
protein_coding	Rnf112	0.026814	0.285811629
lncRNA	Gm16096	0.026883	0.493697075
protein_coding	Cfap47	0.026915	0.482749162
protein_coding	Gnai1	0.026988	0.637148562
lncRNA	4833417C18Rik	0.027245	0.634985842
lncRNA	Gm27042	0.027704	0.54961414
TEC	Gm38235	0.027704	0.654732044
lncRNA	Gm28379	0.027992	0.637481042
processed_pseudogene	Gm48260	0.028218	0.307716463
transcribed_processed_pseudogene	Gm18194	0.028218	0.529884563
protein_coding	Rtn4r	0.028218	0.629407755
lncRNA	Gm42997	0.028357	0.253222377
protein_coding	Tssk3	0.028357	0.513411339
protein_coding	Pcdh20	0.028754	0.187574598
protein_coding	Rnf207	0.028775	0.598638596
lncRNA	4930588J15Rik	0.029036	0.427598503
protein_coding	Foxa2	0.029057	0.136316851
protein_coding	Mcpt4	0.0291	0.284076981
lncRNA	Gm15344	0.02916	0.628939126
protein_coding	Pitx1	0.029162	0.569116341
TEC	2810428J06Rik	0.029288	0.637385463
snRNA	Gm26446	0.030045	0.227214473
protein_coding	Mlf1	0.030055	0.522975704
protein_coding	Rftn2	0.030055	0.605313789
lncRNA	Gm43221	0.030081	0.619712442
protein_coding	Olfr164	0.03071	0.604678567
lncRNA	Gm10441	0.030753	0.144892164
lncRNA	Gm43256	0.030861	0.650682202
processed_pseudogene	Gm17808	0.031184	0.550853291
lncRNA	Gm34574	0.031577	0.435520537
processed_pseudogene	Gm43967	0.031593	0.498744595
protein_coding	Abcc6	0.031596	0.384137026
lncRNA	Gm16549	0.031835	0.394878045
protein_coding	Cldn13	0.031938	0.656759361
lncRNA	C130073E24Rik	0.032002	0.319244683
TEC	Gm43054	0.032365	0.647163286

© 2023. Nandy A et al. Published online at http://www.aginganddisease.org/EN/10.14336/AD.2023.0510

TEC	Gm48442	0.032449	0.519304598
ribozyme	Gm22714	0.03252	0.216314199
miRNA	Mir6392	0.032619	0.166039747
protein_coding	Tcfl5	0.032687	0.397018424
protein_coding	Tmem35a	0.032717	0.398202821
protein_coding	Znrd2	0.032871	0.52852417
protein_coding	Syndig1l	0.032928	0.389728111
protein_coding	Ddias	0.033234	0.657028514
TEC	Gm46339	0.033333	0.369008503
lncRNA	4933431G14Rik	0.033848	0.620518146
snoRNA	Gm25788	0.033949	0.567000612
processed_pseudogene	Gm16269	0.034187	0.3782933
protein_coding	Mageh1	0.034334	0.638018231
protein_coding	Scn1a	0.034865	0.463991452
TEC	Gm38083	0.034865	0.59790179
protein_coding	Klhl13	0.034865	0.6413802
processed_pseudogene	Gm45344	0.035019	0.444083305
protein_coding	Slc6a20a	0.035119	0.66028697
protein_coding	Rcn3	0.035579	0.583947251
TEC	2900035J10Rik	0.03575	0.460364435
protein_coding	Zfp456	0.03578	0.594769788
protein_coding	Gm3604	0.035838	0.550973565
lncRNA	Mir181a-1hg	0.035838	0.55805842
protein_coding	Cfhr2	0.035869	0.407164599
lncRNA	1700017G19Rik	0.036342	0.644693757
protein_coding	Smarca1	0.036389	0.647698922
protein_coding	Nusap1	0.036399	0.640143346
TEC	Gm42983	0.036498	0.643483632
lncRNA	Gm17494	0.036681	0.641652803
protein_coding	Zfp874a	0.03674	0.646454924
protein_coding	Fbln1	0.036825	0.627685343
protein_coding	Serpinb6b	0.036901	0.615978858
processed_pseudogene	Gm18811	0.037034	0.545618778
protein_coding	Ifi209	0.037129	0.634388032
miRNA	Gm26301	0.037485	0.481323274
TEC	Gm49132	0.037723	0.561217967
lncRNA	Gm49024	0.038568	0.573789378
lncRNA	Gm49518	0.039066	0.347388207
protein_coding	Tspyl5	0.039076	0.61697342
protein_coding	Tceal3	0.039183	0.544380384
lncRNA	Gm11630	0.039847	0.327989814

© 2023. Nandy A et al. Published online at http://www.aginganddisease.org/EN/10.14336/AD.2023.0510

lncRNA	Gm45359	0.039847	0.498084194
protein_coding	Npy4r	0.040073	0.22637585
lncRNA	Gm16725	0.040073	0.605474374
lncRNA	Gm15849	0.040523	0.608848846
protein_coding	Gpr22	0.040523	0.629060978
miRNA	Mir703	0.040544	0.483829519
snoRNA	AF357399	0.040685	0.384040215
processed_pseudogene	Gm18388	0.040846	0.602633862
miRNA	Gm44458	0.040846	0.603944914
protein_coding	Gm266	0.041029	0.631810772
protein_coding	P2ry10b	0.041177	0.567783776
lncRNA	2410021H03Rik	0.041243	0.470590381
protein_coding	Foxp2	0.041276	0.645217659
protein_coding	Zfp583	0.041306	0.614489233
protein_coding	Rnf212	0.041416	0.464299825
protein_coding	Itgbl1	0.041705	0.575598252
TEC	Gm47173	0.041894	0.596156152
lncRNA	Gm32569	0.041942	0.513889803
lncRNA	Gm20667	0.041947	0.628933982
protein_coding	Spin4	0.042235	0.60105267
lncRNA	Gm28151	0.042267	0.657884305
protein_coding	Wnt16	0.042335	0.550004174
protein_coding	Calcr	0.042348	0.481932158
lncRNA	Gm11706	0.042631	0.556873547
protein_coding	Fxyd2	0.042685	0.453726799
protein_coding	Fndc1	0.042685	0.630386513
lncRNA	Gm35585	0.042872	0.561715411
TEC	Gm44508	0.043331	0.605966604
lncRNA	Gm28536	0.043416	0.434377392
TEC	Gm42440	0.043753	0.553414632
TEC	Gm42908	0.044055	0.57848046
misc_RNA	Gm24629	0.044446	0.451616641
TEC	Gm48961	0.045144	0.626787494
lncRNA	D330037F02Rik	0.045225	0.368713264
misc_RNA	Gm22136	0.045372	0.562744829
TEC	Gm43312	0.045372	0.625003268
protein_coding	Igf2bp2	0.045372	0.648801132
lncRNA	5730414N17Rik	0.04637	0.497545351
protein_coding	Gm7347	0.04646	0.329739149
protein_coding	Cd24a	0.04655	0.654500749
protein_coding	Dync1i1	0.046695	0.655567209

© 2023. Nandy A et al. Published online at http://www.aginganddisease.org/EN/10.14336/AD.2023.0510

lncRNA	Gm36787	0.046867	0.476543591
protein_coding	Prl2c3	0.046873	0.614873901
protein_coding	Galnt5	0.047271	0.577252176
protein_coding	Ramp3	0.047337	0.526207268
protein_coding	Prkg2	0.047523	0.537636098
protein_coding	Vkorc1	0.047567	0.531820096
processed_pseudogene	Gm44282	0.0479	0.607325879
protein_coding	Fmod	0.048028	0.574030634
processed_pseudogene	Gm49798	0.04836	0.615682425
protein_coding	Scube1	0.048662	0.641517956
unprocessed_pseudogene	Cyp2c52-ps	0.048666	0.416084691
protein_coding	Olfr419	0.048729	0.547818313
processed_pseudogene	Gm16236	0.048844	0.584856158
lncRNA	Gm14023	0.049041	0.619047575
processed_pseudogene	Gm20689	0.049113	0.472114444
protein_coding	Brinp3	0.049829	0.091495714
lncRNA	Gm6213	0.049834	0.413974178

2.3. Function wise differentially expressed genes

2.3.1. Oxidative Stress

Upregulated			
**Feature_gene_biotype**	**Feature_gene_name**	**padj**	**FoldChange**
protein_coding	Gadd45b	0.001491	1.593439744
protein_coding	Maob	0.0257	1.779212791
protein_coding	Ppargc1a	0.036354	1.837495074
protein_coding	Alox15	0.012886	1.869043523
protein_coding	Ros1	0.006438	4.950749426
protein_coding	Nfe2l1	0.000295	1.988026008
protein_coding	Acer2	0.000176	1.550827514

2.3.2. Lipid peroxidation responder

Downregulated			
**Feature_gene_biotype**	**Feature_gene_name**	**padj**	**FoldChange**
protein_coding	Aldh1l2	0.004097	0.621503827

Upregulated			
**Feature_gene_biotype**	**Feature_gene_name**	**padj**	**FoldChange**
protein_coding	Aldh5a1	0.022745	1.565166548
protein_coding	Aldh3b2	7.94E-05	1.733445825
protein_coding	Aldh1l1	0.002667	1.94420628
protein_coding	Aldh1a2	2.19E-14	5.029557771

© 2023. Nandy A et al. Published online at http://www.aginganddisease.org/EN/10.14336/AD.2023.0510

SUPPLEMENTARY DATA

2.3.3. Antioxidant

Downregulated			
**Feature_gene_biotype**	**Feature_gene_name**	**padj**	**FoldChange**
protein_coding	Gpx7	8.60E-07	0.444951542
protein_coding	Ddit4	0.001043	0.476489554
protein_coding	Pxdn	0.016827	0.649771914
protein_coding	Bdh2	0.018895	0.529521325

2.3.4. Glucose metabolism

Downregulated			
**Feature_gene_biotype**	**Feature_gene_name**	**padj**	**FoldChange**
protein_coding	Pklr	0.004658	0.625037073

Upregulated			
**Feature_gene_biotype**	**Feature_gene_name**	**padj**	**FoldChange**
protein_coding	Gys1	0.042693	1.567818963
protein_coding	Hk2	0.002882	1.742405356
protein_coding	Ogdhl	0.044304	1.915797581
protein_coding	Fbp2	0.020225	2.043145343
protein_coding	Pgm5	0.001754	2.15666437
protein_coding	Eno4	0.025461	2.222340889
protein_coding	Gys2	2.20E-05	7.067098391

2.3.5. Lipid metabolism

Downregulated			
**Feature_gene_biotype**	**Feature_gene_name**	**padj**	**FoldChange**
protein_coding	Lipc	1.29E-05	0.402365866
protein_coding	Twist1	0.000908	0.560979175

Upregulated			
**Feature_gene_biotype**	**Feature_gene_name**	**padj**	**FoldChange**
protein_coding	Pex11a	0.009335	1.521735358
protein_coding	Mlycd	0.034045	1.639375606
protein_coding	Acadm	0.01798	1.64105902
protein_coding	Plin5	0.008293	1.909437669
protein_coding	Acsbg1	0.001744	2.716570792
protein_coding	Dgat2l6	0.045996	3.819615488
protein_coding	Acsm5	0.012025	4.0135416
protein_coding	Apol11a	1.55E-58	9.068502656

protein_coding	Fgfbp3	8.88E-49	10.01275759
protein_coding	Apol10a	1.34E-07	10.98820172
protein_coding	Apol6	6.38E-06	2.29419149
protein_coding	Apol10b	0.006868	2.356101889
protein_coding	Apobr	0.009836	1.587660507
protein_coding	Lpl	0.039739	1.737908631

2.3.6. Cartilage morphogenesis

Downregulated			
**Feature_gene_biotype**	**Feature_gene_name**	**padj**	**FoldChange**
protein_coding	Col12a1	9.42E-22	0.180769184
protein_coding	Col6a1	2.84E-15	0.346987741
protein_coding	Col6a2	5.94E-14	0.372275576
protein_coding	Matn2	1.09E-10	0.443406312
protein_coding	Matn4	6.38E-06	0.461152152
protein_coding	Stc1	0.001097	0.486507984
protein_coding	Matn3	0.020809	0.127842084
protein_coding	Chst3	1.45E-10	0.584331526

Upregulated			
**Feature_gene_biotype**	**Feature_gene_name**	**padj**	**FoldChange**
protein_coding	Col7a1	0.00016	2.749247941

2.3.7. Chondrocyte differentiation

Downregulated			
**Feature_gene_biotype**	**Feature_gene_name**	**padj**	**FoldChange**
protein_coding	Acan	2.10E-08	0.1071021
protein_coding	Matn3	0.020809	0.12784208
protein_coding	Col12a1	9.42E-22	0.18076918
protein_coding	Loxl2	1.21E-24	0.19219916
protein_coding	Col11a1	1.12E-12	0.23197732
protein_coding	Ccn3	0.001628	0.26340417
protein_coding	Fgf18	6.21E-06	0.26507288
protein_coding	Col6a1	2.84E-15	0.34698774
protein_coding	Col6a2	5.94E-14	0.37227558
protein_coding	Serpinh1	2.18E-05	0.38819692
protein_coding	Matn2	1.09E-10	0.44340631
protein_coding	Sulf1	3.21E-06	0.45064249
protein_coding	Matn4	6.38E-06	0.46115215
protein_coding	Chst3	1.45E-10	0.58433153

© 2023. Nandy A et al. Published online at http://www.aginganddisease.org/EN/10.14336/AD.2023.0510

SUPPLEMENTARY DATA

Upregulated			
**Feature_gene_biotype**	**Feature_gene_name**	**padj**	**FoldChange**
protein_coding	Fgf9	3.69E-05	2.43762338
protein_coding	Efemp1	0.001218	2.44853057
protein_coding	Col7a1	0.00016	2.74924794
protein_coding	Bmpr1b	0.000754	2.93586446
protein_coding	Gdf6	0.001352	3.71174237

2.3.8. Osteoblast differentiation

Downregulated			
**Feature_gene_biotype**	**Feature_gene_name**	**padj**	**FoldChange**
protein_coding	Wnt7b	6.31E-12	0.11701256
protein_coding	Sox11	3.12E-05	0.1386813
protein_coding	Tnn	2.60E-14	0.17407271
protein_coding	Nell1	0.000341	0.195264129
protein_coding	Bglap2	0.000238	0.255919668
protein_coding	Bglap	0.002419	0.260881227
protein_coding	Fbn2	1.03E-14	0.264633305
protein_coding	Cthrc1	3.40E-06	0.301548074
protein_coding	Gja1	1.91E-09	0.312819396
protein_coding	Lrrc17	1.41E-13	0.330539632
protein_coding	Igfbp3	5.64E-13	0.381686708
protein_coding	Col1a1	0.00011	0.429890106
protein_coding	Sox4	0.00029	0.600857856

Upregulated			
**Feature_gene_biotype**	**Feature_gene_name**	**padj**	**FoldChange**
protein_coding	Esrra	0.019811	1.507194754
protein_coding	Ostn	0.045804	1.790767969
protein_coding	Fgf23	0.000749	2.047870557
protein_coding	Bglap3	0.000878	2.429262768
protein_coding	Fgf9	3.69E-05	2.437623384
protein_coding	Bmpr1b	0.000754	2.935864455
protein_coding	Trp63	5.64E-08	2.989408863
protein_coding	Gpnmb	4.13E-14	3.81051395
protein_coding	Shh	0.034331	15.19623438

2.3.9. Collagen synthesis and organization

Downregulated			
**Feature_gene_biotype**	**Feature_gene_name**	**padj**	**FoldChange**
protein_coding	Col9a1	1.13E-06	0.049857432
protein_coding	Col9a2	2.11E-06	0.086359065

© 2023. Nandy A et al. Published online at http://www.aginganddisease.org/EN/10.14336/AD.2023.0510

SUPPLEMENTARY DATA

protein_coding	Acan	2.10E-08	0.1071021
protein_coding	Col3a1	1.91E-28	0.155777902
protein_coding	Lum	4.54E-06	0.171031084
protein_coding	Col12a1	9.42E-22	0.180769184
protein_coding	Loxl2	1.21E-24	0.192199159
protein_coding	Col11a1	1.12E-12	0.231977321
protein_coding	Col10a1	0.007624	0.290841053
protein_coding	Col16a1	1.07E-14	0.305203637
protein_coding	Lox	2.50E-06	0.322207256
protein_coding	Col5a2	7.43E-10	0.326212268
protein_coding	Col8a1	1.19E-12	0.345737258
protein_coding	Col6a1	2.84E-15	0.346987741
protein_coding	Col6a3	3.49E-28	0.36196761
protein_coding	Col6a2	5.94E-14	0.372275576
protein_coding	Serpinh1	2.18E-05	0.388196923
protein_coding	Col5a1	8.89E-12	0.39455102
protein_coding	Col1a2	2.93E-05	0.407289648
protein_coding	Col26a1	0.006279	0.427886668
protein_coding	Col1a1	0.00011	0.429890106
protein_coding	Col15a1	2.11E-07	0.443007969
protein_coding	Col8a2	1.69E-05	0.447230096
protein_coding	Col4a1	3.38E-08	0.45899559
protein_coding	Plod2	0.007073	0.499926656
protein_coding	Col5a3	0.000219	0.533497229
protein_coding	Col4a2	0.00018	0.559218017
protein_coding	Col24a1	2.55E-05	0.566974421
protein_coding	Col11a2	0.015462	0.581907911
protein_coding	Loxl1	4.15E-06	0.591467315
protein_coding	Col14a1	0.019953	0.627105651
protein_coding	Col18a1	0.027975	0.679371498

**Upregulated**			
**Feature_gene_biotype**	**Feature_gene_name**	**padj**	**FoldChange**
protein_coding	Col4a3	0.000142	2.075324737
protein_coding	Col4a5	0.002359	2.222318421
protein_coding	Col7a1	0.00016	2.749247941

© 2023. Nandy A et al. Published online at http://www.aginganddisease.org/EN/10.14336/AD.2023.0510

SUPPLEMENTARY DATA

2.3.10. Extracellular matrix organization

Downregulated			
**Feature_gene_biotype**	**Feature_gene_name**	**padj**	**FoldChange**
protein_coding	Col9a1	1.13E-06	0.049857432
protein_coding	Acan	2.10E-08	0.1071021
protein_coding	Col3a1	1.91E-28	0.155777902
protein_coding	Lum	4.54E-06	0.171031084
protein_coding	Col12a1	9.42E-22	0.180769184
protein_coding	Loxl2	1.21E-24	0.192199159
protein_coding	Postn	1.80E-13	0.211861197
protein_coding	Col11a1	1.12E-12	0.231977321
protein_coding	Col10a1	0.007624	0.290841053
protein_coding	Col16a1	1.07E-14	0.305203637
protein_coding	Mmp13	0.005352	0.308556436
protein_coding	Egfl6	8.38E-05	0.317600617
protein_coding	Lox	2.50E-06	0.322207256
protein_coding	Col5a2	7.43E-10	0.326212268
protein_coding	Mfap4	0.000118	0.338859411
protein_coding	Col8a1	1.19E-12	0.345737258
protein_coding	Serpinh1	2.18E-05	0.388196923
protein_coding	Col5a1	8.89E-12	0.39455102
protein_coding	Ibsp	0.001878	0.397603393
protein_coding	Col1a2	2.93E-05	0.407289648
protein_coding	Aplp1	2.08E-06	0.4219458
protein_coding	Col1a1	0.00011	0.429890106
protein_coding	Col15a1	2.11E-07	0.443007969
protein_coding	Col8a2	1.69E-05	0.447230096
protein_coding	Sulf1	3.21E-06	0.450642492
protein_coding	Col4a1	3.38E-08	0.45899559
protein_coding	Smoc2	0.002225	0.467769122
protein_coding	Flrt2	0.000283	0.480744595
protein_coding	Scara3	5.36E-07	0.481334321
protein_coding	Olfml2b	1.43E-05	0.481571912
protein_coding	Pdpn	0.012963	0.48491978
protein_coding	Plod2	0.007073	0.499926656
protein_coding	Mmp14	4.21E-05	0.510594067
protein_coding	Col5a3	0.000219	0.533497229
protein_coding	Col4a2	0.00018	0.559218017
protein_coding	Col24a1	2.55E-05	0.566974421
protein_coding	Col11a2	0.015462	0.581907911
protein_coding	Loxl1	4.15E-06	0.591467315
protein_coding	Col14a1	0.019953	0.627105651

© 2023. Nandy A et al. Published online at http://www.aginganddisease.org/EN/10.14336/AD.2023.0510

SUPPLEMENTARY DATA

Upregulated			
**Feature_gene_biotype**	**Feature_gene_name**	**padj**	**FoldChange**
protein_coding	Col4a3	0.000142	2.07532474
protein_coding	Col4a5	0.002359	2.22231842
protein_coding	Elf3	4.59E-06	6.21833119

2.3.11. Ossification

Downregulated			
**Feature_gene_biotype**	**Feature_gene_name**	**padj**	**FoldChange**
protein_coding	Wnt7b	6.31E-12	0.11701256
protein_coding	Sox11	3.12E-05	0.1386813
protein_coding	Tnn	2.60E-14	0.17407271
protein_coding	Nell1	0.0003413	0.195264129
protein_coding	Col11a1	1.12E-12	0.231977321
protein_coding	Ptn	1.82E-10	0.232847176
protein_coding	Bglap2	0.0002378	0.255919668
protein_coding	Bglap	0.0024191	0.260881227
protein_coding	Fbn2	1.03E-14	0.264633305
protein_coding	Fgf18	6.21E-06	0.265072882
protein_coding	Col10a1	0.0076238	0.290841053
protein_coding	Cthrc1	3.40E-06	0.301548074
protein_coding	Mmp13	0.0053524	0.308556436
protein_coding	Gja1	1.91E-09	0.312819396
protein_coding	Col5a2	7.43E-10	0.326212268
protein_coding	Lrrc17	1.41E-13	0.330539632
protein_coding	Sparc	1.21E-05	0.345593721
protein_coding	Omd	0.0116982	0.355810414
protein_coding	Igfbp3	5.64E-13	0.381686708
protein_coding	Ibsp	0.0018776	0.397603393
protein_coding	Mmp2	2.55E-07	0.401460681
protein_coding	Igsf10	7.21E-05	0.411733658
protein_coding	Col1a1	0.0001101	0.429890106
protein_coding	Alpl	3.84E-05	0.44456637
protein_coding	Calcr	0.0423483	0.481932158
protein_coding	Stc1	0.001097	0.486507984
protein_coding	Tnfsf11	0.0038308	0.488375273
protein_coding	Clec11a	0.0024994	0.491207868
protein_coding	Mmp14	4.21E-05	0.510594067
protein_coding	Sox4	0.0002902	0.600857856
protein_coding	Adamts12	0.0006249	0.632956396

© 2023. Nandy A et al. Published online at http://www.aginganddisease.org/EN/10.14336/AD.2023.0510

SUPPLEMENTARY DATA

Upregulated			
**Feature_gene_biotype**	**Feature_gene_name**	**padj**	**FoldChange**
protein_coding	Esrra	0.019811	1.507194754
protein_coding	Ostn	0.045804	1.790767969
protein_coding	Ryr1	0.017085	2.027391894
protein_coding	Fgf23	0.000749	2.047870557
protein_coding	Bglap3	0.000878	2.429262768
protein_coding	Fgf9	3.69E-05	2.437623384
protein_coding	Bmpr1b	0.000754	2.935864455
protein_coding	Trp63	5.64E-08	2.989408863
protein_coding	Gpnmb	4.13E-14	3.81051395
protein_coding	Shh	0.034331	15.19623438

2.3.12. Bone development

Downregulated			
**Feature_gene_biotype**	**Feature_gene_name**	**padj**	**FoldChange**
protein_coding	Col9a1	1.13E-06	0.049857432
protein_coding	Matn3	0.020809	0.127842084
protein_coding	Col12a1	9.42E-22	0.180769184
protein_coding	Bglap2	0.000238	0.255919668
protein_coding	Bglap	0.002419	0.260881227
protein_coding	Fgf18	6.21E-06	0.265072882
protein_coding	Pax1	0.004068	0.289614867
protein_coding	Col10a1	0.007624	0.290841053
protein_coding	Mmp13	0.005352	0.308556436
protein_coding	Lrrc17	1.41E-13	0.330539632
protein_coding	Sparc	1.21E-05	0.345593721
protein_coding	Col6a1	2.84E-15	0.346987741
protein_coding	Col6a2	5.94E-14	0.372275576
protein_coding	Serpinh1	2.18E-05	0.388196923
protein_coding	Col1a1	0.00011	0.429890106
protein_coding	Chad	0.004874	0.438955534
protein_coding	Matn2	1.09E-10	0.443406312
protein_coding	Alpl	3.84E-05	0.44456637
protein_coding	Sulf1	3.21E-06	0.450642492
protein_coding	Matn4	6.38E-06	0.461152152
protein_coding	Stc1	0.001097	0.486507984
protein_coding	Tnfsf11	0.003831	0.488375273
protein_coding	Mmp14	4.21E-05	0.510594067
protein_coding	Adamts12	0.000625	0.632956396

© 2023. Nandy A et al. Published online at http://www.aginganddisease.org/EN/10.14336/AD.2023.0510

SUPPLEMENTARY DATA

Upregulated			
**Feature_gene_biotype**	**Feature_gene_name**	**padj**	**FoldChange**
protein_coding	Esrra	0.019811	1.50719475
protein_coding	Ostn	0.045804	1.79076797
protein_coding	Ryr1	0.017085	2.02739189
protein_coding	Pitx2	0.006321	2.42223929
protein_coding	Bglap3	0.000878	2.42926277
protein_coding	Col7a1	0.00016	2.74924794
protein_coding	Bmpr1b	0.000754	2.93586446
protein_coding	Wnt1	8.78E-05	3.41864372

© 2023. Nandy A et al. Published online at http://www.aginganddisease.org/EN/10.14336/AD.2023.0510
